# Telemedicine in adult intensive care: A systematic review of patient-relevant outcomes and methodological considerations

**DOI:** 10.1371/journal.pdig.0001126

**Published:** 2025-12-15

**Authors:** Tamara Pscheidl, Carina Benstoem, Kelly Ansems, Lena Saal-Bauernschubert, Anne Ritter, Ana-Mihaela Zorger, Karolina Dahms, Sandra Dohmen, Eva Steinfeld, Julia Dormann, Claire Iannizzi, Nicole Skoetz, Heidrun Janka, Maria-Inti Metzendorf, Carla Nau, Miriam Stegemann, Patrick Meybohm, Falk von Dincklage, Sven Laudi, Falk Fichtner, Stephanie Weibel

**Affiliations:** 1 University Hospital Würzburg, Department of Anaesthesiology, Intensive Care, Emergency and Pain Medicine, Würzburg, Germany; 2 Department of Intensive Care Medicine and Intermediate Care, Medical Faculty, RWTH Aachen University, Aachen, Germany; 3 Department of Infectious Diseases, Respiratory and Critical Care Medicine, Charité-Universitätsmedizin Berlin, Berlin Institute of Health, Berlin, Germany; 4 Institute of Public Health, Faculty of Medicine and University Hospital Cologne, University of Cologne, Cologne, Germany; 5 Institute of General Practice, Medical Faculty of the Heinrich-Heine-University Düsseldorf, Düsseldorf, Germany; 6 University Medical Center Schleswig-Holstein, Campus Lübeck, Department of Anaesthesiology and Intensive Care Medicine, Lübeck, Germany; 7 Universitätsmedizin Greifswald, Department of Anesthesia, Critical Care, Emergency and Pain Medicine, Greifswald, Germany; 8 Department of Anesthesiology and Intensive Care Medicine, University of Leipzig Medical Center, Leipzig, Germany; Iran University of Medical Sciences, IRAN, ISLAMIC REPUBLIC OF

## Abstract

Given the growing challenges of healthcare, including an aging population and increasing shortages of specialized intensive care staff, this systematic review investigates the efficacy of telemedicine in intensive care compared to standard of care (SoC) or any other type or mode of telemedicine on patient-relevant outcomes for adult intensive care unit (ICU) patients. This systematic review follows Cochrane’s methodological standards. Comprehensive searches for any controlled clinical studies were conducted in MEDLINE, Scopus, CINAHL, and CENTRAL (up to 18 April 2024, and an updated search for randomized controlled trials (RCTs) up to 29 September 2025). Twenty-six studies comparing telemedicine in intensive care to SoC with approximately 2,164,508 analysed patients were identified, including data from one cluster RCT (cRCT), two stepped-wedge cluster RCTs (sw-cRCTs), and 23 non-randomized studies of interventions (NRSIs). No other comparisons were identified. Due to high clinical and methodological heterogeneity among studies, no meta-analysis was conducted. For ICU mortality, one cRCT (15,230 patients) and two sw-cRCTs (5,915 patients) showed heterogeneous results: two found no evidence for a difference, while one favoured SoC (very low-certainty). One sw-cRCT (1,462 patients) reporting overall mortality at 180 days suggested no evidence for a difference between groups (very low-certainty). Data from one cRCT (15,230 patients) and one sw-cRCT (1,462 patients) on ICU length of stay (LOS) showed no evidence for a difference between groups (moderate- and very low-certainty). Quality of life from one sw-cRCT (786 patients) indicated no evidence for a difference (very low-certainty). Six NRSIs reported adjusted data on ICU mortality, two on overall mortality, and three on ICU LOS, with heterogeneous results. High risk of bias and substantial heterogeneity limited the certainty, emphasizing the need for robust, patient-centered research in clinical studies to define telemedicine’s role in intensive care and optimize its implementation. Future studies should particularly ensure transparent and comprehensive reporting.

## Author summary

Telemedicine is increasingly used in intensive care units (ICUs) around the world to provide remote medical expertise, especially in settings with limited staff or resources. However, it remains unclear how well telemedicine works in practice and which models are most effective for putting it into practice. In our systematic review, we carefully examined 26 studies involving over two million patients. We looked at patient-relevant outcomes such as mortality, length of ICU stay, and quality of life. We found that the results of these studies were highly variable and often limited by poor reporting, differences in study design, and lack of standardization. Because the studies were too diverse, we were unable to combine their results in meta-analyses. The variability across studies limited our ability to evaluate the effects of telemedicine in intensive care on patient-relevant outcomes or to determine which types of telemedicine models work best in practice. Our findings highlight the urgent need for well-designed, patient-centered studies that not only measure patient-relevant outcomes but also evaluate how telemedicine in intensive care settings should be structured and delivered. This will be key to ensuring that future telemedicine programs are both effective and tailored to the needs of patients and health professionals.

## Introduction

Telemedicine in intensive care has emerged as a promising solution to enhance quality of patient care by providing remote access to intensive care specialists. This is particularly important given the increasing demand for intensive care services due to a growing and aging population compared with the shortage of intensive care unit (ICU) professionals - a challenge that is becoming increasingly significant in Germany [1–3].

Telemedicine experts can operate either individually or as part of interdisciplinary and multiprofessional clinical teams, utilizing various audio-, audio-visual-, and data transfer technologies to deliver optimal, evidence-based care to ICU patients, regardless of time or location. Telemedicine is a complex intervention, ensuring data security, enabling teaching, and coordinating care across multiple healthcare providers, all while addressing regulatory and logistical challenges. Therefore, several questions remain about the optimal approach for implementing and conducting telemedicine services, including which conditions or situations in the ICU setting benefit most from telemedical consultation or treatment to finally achieve beneficial effects and reduce harm for critically ill patients.

Until today, telemedicine programs investigated in clinical studies have demonstrated mixed effects on patient-relevant outcomes when combined in systematic reviews with meta-analysis ranging from reduced mortality and length of stay (LOS) to no effect [4–7]. These inconsistencies are probably related to high heterogeneity among clinical studies, e.g., regarding variations in the design of telemedicine interventions and differences in the patient populations analysed, as well as different review methods.

We conducted a systematic review following Cochrane standards to address the methodological challenges presented by the heterogeneous study landscape. This systematic review is part of an evidence-based German Arbeitsgemeinschaft der Wissenschaftlichen Medizinischen Fachgesellschaften e.V. (AWMF) S3-guideline on telemedicine in intensive care [8] and was conducted to provide most up to date recommendations regarding the optimal technical implementation of telemedicine to improve patient-relevant outcomes in modern healthcare. Therefore, this systematic review compares telemedicine in intensive care to standard of care (SoC), as well as to other telemedicine types or modes to assess ICU mortality, overall mortality at longest follow-up, ICU LOS, and quality of life for adult ICU patients. Additionally, the review aims to identify evidence gaps and should guide future research to optimize the rationale and design of telemedicine studies.

## Materials and methods

The protocol for this systematic review was registered within the International Prospective Register of Systematic Reviews (PROSPERO, registration number CRD42024547985) and was publicly accessible on 28 May 2024 (S1 Protocol). This systematic review was conducted in accordance with the PRISMA 2020 checklist (S1 Checklist).

### Eligibility criteria and search

#### Types of studies.

We included any controlled study design. Although randomized controlled trials (RCTs) represent the most valid study design to investigate the efficacy of interventions, only a few studies regarding telemedicine in intensive care have been conducted in this format so far. Therefore, we considered controlled non-randomized studies of interventions (NRSIs) eligible in order to extend the evidence base. We considered results reported as full-text journal publication, preprint article, and results published in trial registries. We restricted our search to reports in English or German due to the practical constraint of language fluency among the review authors which ensures accurate assessment of study content. Furthermore, studies must have been published from 1999 onwards, as telemedicine became widely available from that time and to ensure consistency with modern technical standards. Studies must have included ten or more participants to increase the validity and generalizability of the findings.

#### Types of participants.

Studies investigating any critically ill adult (≥ 18 years) inpatient on any ICU or critical care unit (CCU) were eligible. We excluded studies investigating children (< 18 years), and non-ICU and emergency department (ED) patients.

#### Types of interventions and comparators.

Studies comparing telemedicine to SoC defined as care without telemedicine in any ICU setting, or studies comparing telemedicine to any other type or mode of telemedicine were considered. We defined telemedicine as a standardized audio- or audio- and video-connection delivering care by using high-tech (e.g., specific communication technology for telemedical purpose, i.e., remote-controlled camera in the patient’s room) or low-tech (e.g., laptop, mobile) equipment, optionally in combination with shared electronic health records (EHR) with automated data transfer or without automated data transfer. Telemedicine in intensive care was defined as any telemedicine delivered by ICU professionals (e.g., by daily rounding or contact on demand) practicing in health-care institutions or tele-centers, to ICU professionals located elsewhere in ICU settings. Additionally, telemedicine had to include an assessment of all organ systems.

Eligible comparisons for this review are:

- Telemedicine in intensive care vs SoC
- Any type of telemedicine vs any other type of telemedicine in intensive care
- Any mode of telemedicine vs any other mode of telemedicine in intensive care.

#### Types of outcome measures.

Our main outcome set included ICU mortality, overall mortality at longest follow-up, ICU LOS, and quality of life at longest follow-up. Additional outcomes were hospital mortality, hospital LOS, disease-related detection rate (e.g., correctly diagnosed disease), disease-specific effects (e.g., adequate antibiotic therapy, antibiotic consumption, ventilation, positioning), transfer rate (e.g., from telemedicine recipient to other clinics, e.g., telemedicine provider), acceptance (e.g., patient, family, care givers), adherence to best practice guidelines (e.g., sepsis management, lung protective ventilation), fulfilment of process and quality indicators (e.g., start of enteral nutrition, start of antibiotic treatment, daily interdisciplinary visits), change of therapeutic goal, and triage result.

#### Review team.

The review team consists of methodological experts (TP, CB, KA, LSB, AMZ, KD, ES, JD, CI, NS, HJ, MIM, SW) and clinical telemedicine experts in ICU settings (AR, SD, CN, MS, PM, FvD, SL, FF). Clinical experts supported decisions regarding study selection and interpretations of clinical relevance of estimated effects.

#### Systematic search.

Systematic searches were conducted on 9 January 2024 and 18 April 2024, the first focusing on telemedicine and ICUs, the second on telemedicine and acute diseases. The following bibliographic databases were searched from inception until 9 January or 18 April 2024: Ovid MEDLINE, Scopus, CINAHL, and Cochrane Central Register of Controlled Trials (CENTRAL), for studies focusing on telemedicine in ICUs or acute diseases, respectively. In addition, we searched the following trials registries to identify completed, unpublished and ongoing studies: ClinicalTrials.gov and WHO International Clinical Trials Registry Platform (ICTRP). We also searched reference lists of included studies and systematic reviews. Two update searches of both preliminary searches focusing only on RCTs were conducted on 26 October 2024 and 29 September 2025 in MEDLINE and CENTRAL. The full search strategy is reported in the supplement (S1 Text).

#### Selection of studies.

Two review authors independently performed study selection in Covidence (https://www.covidence.org) according to predefined eligibility criteria in accordance with the *Cochrane Handbook for Systematic Reviews of Interventions* [9]. The review authors screened titles and abstracts of identified records. Disagreements were resolved by discussion and in case of doubt, the study was carried over to the full-text screening stage. Two review authors independently assessed eligibility of full-text records. Disagreements between two review authors were solved by discussion or by consulting a third review author. When more than one article presented data on the same population, the article with the largest number of subjects included or with the most informative data was chosen.

#### Role of the funding source.

The funder of this systematic review had no role in study design, data collection, data analysis, data interpretation, or writing of the report.

### Data collection and analysis

#### Data extraction.

Two review authors independently extracted general study data as detailed in the protocol, including details on predefined study characteristics, settings, participants, intervention- and comparator details in Covidence (https://www.covidence.org), and outcome data in Excel (https://office.microsoft.com/excel) using piloted data extraction forms. Based on the excel sheet, it was determined which studies were eligible for inclusion in each comparison. For cluster RCTs (cRCTs), outcomes adjusted for cluster effects were extracted and for stepped-wedge cluster RCTs (sw-cRCTs), outcomes additionally adjusted for time trends were extracted. We did not consider outcome data that may have been overadjusted (e.g., for disease severity or age) for our main outcomes set since such adjustments could bias effect estimates. In a rigorously designed and properly blinded RCT, adjustment for such baseline confounders should not be necessary, as randomisation is expected to ensure baseline comparability between groups. For NRSIs, only adjusted outcome data, e.g., adjusted for disease severity or age, were extracted. At each step of data extraction, we resolved discrepancies by discussion within the team. In case of missing data, we contacted the study authors via e-mail.

#### Assessment of risk of bias in included studies.

Two review authors independently assessed the risk of bias for each relevant main outcome reported in the included studies. For cRCTs, outcomes adjusted for cluster effects were assessed using the Cochrane Risk of Bias tool for cRCTs (RoB 2) [10]. For sw-cRCT, outcomes adjusted for cluster effects and time trends were assessed using the RoB 2 tool [10]. Data from sw-cRCTs not adjusted for time trends were considered for risk of time trend bias according to recommendations in the Cochrane Handbook [11]. For NRSIs, outcomes (only adjusted data, e.g., adjusted for disease severity or age) were rated using the Risk Of Bias In Non-randomized Studies - of Interventions (ROBINS-I) tool [12]. For each domain, studies and outcomes were classified as ‘low’, ‘some concerns’, or ‘high’ risk of bias (for RoB 2) or as ‘low’, ‘moderate’, ‘serious’, or ‘critical’ risk of bias (for ROBINS-I) according to the instructions of the tools. The review authors resolved disagreements by discussion with the team.

#### Data synthesis.

We planned to perform meta-analyses according to recommendations from the Cochrane Handbook [9]. We did not pool data from RCTs and NRSIs as well as from sw-cRCTs and cRCTs due to incompatible study designs. If clinical and methodological characteristics of individually identified studies with comparable study designs were sufficiently similar, we planned to pool data. Owing to clinical and methodological heterogeneity among all included studies, we decided against pooling and did not conduct any meta-analyses in this systematic review. Results were therefore reported descriptively and compiled in a Summary of Findings table.

Deviating from the protocol, only adjusted data from NRSIs were considered eligible for primary analysis due to increased validity without randomization, while data adjusted for cluster effects were used from cRCTs, and data adjusted for cluster effects and time trends were used from sw-cRCTs, preferably. For data lacking the cluster effect adjustment, we calculated the cluster effects using the studies’ primary data and an intracluster correlation coefficient (ICC), as recommended in the Cochrane Handbook [13]. The effective sample size was calculated with assumption of an ICC estimate (ICC = 0.018) from a similar study Ukoumunne *et al* [14]. This adjustment accounts for the design effect introduced by clustering.

Risk ratio (RR) with 95% confidence intervals (CIs) was the preferred effect measure for meta-analysis of binary outcomes. Since several adjusted binary effect estimates in NRSIs were reported as adjusted odds ratios (ORs), ORs with 95% CIs were also extracted. For continuous outcomes, mean difference (MD) was the preferred effect measure [15].

We considered effect estimates of dichotomous outcomes with the range of the 95% CIs not crossing 1 and continuous outcomes with the range of the 95% CIs not crossing 0 as statistically significant (e.g., favoured/did not favour the intervention), or statistically not significant (e.g., no evidence for a difference). A statistically significant effect does not necessarily imply that the estimated effect is clinically relevant. Clinical relevance was assessed by experts in the field.

Statistical heterogeneity was intended to be assessed using the χ² test and the I² statistic, and the 95% prediction interval for random-effects meta-analyses, as prespecified in the protocol. As we did not pool any studies, we compared the point estimates and the 95% CIs of studies to assess heterogeneity. Heterogeneity was planned to be explored via subgroup analysis as outlined in the protocol. However, none of the subgroup analyses could be conducted due to lack of meta-analyses, insufficient reporting of participant characteristics, and missing diversity in intervention details.

Due to high risk of selection bias in sw-cRCTs, we narratively compared overadjusted outcome data (data from RCTs adjusted for, e.g., age or disease severity) with data adjusted for cluster effects only to evaluate robustness of effect estimates.

There are many potential sources of missing data in a systematic review or meta‐analyses, which can affect the level of studies, outcomes, summary data, individuals, or study‐level characteristics. Incomplete data can introduce bias into systematic reviews and meta‐analysis, if they are not missing at random. We planned to address all sources of missing data. Missing studies may be the result of reporting bias. We searched for completed non‐published trials in trial registers. We classified these studies as ‘awaiting classification’ until the results are reported. We reported the number of completed non‐published studies. Additionally, if there were 10 or more relevant studies pooled in a meta‐analysis, we planned to investigate risk of reporting bias (publication bias) in pairwise meta‐analyses using contour‐enhanced funnel plots. However, we did not pool any studies. Missing outcomes and summary data may be the result of selective reporting bias; missing individuals may be the result of attrition from the study or lack of intention‐to‐treat analysis. We addressed these sources of missing data using risk of bias assessment tools. If data were incompletely reported, we contacted the study authors to request additional information.

#### Certainty of evidence.

We used the Grading of Recommendations Assessment, Development and Evaluation (GRADE) approach (https://www.gradeworkinggroup.org) within the MagicApp (https://app.magicapp.org/) to assess the certainty of the evidence for all main outcomes. The GRADE assessment comprises the categories study risk of bias, inconsistency, indirectness of study results, imprecision, and publication bias and can result in one of four levels of certainty (‘very low’, ‘low’, ‘moderate’ or ‘high’). Clinical relevance of effect estimates was assessed by experts in the field. We conveyed findings using informative statements as outlined by Santesso *et al* [16].

## Results

### Search

**Fig 1:**
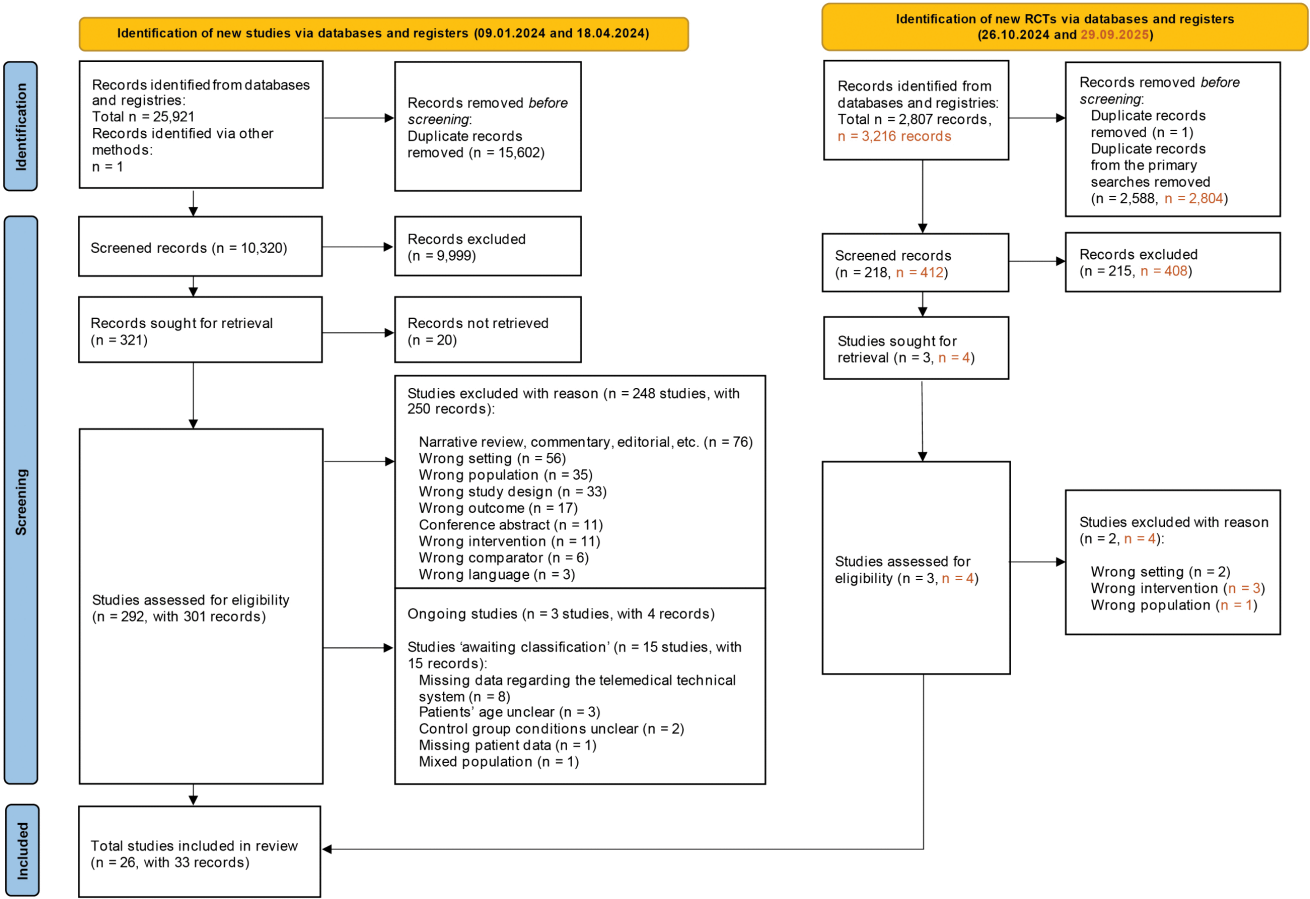
PRISMA flow diagram. Results of the searches are presented in the Preferred Reporting Items for Systematic reviews and Meta-Analyses (PRISMA) flow diagram. **Abbreviations:** Randomized controlled trial (RCT).

The result of the search is presented in the Preferred Reporting Items for Systematic reviews and Meta-Analyses (PRISMA) flow diagram (Fig 1). A total of 25,921 records were identified from the two initial searches. After removing duplicates, 10,320 records were screened and 9,999 were considered irrelevant. From the remaining 321 records, 20 were not retrievable as full-text articles despite intensive use of interlibrary loan. Three-hundred-one records were assessed for eligibility by full-text screening leading to the exclusion of 250 records with reasons. Three studies (non-RCTs and <1.000 patients) were excluded due to language restriction. Three studies were classified as ‘ongoing’ (none of them completed) and 15 studies remained ‘awaiting classification’ due to insufficient information on eligibility criteria. A search update for RCTs on 26 October 2024 identified further 218 records, from which one study was deemed eligible after screening. A second update search for RCTs conducted on 29 September 2025 identified 408 additional records; however, none were eligible for inclusion. Finally, 26 studies were included in this systematic review [17–49]. References of studies ongoing, excluded, and ‘awaiting classification’ along with reasons are presented in Supplement (S1 Table, S2 Table, S3 Table).

### Study characteristics and settings

One study utilized a traditional cRCT design, in which ICUs were randomized to either the telemedicine intervention or SoC group [41]. Two cRCTs used a stepped-wedge design, in which all ICU clusters transitioned from a control phase to the telemedicine phase [31,45]. Randomization determined the timing of each ICU’s transition. The majority of the 23 non-randomized studies used a before-and-after study design (n = 18). Additionally, four cohort studies were included [20,38,43,47], and one case-control study [27] (Table 1). Twenty-one studies were conducted in the United States, two in Germany [31,45], one in Australia [39], one in Brazil [41], and one did not report the country of study conduct [23] (Table 1). Seven out of 26 studies did not report a funding source at all, while three studies were fully or partially funded by industry, and the remaining studies by government, department, or a foundation (Table 1). All included studies compared telemedicine in intensive care to SoC without telemedicine (Table 1); however, in most studies SoC description was insufficient or completely absent. In 11 studies tele-centers provided telemedicine exclusively while in seven studies one or more hospitals acted as telemedicine provider. In three studies a combination of both was reported and in five studies no telemedicine provider information was available (Table 1). Recipient hospitals in eligible studies were community/rural (n = 5), urban/tertiary (n = 3), or university hospitals (n = 2). Mostly, a combination of different hospital types was reported (n = 13) and in three studies no recipient information was available (Table 1). Many hospitals used high-tech audio-video communication tools and EHR with automated data transfer (n = 15), while only three studies reported the use of low-tech equipment along with other approaches (n = 8) (S4 Table). The mode of communication varied widely across included studies, ranging from unstructured interviews during contact on demand only (n = 1) to combinations of contact on demand, emergency contact, together with daily rounding (n = 5), as well as structured interviews during contact on demand and daily rounding (n = 2), among other sorts of variations (n = 18) (S4 Table). Reporting on the expertise in telemedicine of the provider or recipient, including additional training or implementation aids, was generally inadequate (S4 Table). In most included studies, telemedical consultation combined with therapeutic decision making was the primary mode of delegation for remote caregivers (n = 12), followed by consultation only (n = 7), and therapeutic decision making only (n = 1) (S4 Table). Four studies did not report this information and two had additional regulations. Further information on telemedical technical equipment, mode of communication, and delegations grade are provided in Supplement (S4 Table). Twenty-four of the 26 included studies comprised 2,164,508 analysed patients, with a median (range) number of 7,314 (525-1,123,563) (Table 1). One NRSI reported the amount of telemedical consultations only [21]. Twenty of the included studies reported a mean or median age of > 60 years for the patient population, while two studies did not report the populations’ age at all (S5 Table). Most of the studies included a higher proportion of men than women; however, the difference was not significant within individual studies (S6 Table). Three of the included studies utilized an overlapping pool of study participants in their analyses: one with 6,988 enrolled patients [34], another with 563,491 enrolled patients [24], and a third without reporting the number of enrolled patients [38] (Table 1). Notably, more than 90% of the participants in this specific pool were men [24,34,38] (S6 Table). To avoid duplication, only one study [34] was used for further analyses.

**Table 1:** Study characteristics of included studies.

Study ID	Study design	Location	Funding	Hospital category provider	Hospital category recipient	Setting and patient status	Enrolled/ analysed participants (intervention/comparator)	Intervention/comparator	Reported relevant outcomesa
Boyle 2023	Before-and-after NRSI	United States	Industry; Foundation	Tele-center	Urban/tertiary	Adults; no specific disease (any critically ill patient)	15,114/14,908 (12,479/2,429)	Telemedicine/SoC	Hospital mortality, hospital LOS, transfer rated
Breslow 2004	Before-and-after NRSI	United States	Industry	Tele-center	Urban/tertiary	Adults, NR	2,144/2,140 (744/1,396)	Telemedicine/SoC	ICU mortalityd, hospital mortalityd, ICU LOSd, hospital LOSd
Collins 2017	Cohort NRSI	United States	Departmental	Urban/tertiary; university; tele-center	Urban/tertiary; University	Adults; surgical	NR/7,689 (1,037/6,652)	Telemedicine/SoC	ICU mortalityd, ICU LOSd
Davis 2017	Before-and-after NRSI	United States	NR	Military hospital	Military base/ rural	Adults; NR	NR/NR (NR/NR)	Telemedicine/SoC	ICU mortalityd, ICU LOSc, hospital LOSd, transfer rated, disease specific effectsd
Forni 2010	Before-and-after NRSI	NR	NR	Tele-center	Urban/tertiary	Adults; no specific disease (any critically ill patient)	NR/2,152 (1,073/1,079)	Telemedicine/SoC	Disease specific effectsc, ICU LOSd
Fortis 2014	Before-and-after NRSI	United States	NR	University; tele-center	University; 5 of 6 hospitals NR	Adults; NR	NR/12,160 (6,063/6,097)	Telemedicine/SoC	ICU mortalityd
Fortis 2018b	Before-and-after NRSI	United States	Government, federal; departmental	Tele-center	NR	Adults; no specific disease (any critically ill patient)	563,491/553,523 (97,256/456,267)	Telemedicine/SoC	Overall mortality at 30 daysc, ICU LOSd, transfer rated
Kahn 2016	Case-control NRSI	United States	Government, federal	NR	Urban/tertiary; community/rural; academic small teaching/ large teaching hospital	Adults; no specific disease (any critically ill patient)	NR/1,123,563 (292,636/830,927)	Telemedicine/SoC	Overall mortality at 90 days, ICU LOSd, hospital mortalityd
Lilly 2011	Before-and-after NRSI	United States	Departmental	University	University	Adults; no specific disease (any critically ill patient)	6,465/6,290 (4,761/1,529)	Telemedicine/SoC	Hospital mortality, ICU mortality, hospital LOS, ICU LOS, adherence to best practice guidelinesd
Lilly 2014	Before-and-after NRSI	United States	Departmental	NR	Urban/tertiary; community/rural; university	Adults; no specific disease (any critically ill patient)	119,169/118,990 (107,432/11,558)	Telemedicine/SoC	ICU mortality, hospital mortality
Lilly 2017	Before-and-after NRSI	United States	Departmental	University	University	Adults; no specific disease (any critically ill patient)	52,322/51,203 (36,946/14,257)	Telemedicine/SoC	Hospital LOSd
Marx 2022	Sw-cRCT	Germany	Government, federal	University	Urban/tertiary; community/rural	Adults; no specific disease (any critically ill patient)	159,424/36,790 (29,671/7,119)	Telemedicine/SoC	ICU LOSc, hospital LOSd, hospital mortality, ICU mortality, transfer rate, adherence to best practice guidelines, disease specific effects
McCambridge 2010	Before-and-after NRSI	United States	Grant	NR	Community/rural	Adults; no specific disease (any critically ill patient)	2,000/1,913 (959/954)	Telemedicine/SoC	ICU LOSd, hospital LOSd; ICU mortalityd
Morrison 2010	Before-and-after NRSI	United States	Foundation	Tele-center	Community/rural	Adults; no specific disease (any critically ill patient)	4,388/4,088 (2,717/1,371)	Telemedicine/SoC	ICU mortalityd, hospital mortalityd, ICU LOSd, hospital LOSd
Nassar 2014b	Before-and-after NRSI	United States	Government, federal; award	Tele-center	Urban/tertiary; community/rural; university	Adults; no specific disease (any critically ill patient)	6,988/6,939 (3,355/3,584)	Telemedicine/SoC	ICU mortality, hospital mortality, overall mortality at 30 days, ICU LOSc, hospital LOS
O’Shea 2022b	Cohort NRSI	United States	Departmental	Tele-center	NR	Adults; no specific disease (any critically ill patient)	NR/NR (81,333/235,670)	Telemedicine/SoC	Hospital mortality, ICU mortalityc, transfer rate, hospital LOS, ICU LOSc
Panlaqui 2017	Before-and-after NRSI	Australia	NR	Regional facility	NR	Adults; no specific disease (any critically ill patient)	541/525 (188/337)	Telemedicine/SoC	ICU mortality, hospital mortality, ICU LOSd, hospital LOSd, transfer rate
Pannu 2017	Before-and-after NRSI	United States	Government, federal	Tele-center	Community/rural	Adults; no specific disease (any critically ill patient)	19,389/18,292 (6,291/12,001)	Telemedicine/SoC	Hospital mortalityd, ICU LOSd, hospital LOSd, transfer rated
Pereira 2024	cRCT	Brazil	Government, federal	Tele-center	Community/rural; public hospitals (philanthropic and governmental administration)	Adults; no specific disease (any critically ill patient)	17,342/17,024 (15,230/1,794)	Telemedicine/SoC	ICU LOS, hospital mortality, disease specific effects, ICU mortality
Rosenfeld 2000	Cohort NRSI	United States	NR	At home	Academic-affiliated community hospital	Adults; no specific disease (any critically ill patient)	692/628 (201/427)	Telemedicine/SoC	Hospital mortalityd, hospital LOSd, ICU mortalityd, ICU LOSd
Sadaka 2013	Before-and-after NRSI	United States	NR	NR	Community/rural	Adults; no specific disease (any critically ill patient)	NR/2,823 (2,193/630)	Telemedicine/SoC	ICU mortality, hospital mortality, ICU LOSc, hospital LOS
Spies 2023	Sw-cRCT	Germany	Government, federal	University; tele-center	Community/rural; university	Adults; no specific disease (any critically ill patient)	1,463/1,462 (1,048/414)	Telemedicine/SoC	Overall mortality at 180 days, ICU LOS, ICU mortality, quality of life at 6 months, disease specific effects, fulfilment of process and quality indicators
Thomas 2009	Before-and-after NRSI	United States	Government, federal	Tele-center	Urban/tertiary; community/rural	Adults; NR	4,167/4,142 (2,108/2,034)	Telemedicine/SoC	ICU mortality, hospital mortalityd, ICU LOSd, hospital LOSd
Udeh 2022	Cohort NRSI	United States	Departmental	NR	Urban/tertiary; community/rural	Adults; no specific disease (any critically ill patient)	642,123/151,780 (107,930/43,850)	Telemedicine/SoC	Hospital mortalityd, hospital LOSd, ICU LOSd
VanGent 2018	Before-and-after NRSI	United States	NR	Urban/tertiary	Community/rural	Adults, pediatric, neonate; surgical ICU patients	NR/828 (513/315)	Telemedicine/SoC	ICU mortalityd, ICU LOSd, transfer rated
Willmitch 2012	Before-and-after NRSI	United States	Industry, departmental	Tele-center	Community/rural; partially NR	Adults; NR	NR/24,656 (18,152/6,504)	Telemedicine/SoC	Hospital mortality, hospital LOS, ICU LOS

**Abbreviations:** Cluster randomized controlled trial (cRCT), intensive care unit (ICU), length of stay (LOS), non-randomized study of intervention (NRSI), not applicable (NA), not reported (NR), standard of care (SoC), stepped-wedge cluster randomized controlled trial (sw-cRCT).

**Footnotes:**

^a^For analyses, we only used appropriately adjusted outcomes from our predefined outcomes set:

• adjusted outcomes for NRSIs.

• outcomes adjusted for time trends and cluster effects for sw-cRCTs.

• outcomes adjusted for cluster-effects for cRCTs.

• if relevant primary outcomes in (sw-)cRCTs were not adjusted for cluster effects, we performed the adjustment ourselves.

• outcomes adjusted for, e.g., age and disease severity for (sw-)cRCTs contributing to our secondary outcome set.

^b^Studies used the same population pool for analyses.

^c^Data could not be used for further analyses due to missing data, wrong data, or inappropriate effect measure.

^d^Data were not or inappropriately adjusted.

Three RCTs, one with cluster design and two with stepped-wedge cluster design, reported data of our main outcome set used in this systematic review [31,41,45]. Spies 2023 [45] reported ICU mortality, overall-mortality at 180 days, quality of life at 180 days, and ICU LOS. Marx 2022 [31] reported ICU mortality, and Pereira 2024 [41] ICU mortality and ICU LOS. Additionally, eight NRSIs reported outcomes of our main outcome set. ICU mortality was analysed by seven studies [28,29,34,39,44,46]. Three studies reported ICU LOS [28,34,49], and two studies overall mortality at 30 and 90 days [27,34]. Adjustments for different confounders are shown in Tables 2 and 3.

**Table 2:** Summary of findings: Telemedicine vs SoC in ICU patients ((sw-)cRCTs).

Outcome	Included studies: number of patients analysed for this outcome	Narrative synthesis		Certainty of evidence
**ICU mortality**	Marx 2022: 4,453^a^	RR 2.29 (95% CI 1.51 – 3.48)^1^; OR 1.28, 95% CI 0.91 – 1.79)^2^	We did not combine data from different studies owing to clinical/methodological heterogeneity. Evidence from Marx 2022 favoured SoC, Spies 2023 showed no evidence for a difference between groups.	Very low (due to very serious risk of bias, due to serious inconsistency)
	Spies 2023: 1,462^b^	RR 0.89 (95% CI 0.54 – 1.46)^3^	Pereira 2024 showed no evidence for a difference between groups.	Very low (due to extremely serious imprecision)
	Pereira 2024: 15,230^c^	RR 1.09 (95% CI 0.94 – 1.26)^4^	Spies 2023 showed no evidence for a difference between groups.	Very low (due to very serious risk of bias, due to serious imprecision)
**Overall mortality at 180 days**	Spies 2023: 1,462^b^	RR 1.02 (95% CI 0.76 – 1.36)^5^		
**Quality of life at 6 months on an EQ-5D-5L VAS scale: 0 – 100, higher is better**	Spies 2023: 786	MD -2.71 (95% CI 6.95 fewer to 1.53 more)^6^	Spies 2023 showed no evidence for a difference between groups.	Very low (due to very serious risk of bias, due to serious imprecision)
**ICU LOS**	Spies 2023: 1,462^b^	Median [IQR] 6 [4–13] days (telemedicine) vs 5 [3–11] days (SoC)^7^	Spies 2023 showed no evidence for a difference between groups.	Very low (due to very serious risk of bias, due to serious imprecision)
	Pereira 2024: 15,230^c^	Mean (SD) 8.1 (10) days (telemedicine) vs 7.1 (9) days (SoC); MD 1 day more, 95% CI 0.04 fewer to 1.96 more^7^	Pereira 2024 showed no evidence for a difference between groups.	Moderate (due to serious risk of bias)

**Abbreviations:** Cluster randomized controlled trial (cRCT), confidence interval (CI), European Quality of Life 5 Dimensions 5 Level Version (EQ-5D-5L), intensive care unit (ICU), interquartile range (IQR), length of stay (LOS), mean difference (MD), risk ratio (RR), standard deviation (SD), standard of care (SoC), stepped-wedge cluster randomized controlled trial (sw-cRCT).

**Footnotes:**

^a^Effective sample size: 1,334 from a sw-cRCT calculated by review authors.

^b^Effective sample size: 636 from a sw-cRCT calculated by review authors.

^c^Effective sample size: 1,505 from a cRCT calculated by review authors.

^1^Adjusted for cluster effect (by review authors). Extracted data: Number of events in intervention group 175/1,782, number of events in control group 113/2,671.

^2^Adjusted for hospital, age, and disease severity as reported in the study.

^3^Adjusted for cluster effect (by review authors). Extracted data: Number of events in intervention group 104/1,048, number of events in control group 45/414.

^4^Adjusted for cluster effect (by review authors). Extracted data: Number of events in intervention group 2,565/7,471, number of events in control group 2,435/7,759.

^5^Adjusted for cluster effect (by review authors). Extracted data: Number of events in intervention group 278/1,048, number of events in control group 107/414.

^6^Adjusted for cluster effect and time trends (by study authors after e-mail request).

^7^Unadjusted data as reported in the study.

**Table 3:** Summary of findings: Telemedicine vs SoC in ICU patients (NRSIs).

Outcome	Included studies: number of patients	Narrative synthesis		Certainty of evidence
ICU mortality	Lilly 2011: 6,290^a^	OR 0.37 (95% CI 0.28 – 0.49)	We did not combine data from different studies owing to clinical/methodological heterogeneity. Evidence from Lilly 2011, Lilly 2014, Sadaka 2013 favoured telemedicine; Nassar 2014, Thomas 2009, Panlaqui 2017 showed no evidence for a difference between groups.	Very low (due to very serious risk of bias, due to serious inconsistency)^1^
	Lilly 2014: 118,990^b^	HR 0.74 (95% CI 0.68 – 0.79)		
	Nassar 2014: 3,355^c^	OR 1.07 (95% CI 0.60 – 1.91)		
	Sadaka 2013: 2,823^d^	OR 0.46 (95% CI 0.32 – 0.66)		
	Thomas 2009: 4,142^e^	RR 0.88 (95% CI 0.71 – 1.08) OR 0.87 (95% CI 0.70 – 1.09)		
	Panlaqui 2017: 525^f^	RR 0.60 (95% CI 0.10 – 3.10)		
Overall mortality at longest follow-up (30 and 90 days)^b^	Nassar 2014 (30 days): 3,355^c^	OR 1.10 (95% CI 0.82 – 1.47)	We did not combine data from different studies owing to clinical/methodological heterogeneity. Evidence from Kahn 2016 favoured SoC; Nassar 2014 showed no evidence for a difference between groups.	Very low (due to very serious risk of bias, due to serious imprecision)^2^
	Kahn 2016 (90 days): 292,636^g^	RR 1.04 (95% CI 1.03 – 1.06)		
ICU LOS	Lilly 2011: 6,290^a^	HR 1.26 (95% CI 1.17 – 1.36)	We did not combine data from different studies owing to clinical/methodological heterogeneity. Evidence from Willmitch 2012 and Lilly 2011 favoured telemedicine; Nassar 2014 showed no evidence for a difference between groups.	Very low (due to very serious risk of bias, due to serious inconsistency)^3^
	Nassar 2014: 3,355^c^	OR 1.02, (95% CI 0.95 – 1.11)		
	Willmitch 2012: 12,285^h^	MD 0.56, (95% CI 0.36 – 0.76)		
Quality of life at 6 months on an EQ-5D-5L VAS scale: 0 – 100, higher is better	No NRSI reported this outcome.			

**Abbreviations:** Confidence interval (CI), hazard ratio (HR), intensive care unit (ICU), length of stay (LOS), mean difference (MD), non-randomized study of intervention (NRSI), odds ratio (OR), risk ratio (RR), standard of care (SoC).

**Footnotes:**

^a^adjusted for differences in acuity score, admission source, admission ICU, time after enrollment of first case in group, and other predictive factors including laboratory values and physiological measurements. ICU mortality was additionally adjusted for adherence to best practices and lower rates of complications.

^b^adjusted for APACHE IV score, age, hospital or ICU identifier (as a random effect), admission source, primary admission diagnosis, operative status, time from start of study enrollment, heart rate, admission and highest creatinine values, respiratory rate, admission hematocrit value, BUN, WBC count, Glasgow Coma Score, prothrombin time, anion gap, urine output (in the first 24 h), base excess, and total bilirubin and albumin values.

^c^adjusted for patient demographics, comorbid illness, primary conditions at ICU admission and the most abnormal laboratory values during 24 h surrounding ICU admission, categorized to the APACHE III scoring method.

^d^severity-adjusted for APS and APACHE IV scores.

^e^adjusted for severity of illness.

^f^adjusted for age and APACHE II scores.

^g^adjusted for age, sex, admission source, and patient comorbidities.

^h^adjusted for severity of illness.

We contacted 24 authors from 22 different studies regarding study characteristics and missing or unclear data. Four authors provided additional information about unclear data and patient details [24,39,45,48].

### Risk of bias of included studies

The risk of bias of the main outcomes ICU mortality, overall mortality at longest follow-up, ICU LOS, and quality of life were separately assessed in three different cRCTs [31,41,45] by using the RoB 2 tool [10] (S7 Table). Spies 2023 [45] was rated as overall high risk of bias for all outcomes due to recruitment bias leading to selection of sicker patients to the telemedicine group. Additionally, all outcomes, except quality of life, were not adjusted for time trends in this study. ICU mortality in Marx 2022 [31] was assessed as overall high risk of bias due to deviations from intended intervention as sicker patients in the telemedicine group preferably received the intervention, combined with bias for missing adjustment of time trends. ICU LOS overall risk of bias in Pereira 2024 [41] was rated as ‘some concerns’ due to bias in measurement of the outcome, and ICU mortality was rated as ‘no concerns’ (S7 Table).

We assessed the main outcomes ICU mortality, overall mortality at longest follow-up, and ICU LOS in eight different NRSIs [27–29,34,39,44,46,49] using ROBINS-I [12] (S8 Table). ICU mortality was assessed as overall moderate risk of bias in one study [28], serious in three studies [28,44,46], and critical in three studies [29,34,39]. Both studies, contributing overall mortality at longest follow-up, received an overall critical risk of bias [27,34]. The overall risk of bias for ICU LOS was rated as moderate [28], serious [49], and critical [34] in one study each (S8 Table). Problematic studies most frequently revealed bias in selection of participants and bias due to missing data.

### Effects of interventions: primary outcomes

#### Mortality on intensive care units.

**Fig 2:**
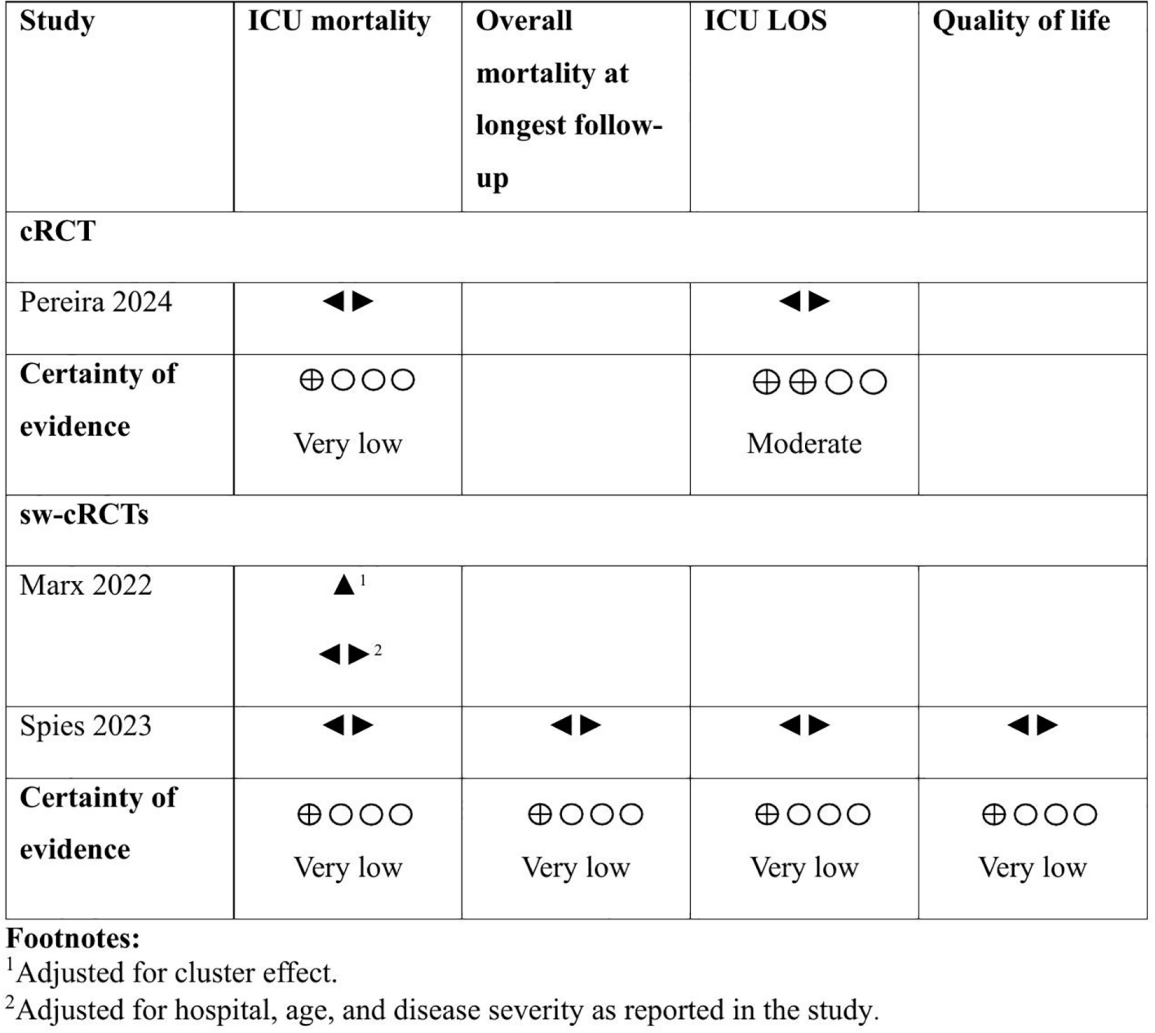
Effect direction plot for RCTs. Effect direction plot summarizing findings by showing the direction of effects across RCTs. **Abbreviations:** Cluster randomized controlled trial (cRCT), intensive care unit (ICU), length of stay (LOS), randomized controlled trial (RCT), standard of care (SoC), stepped-wedge cluster randomized controlled trial (sw-cRCT). **Legend:** ▲ telemedicine increases outcome. ◄► telemedicine has no to minimal effect on outcome.

Two sw-cRCTs involving 5,915 participants reported ICU mortality but were not pooled in a meta-analysis due to clinical and methodological heterogeneity [31,45]. Additionally, one cRCT involving 15,230 participants reported ICU mortality [41]. Spies 2023 (RR 0.89 (95% CI 0.54–1.46)) and Pereira 2024 (RR 1.09 (95% CI 0.94–1.26)) showed no evidence for a difference between telemedicine and SoC, while evidence in Marx 2022 (RR 2.29 (95% CI 1.51 – 3.48)) favoured SoC. In both sw-cRCTs, selection of considerably sicker patients for telemedicine likely biased the observed results. This assumption is supported by disease severity adjusted data for ICU mortality (OR 1.28, 95% CI 0.91–1.79)) showing no evidence for a difference between groups [31]. Certainty of evidence for Marx 2022 and Spies 2023 was very low due to very serious risk of bias and serious inconsistency, while for Pereira 2024 certainty of evidence was very low due to extremely serious imprecision (Table 2, Fig 2).

**Fig 3:**
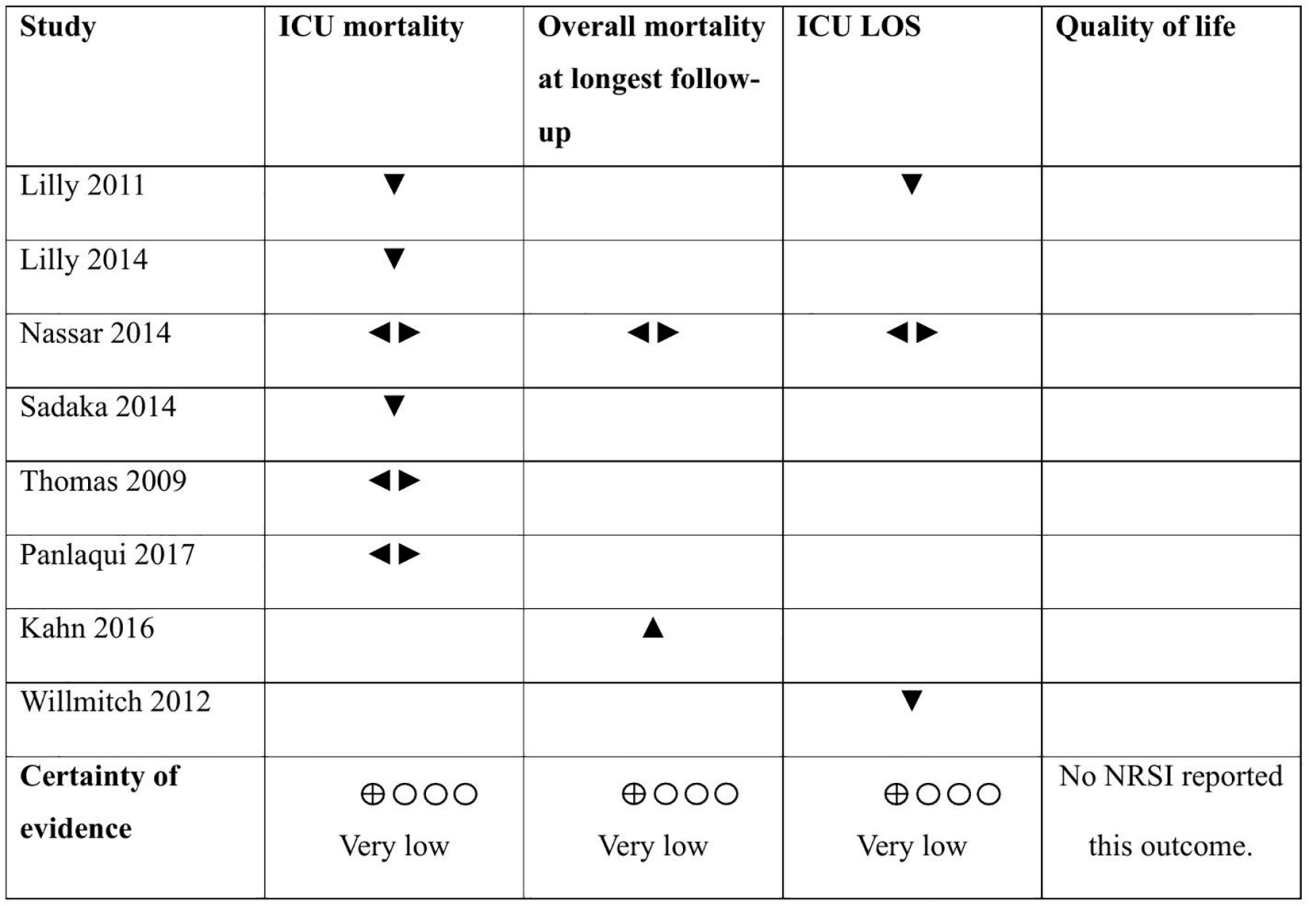
Effect direction plot for NRSIs. Effect direction plot summarizing findings by showing the direction of effects across NRSIs. **Abbreviations:** Intensive care unit (ICU), length of stay (LOS), non-randomized study of intervention (NRSI), standard of care (SoC). **Legend:** ▲ telemedicine increases outcome. ▼ telemedicine decreases outcome. ◄► telemedicine has no to minimal effect on outcome.

Six NRSIs reported adjusted effect estimates for ICU mortality involving 136,125 participants. Studies were not combined in meta-analysis due to clinical and methodological heterogeneity [28,29,34,39,44,46]. Evidence from three studies favoured telemedicine [28,29,44], while the others showed no evidence for a difference between telemedicine and SoC [34,39,46]. Certainty of evidence was very low due to very serious risk of bias and serious inconsistency (Table 3, Fig 3).

### Overall mortality at longest follow-up

One sw-cRCT reported overall mortality at 180 days (n = 1,462), which showed no evidence for a difference between telemedicine and SoC (RR 1.02 (95% CI 0.76–1.36)) [45]. Certainty of evidence was very low due to very serious risk of bias and serious imprecision (Table 2, Fig 2).

Two NRSIs (n = 295,991) reported overall mortality at longest follow-up but were not pooled due to clinical and methodological heterogeneity. One study showed no evidence for a difference between telemedicine and SoC for 30-day mortality [34] and another favoured SoC for 90-day mortality [27]. Certainty of evidence was very low due to very serious risk of bias and serious imprecision (Table 3, Fig 3).

### Length of stay on intensive care units

Length of stay on ICUs was reported by one cRCT (mean (SD) 8.1 (10) days (telemedicine) vs 7.1 (9) days (SoC)) and one sw-cRCT (median [IQR] 6 [4–13] days (telemedicine) vs 5 [3–11] days (SoC)) (n = 16,692) [41,45]. Both studies showed no evidence for a difference between telemedicine and SoC. For Spies 2023, certainty of evidence was very low due to very serious risk of bias and serious imprecision thus the evidence is very uncertain about the effect of telemedicine on ICU LOS. Certainty of evidence for Pereira 2024 was moderate due to serious risk of bias consequently telemedicine probably has little or no difference on ICU LOS (Table 2, Fig 2).

Three NRSIs (n = 21,933) reported adjusted data on ICU LOS but were not pooled due to clinical and methodological heterogeneity [28,34,49]. The studies provided conflicting results: data from two studies favoured telemedicine [28,49], while one study showed no evidence for a difference between telemedicine and SoC [34]. Certainty of evidence was very low due to very serious risk of bias and serious inconsistency (Table 3, Fig 3).

### Quality of life (at 180 day)

Quality of life, measured in one stepped-wedge cluster-RCT (n = 786) using the EQ-5D-5L, showed no evidence for a difference between telemedicne and SoC (MD -2.71 (95% CI 6.95 fewer to 1.53 more)) [45]. Certainty of evidence was very low due to very serious risk of bias and serious imprecision (Table 2, Fig 2).

### Effects of interventions: secondary outcomes

None of the included studies addressed disease-related detection rate, acceptance, changes in therapeutic goals, or triage outcomes. Studies investigating hospital mortality and hospital LOS reported heterogeneous results (S9 Table, S10 Table). Adherence to best practice guidelines was notably higher in telemedicine groups [31] (S11 Table). The same study demonstrated increased compliance with sepsis bundles and timely antibiotic administration in the telemedicine group [31] (S11 Table). Additionally, this sw-cRCT found a slight reduction in antibiotic days with telemedicine [31] (S12 Table). One RCT noted fewer ventilator-free days at 28 days in telemedicine settings [41], while another reported a longer duration of mechanical ventilation for telemedicine patients [45] (S12 Table). Transfer rate reported in two studies [31,38] was higher in telemedicine groups (S13 Table). One sw-cRCT reported improved fulfilment of a subset of process and quality indicators under telemedicine management [45] (S14 Table). However, interpretation of the clinical relevance of this improvement is challenging due to high baseline adherence in some ICU clusters.

### Subgroup analyses

None of the studies compared different types or modes of telemedicine in intensive care settings.

One study reported ICU mortality for sepsis patients with uncertain effect (OR 0.68 (95% CI: 0.23–10.87) [31].

## Discussion

In this systematic review, we included 26 studies with approximately 2,164,508 analysed patients, examining the effect of telemedicine in intensive care on patient- and clinically-relevant outcomes. The results revealed very low certainty of evidence regarding the effect of telemedicine on ICU mortality, ICU LOS, overall mortality at 30, 90, and 180 days, and quality of life at 180 days due to very serious risk of bias and (extremely) serious imprecision or inconsistency. For ICU LOS, moderate certainty of evidence from one cRCT suggested that telemedicine likely results in little to no difference compared to SoC [41]. However, all findings must be interpreted in the context of methodological study limitations and high heterogeneity among studies. The uncertainty in the findings indicates that current clinical studies are insufficient to address the research questions, highlighting the need for further clinical research.

### Methodological limitations and heterogeneity

Several sources of bias and heterogeneity complicate the interpretation of the evidence. Two sw-cRCTs were prone to selection bias, where telemedicine was preferentially applied to sicker ICU patients, and likely distorting patient-relevant outcomes such as mortality, LOS and quality of life. In several NRSIs, it also remained unclear whether all patients in the intervention group consistently received telemedicine, further increasing the risk of bias [29,39,44,46]. Additionally, hospitals involved in these studies varied widely in ICU or hospital settings, staff allocation, and the implementation of telemedicine interventions leading to high clinical heterogeneity and limited comparability.

Moreover, the differences in authorizations of the telemedicine teams in terms of consultation vs decision making and treatment responsibility presumably leads to a high variability in the implementation of telemedical recommendations in clinical practice. Only one included study reported full authority to the telemedicine provider [23], which may have a more significant impact on patient-relevant outcomes than consultation-only models.

The same applies for the availability of telemedicine providers and their telemedicine expertise as well as the access to training programs, which are also crucial factors. High heterogeneity among studies was also found on analytical level. Adjustments for confounders were inconsistent or entirely absent in many NRSIs [19,20,24,25,27,29,32,33,39,40,43,46–48].

Another contributing factor to uncertainty is that many included studies were designed and powered for different primary outcomes than those analyzed in our systematic review [21,23,24,30,31,40,45,48], resulting in suboptimal study designs or analyses for our research questions. As previously noted in another systematic review [50], it often remains unclear whether telemedicine interventions aim to maintain existing standards of care or to actively improve outcomes — further complicating the interpretation of study objectives and findings.

This pronounced heterogeneity across studies underscores the multifaceted nature of telemedicine as a complex intervention, reflecting its variability in design, delivery, and integration into clinical workflows as well as the degree of personal motivation or engagement among providers and recipients. These inconsistencies collectively challenge the ability to pool results effectively and to draw definitive conclusions about telemedicine ‘s impact on patient-relevant outcomes in intensive care.

### Agreements and disagreements with other studies or systematic reviews

These outcome variations are not unique to our systematic review. Other reviews have likewise observed inconsistent effects in primary studies [4–7], largely attributable to different primary study designs (mainly NRSIs) and differing analytical approaches compared with a strong heterogeneity regarding hospital and ICU characteristics, e.g., the technical implementation, staff allocation, and the delegations’ grade. Kalvelage 2021 [7] pooled adjusted and unadjusted outcome data from NRSIs in meta-analyses, while Chen 2018 [4] and Wilcox 2012 [5] relied exclusively on unadjusted outcome data. Mackintosh 2016 included only two NRSIs, both assessed as high risk of bias [51]. Collectively, a plenty of systematic reviews call attention to the high heterogeneity and reporting problems among studies regarding clinical settings and methodology. However, none of the published systematic reviews opted against pooling due to heterogeneous primary studies, as recommended by Cochrane [52]. Meta-analyses of very diverse studies can be misleading and should only be considered when a group of studies is sufficiently homogeneous in terms of participants, interventions, and outcomes to provide a meaningful summary [52]. A strength of our systematic review is that we chose not to pool the studies, given their heterogeneity. This lack of unity among literature highlights the importance of evidence-based strategies for telemedicine implementation in intensive care. To our knowledge, this is the first systematic review including evidence from (sw-)cRCTs providing a higher level of evidence compared to NRSIs.

## Limitations of this review

A limitation of this systematic review is the exclusion of mixed populations. This approach may have reduced the overall number of included studies and patients. We aimed to include clearly defined ICU populations, as, e.g., patients admitted from the ED can differ substantially from ICU patients in terms of baseline characteristics. The language restriction to German and English resulted in the exclusion of three studies; however, given their non-RCT design and small sample sizes (<1,000 patients), they are unlikely to have a significant impact on our findings. Some additional probably relevant outcomes — such as the fulfilment of process and quality indicators — were not analyzed in detail within this review, as their inherent complexity, in combination with the multifaceted nature of telemedicine itself, posed substantial methodological challenges. Additionally, incomplete reporting or lack of response from study authors regarding missing or unclear data further hindered comprehensive analysis.

## Research gaps and future directions

Future research should prioritize well-designed, patient-centered RCTs with defined study populations, best achievable blinding (at least during the process of outcome assessment), transparent reporting, and appropriate outcome selection. The outcomes to be assessed have to be chosen wisely and they should be interpreted carefully with respect to the patients’ need as telemedicine can influence many different aspects of clinical treatment on ICU. For example, an increase in ICU mortality under telemedicine may indeed represent a patient-centered beneficial effect, if the telemedical intervention focused on integration and extension of palliative care in daily ICU routine. In other ICU settings and if adequate, long-term patient-relevant outcomes such as quality of life and overall hospital mortality should be included. Moreover, standardizing outcome measures and adjustment methods are critical for comparability across studies.

Comparative evaluations of different telemedicine models, such as consultation-only systems vs therapeutic approaches, high-tech vs low-tech setups, and varying communication modes (e.g., daily rounding vs on demand), are crucial to determine the most effective models and implementation strategies and should be included in future studies.

Telemedicine theoretically offers promising benefits, such as improved access to specialist care and reduced healthcare disparities, particularly in underserved regions. However, it may also entail potential risks, including reduced human-to-human contact at bedside, which may lower acceptance and trust among families or on-site staff, and transfer of large volumes of sensitive health data, raising concerns about privacy and data security. These potential harms warrant thorough investigation alongside evaluations of clinical effectiveness.

Given the growing demand for ICU services in aging populations and rising healthcare complexities, addressing these research gaps is vital. Telemedicine has the potential to enhance the delivery of optimal intensive care services and improve medical access. It is already widely implemented in clinical practice across the globe and recognized for its high clinical relevance, as highlighted in the forthcoming German AWMF S3-Guideline on telemedicine in intensive care [8]. However, our systematic review cannot yet offer telemedicine users a clear, evidence-based roadmap for optimal implementation, underlining the urgent need for rigorous studies that demonstrate efficacy of telemedicine in intensive care and guide best practices due to the discussed gaps in the existing evidence.

## Conclusion

In conclusion, the current methodological limitations and insufficient reporting of the included studies pose considerable challenges to reliably assess the effects of telemedicine in intensive care on patient-relevant outcomes, reflected in the uncertainty of the evidence. While many different studies indicate possible positive effects of telemedicine on intensive care delivery, robust, patient-centered research is needed to evaluate both clinical effectiveness and implementation strategies across diverse settings with the aim of addressing persistent evidence gaps.

### Patient and public involvement

Patients or the public were not involved in the design, conduct, reporting, or dissemination plans of our research.

## Supporting information

S1 Checklist The PRISMA 2020 statement: an updated guideline for reporting systematic reviews. Page MJ, McKenzie JE, Bossuyt PM, Boutron I, Hoffmann TC, Mulrow CD, et al. The PRISMA 2020 statement: an updated guideline for reporting systematic reviews. BMJ 2021;372:n71. doi: 10.1136/bmj.n71. This work is licensed under CC BY 4.0.
(DOCX)

S1 Protocol PROSPERO, registration number CRD42024547985. (PDF)

S1 Text Searches for primary studies (Randomized Controlled Trials and others). (DOCX)

S1 Table Excluded studies with reasons of the primary search. (DOCX)

S2 Table Ongoing studies and studies held in ‘awaiting classification’ of the primary search. (DOCX)

S3 Table Excluded studies with reasons of update RCT searches. (DOCX)

S4 Table Telemedicine characteristics of included studies. (DOCX)

S5 Table Mean and median age per group of all included studies. (DOCX)

S6 Table Number and percentages of males per group of all included studies. (DOCX)

S7 Table Risk of bias results for (sw-)cRCTs assessed with RoB 2. (DOCX)

S8 Table Risk of bias results for NRSIs assessed with ROBINS-I. (DOCX)

S9 Table Secondary outcome hospital mortality; data from two (sw-)cRCTs and nine NRSIs. (DOCX)

S10 Table Secondary outcome hospital LOS; data from one sw-cRCT and data from seven NRSIs. (DOCX)

S11 Table Secondary outcome adherence to best practice guidelines; data from one sw-cRCT (Marx 2022); adjusted for treating hospital, patient age, and SOFA score. (DOCX)

S12 Table Secondary outcome disease specific effects; data from three (sw-)cRCTs. (DOCX)

S13 Table Secondary outcome transfer rate; data from one sw-cRCT and data from one NRSI. (DOCX)

S14 Table Secondary outcome fulfilment of process and quality indicators; data from one sw-cRCT; adjusted for time, with random intercepts for patient, and centre levels. (DOCX)
