# Supplementary material for: Telemedicine in adult intensive care: A systematic review of patient-relevant outcomes and methodological considerations
Source: PLOS Digit Health. 2025 Dec 15;4(12):e0001126. doi: 10.1371/journal.pdig.0001126 (PMC12704867; doi:10.1371/journal.pdig.0001126)
Supplement: S1 Protocol — (PDF) [file pdig.0001126.s002.pdf]

Impact of telemedicine in ICU care on patient-relevant and process-relevant outcomes: a systematic review protocol

Tamara Pscheidl, Lena Saal-Bauernschubert, Karolina Dahms, Eva Steinfeld, Julia Dormann, Kelly Ansems, Sandra Dohmen, Carina Benstoem, Anne Ritter, Miriam Stegemann, Claire Iannizzi, Ana-Mihaela Zorger, Nicole Skoetz, Heidrun Janka, Maria-Inti Metzendorf, Carla Nau, Falk von Dincklage, Stephanie Weibel

1. Administrative information

1	Review title	Impact of telemedicine in ICU care on patient-relevant and process-relevant outcomes: a systematic review protocol
2	Original language title	N.A.
3	Anticipated or actual start date	18.04.2024
4	Anticipated completion date	October 2024
5	Stage of review at time of submission	Data extraction
6	Named contact	Stephanie Weibel
7	Named contact email	Weibel_S@ukw.de
8	Named contact address	University Hospital Würzburg<br>Department of Anaesthesiology, Intensive Care, Emergency and Pain Medicine<br>Oberdürrbacher Str. 6<br>97080 Würzburg<br>Germany
9	Named contact phone number	+49-931-201-30310
10	Organisational affiliation of the review	University Hospital Würzburg
11	Review team members and their organisational affiliations	Tamara Pscheidl, University Hospital Würzburg Department of Anaesthesiology, Intensive Care, Emergency and Pain Medicine, Germany<br>Lena Saal-Bauernschubert, University Hospital Würzburg Department of Anaesthesiology, Intensive Care, Emergency and Pain Medicine, Germany<br>Karolina Dahms, Department of Intensive Care Medicine and Intermediate Care, Medical Faculty, RWTH Aachen University, Aachen, Germany

		Eva Steinfeld, Department of Intensive Care Medicine and Intermediate Care, Medical Faculty, RWTH Aachen University, Aachen, Germany<br>Julia Dormann, Department of Intensive Care Medicine and Intermediate Care, Medical Faculty, RWTH Aachen University, Aachen, Germany<br>Kelly Ansems, Department of Intensive Care Medicine and Intermediate Care, Medical Faculty, RWTH Aachen University, Aachen, Germany<br>Sandra Dohmen, Department of Intensive Care Medicine and Intermediate Care, Medical Faculty, RWTH Aachen University, Aachen, Germany<br>Carina Benstoem, Department of Intensive Care Medicine and Intermediate Care, Medical Faculty, RWTH Aachen University, Aachen, Germany<br>Anne Ritter, Charité-Universitätsmedizin Berlin, corporate member of Freie Universität Berlin and Humboldt-Universität zu Berlin, Department of Infectious Diseases, Respiratory Medicine and Critical Care<br>Miriam Stegemann, Charité-Universitätsmedizin Berlin, corporate member of Freie Universität Berlin and Humboldt-Universität zu Berlin, Department of Infectious Diseases, Respiratory Medicine and Critical Care<br>Claire Iannizzi, University of Cologne, Institute of Public Health, Germany<br>Ana-Mihaela Zorger, University of Cologne, Institute of Public Health, Germany<br>Nicole Skoetz, University of Cologne, Institute of Public Health, Germany<br>Heidrun Janka, Institute of General Practice, Medical Faculty of the Heinrich-Heine-University Düsseldorf, Düsseldorf, Germany<br>Maria-Inti Metzendorf, Institute of General Practice, Medical Faculty of the Heinrich-Heine-University Düsseldorf, Düsseldorf, Germany<br>Carla Nau, University Medical Center Schleswig-Holstein, Campus Lübeck, Department of Anaesthesiology and Intensive Care Medicine, Lübeck, Germany<br>Falk von Dincklage, Universitätsmedizin Greifswald Körperschaft des öffentlichen Rechts, Klinik für Anästhesie, Intensiv-, Notfall- und Schmerzmedizin, Greifswald, Germany
12	Funding sources	This systematic review is part of the project UTN which is funded by ‘Bundesministerium für Bildung und Forschung (BMBF)’ as part of the ‘Netzwerk Universitätsmedizin 2.0’ (NUM 2.0), No. 01KX2121
13	Conflicts of interest	TP, LSB, KD, ES, JD, KA, SD, CB, AR, MSt, CI, AMZ, NS, HJ, MIM, CN, FvD SW: none
14	Collaborators	UTN partners (Björn Weiß, Berlin; Maria Deja, Lübeck; Sven Laudi, Leipzig; Falk Fichtner, Leipzig)

Content 15 - 30 below		
31	Language	English
32	Country	Germany
33	Other registration details	
34	Reference and/or URL for published protocol	
35	Dissemination plan	Journal publication, evidence syntheses for the German AWMF-guideline 'S3-Leitlinie Telemedizin in der Intensivmedizin', AWMF-Registernummer: 001-034 (additional publication in English is planned)
36	Keywords	Tele-ICU, Telemedicine, ICU
37	Details of any existing review of the same topic by the same authors	Decision-Making Authority During Tele-ICU Care Reduces Mortality and Length of Stay-A Systematic Review and Meta-Analysis. Christina Kalvelage, Susanne Rademacher, Sandra Dohmen, Gernot Marx, Carina Benstoem. Crit Care Med. 2021 Jul 1;49(7):1169-1181 DOI: 10.1097/CCM.0000000000004943
38	Current review status	ongoing
39	Any additional information	
40	Details of final report / publication or preprints if available	

15. Review question

To assess patient-relevant and process-relevant outcomes in any critically ill patient comparing telemedicine (TM) to standard of care (SoC) or to any other type or mode of TM in intensive care settings. We will explore interventional (see "intervention") and disease-related aspects in subgroups (see „participants/population") that benefit from TM.

16. Searches

We will search the following bibliographic databases from inception until 18 April 2024:

- MEDLINE (via Ovid)
- Scopus
- CINAHL
- Cochrane Central Register of Controlled Trials (CENTRAL)

In addition, we will search the following trial registries to identify completed, unpublished and ongoing studies:

- Clinicaltrials.gov

- WHO International Clinical Trials Registry Platform (ICTRP)

We will consult the following additional resources:
- Screening references of all included primary studies and identified systematic review articles

17. URL to search strategy

Link to search strategy for MEDLINE in pdf:
https://www.crd.york.ac.uk/PROSPEROFILES/66c455811a47a4d25a28fe02d80fcdf9.pdf.

18. Condition or domain being studied

Telemedicine-Intensive-Care-Units (tele-ICUs) provide continuous, connected and/or partially algorithmically controlled care to augment local intensive care unit (ICU) teams. Tele-ICU experts can work as single experts or in interdisciplinary or multiprofessional clinical teams using a variety of different audio, audio-visual, and data transfer technologies to provide optimal evidence-based care to every patient admitted to an ICU, regardless of time or location. However, a number of questions remain regarding the type and mode in which tele-ICU should be ideally implemented and performed, as well as the question which diseases and/or situations in the ICU setting benefit most from tele-intensive-care consultation or therapy.

19. Participants/population

Inclusion criteria: studies investigating

- Any critically ill adult (≥ 18 years) inpatient on any ICU or critical care unit (CCU), irrespective of sex, ethnicity, comorbidities, ICU specialization, disease or clinical condition, including relevant subgroups regarding the clinical disease, i.e.:
  - Sepsis
  - Acute Respiratory Distress Syndrome (ARDS)
  - COVID-19
  - Other (non-COVID-19) infectious diseases
  - One organ dysfunction (e.g. cardiogenic shock, liver failure, kidney failure)
  - Multiple organ dysfunctions
  - Polytrauma / multiple injury
  - Traumatic brain injury
  - Haematological / oncological patients / stem cell therapy
- Situation(s) or condition(s), i.e.:
  - Prolonged mechanical ventilation
  - Prolonged weaning from the respirator
  - Prior to Extracorporeal Membrane Oxygenation (ECMO) implantation
  - Transfer request
  - End of life decisions / organ donation / brain death diagnosis
  - Limited resources (i.e. lack of personnel, technical capacity, expertise) / triage questions

Exclusion criteria: studies on

- Children < 18 years
- Not ICU/CCU-patients (settings)

- Patients in an emergency department

In case we identify studies including mixed populations, we will include these only if subgroup data on our eligible population are reported separately.

20. Intervention(s) / exposure(s)

- TM is defined for this review as a standardized
  - Audio- OR
  - Audio- and video-connection

  by using high-tech (e.g. remote-controlled cameras or robots specifically designed for TM) or low-tech (e.g. laptops, mobile phones) equipment, and optionally in combination with shared electronic health records (EHR) with automated data transfer (e.g. real-time access to patient records including vital signs, ventilator settings, patient files with laboratory parameters, decision-support tools, note sharing, alerts, and imaging, such as radiography and echocardiography) or without automated data transfer (e.g. screen sharing, email, fax).
- Tele-Intensive-Care is defined as any TM from ICU-professionals (e.g. one intensivist, multiprofessional/interdisciplinary team, or critical care nurse) located in a health-care institution or tele-center to ICU-professionals (e.g. one intensivist, multiprofessional team, physician, or nurse) in another health-care institution with ICU-setting located elsewhere (i.e. not in-house), e.g. from maximum healthcare provider to non-maximum healthcare provider.
- Tele-Intensive-Care is defined as an assessment of all organ systems.

If unclear what type of TM was used (e.g. audio, audio-video with or without automated data transfer) the study will be classified as "awaiting classification" and we will contact the study authors for further information.

21. Comparator(s) / control

- SoC in an ICU-setting defined as care without TM
- Any other type or mode of TM

Eligible comparisons for this review will be:

- TM vs SoC
- Any type of TM vs any other type of TM (see data extraction for types of TM)
- Any mode of TM vs any other mode of TM (see data extraction for modes of TM)

22. Types of studies to be included

We aim to include primary studies of the following study designs:

- Any controlled studies:
  - Randomized controlled trials (RCTs) (including non-standard RCT designs, such as cluster-randomized trials)
  - Prospective and retrospective non-randomized studies of interventions (NRSIs), including quasi-randomized controlled trials, controlled before-and-after (CBA) studies, cohort studies, case-control studies

We consider the following publication formats:

- Peer-reviewed journal publications
- Preprint articles
- Results published in trial registries

- Completed, but unpublished studies in trial registers and ongoing-studies

We will restrict our search to reports in English or German published since 1999, and including ten or more participants.

We will not consider the following study designs: qualitative studies or uncontrolled studies, and systematic reviews or meta-analyses.
We will not consider the following publication formats: conference abstracts and posters.

23. Context

Studies conducted in hospital environment, specifically ICU-setting

24. Main outcomes

Patient relevant outcomes:

- Mortality/Lethality in ICU
- Mortality/Lethality in hospital
- Overall mortality
- Length of stay (LOS) ICU
- LOS Hospital
- Patient/unit of care centered outcomes e.g. functional outcomes, quality of life
- Disease related detection rate (e.g. correctly diagnosed)
- Disease-specific effects (e.g. adequate antibiotic therapy, antibiotic consumption, ventilation, positioning)
- Transfer rate (i.e. from TM recipient to other clinics, e.g. TM provider)
- Acceptance (e.g. patient, family, care givers)

Process and quality indicators:

- Adherence to best practice guidelines (e.g. sepsis management, lung protective ventilation)
- Fulfilment of process and quality indicators (e.g. start of enteral nutrition, start of antibiotic treatment, daily interdisciplinary visits)
- Change of therapeutic goal
- Triage result

We defined four outcomes from the list above as our main outcome set:

- Mortality/Lethality in ICU
- Overall mortality at longest follow up
- LOS ICU
- Patient/unit of care centered outcome: quality of life at longest follow up

Measures of effect

Dichotomous outcomes: risk ratio (RR) with a 95% confidence intervals (CI)
Continuous outcomes: mean difference (MD) with 95% CIs if using the same scale. Standardized mean difference (SMD) with 95% CI if using different scales.

Time-to-event (TTE) outcomes: Hazard ratios (HRs) and 95% CIs. If not directly provided, we will attempt to recalculate TTE outcomes based on methods proposed by Tierney 2007.

25. Additional outcomes

none (see above)

Measures of effect

See above.

26. Data extraction (selection and coding)

Study selection: Two reviewers will perform study selection independently according to predefined eligibility criteria in accordance with the Cochrane Handbook of Intervention studies in a two-step approach. First, two authors will screen titles and abstracts of identified records. Disagreements between individual judgments will be resolved by discussion, and in case of doubt, the study will be carried over to the full-text screening stage. We will then retrieve full text-articles of all doubtful and potentially eligible records and assess eligibility of these remaining records (in duplicate). Disagreements between two authors will be solved by discussion, and if needed, by consulting a third review author.

When more than one article presents data on the same population, we will include the primary article, which will be the article with the largest number of people or with the most informative data.

We will use *Covidence* for screening.

Data extraction: Two review authors will independently extract data using a piloted data extraction form, including details on

- General study information
    - Author, year, journal, language, type of publication, source of publication, study objective, study setting, country of study conduct, funding source, trial registration number, conflict of interest
- Study characteristics
    - Type of study design, time point of data collection
- Participants
    - Age, sex, number of participants allocated, treated, and analyzed, number of participants in ICU setting, disease/condition of ICU participants, situation/condition of the ICU participants
- Intervention
    - Type of ICU specialization (provider/recipient), clinical setting, type of hospital (provider/recipient)
    - Technical implementation: type of communication (audio or audio-video with or without EHR with or without automated data transfer), mode of communication (emergency contact, contact on demand, unstructured or structured contact on demand, daily rounding, unstructured/structured daily rounding)
    - Executing person and delegation: delegation's grade, staff allocation for tele-intensive-care (provider/recipient), expertise/experience in tele-intensive-care (provider/recipient)
- Comparator
    - SoC or other type or mode of TM

- Technical architecture: provider/recipient (high-tech, low-tech)
- Technical implementation: type of communication, mode of communication
- Executing person and delegation: delegations grade, staff allocation for tele-ICU (provider/recipient), expertise/experience in tele-intensive-care (provider/recipient)

- Outcomes
  - Patient-relevant outcomes: see above
  - Quality- and process indicators: see above

If necessary, we will try to receive missing data by contacting the authors of relevant publications. At each step of data extraction, we will resolve discrepancies by discussion within the group of review authors.

The data will be extracted using Covidence.

27. Risk of Bias (quality) assessment

Two review authors will independently assess the risk of bias for RCTs using the Cochrane Risk of bias tool 2 (RoB 2) for each study and each relevant study outcome and a variant of the RoB 2 tool specifically for cluster-randomized trials, and for NRSIs using the ROBINS-I tool. The review authors will resolve disagreements by discussion with a third review author.

Risk of bias will be assessed for outcomes of the main outcome set which contribute to the review's 'Summary of findings' table.

28. Strategy for data synthesis

If clinical and methodological characteristics of individual identified studies are sufficiently homogeneous, we will pool data in meta-analyses. We will not pool data from RCTs and NRSIs. We will use the direct estimate of cluster-randomized trial if the required effect measure (e.g. an risk ratio with its confidence interval) comes from an analysis which properly accounts for the cluster design. Effect estimates and their standard errors from correct analyses of cluster-randomized trials will be meta-analysed using the generic inverse-variance approach. When the study authors have not conducted such an analysis, we will use an approximate approach to adjust the results as described in the Cochrane Handbook (https://training.cochrane.org/handbook/current/chapter-23#section-23-1-4).
We will use the random-effects model as we assume that the intervention effects will be related but will not be the same for included studies. We will analyze the following comparisons separately:

- Any TM vs. SoC/no telemedicine
- Any type of TM vs. any other type of TM
- Any mode of TM vs any other mode of TM

Time-to-event outcomes: we will record hazard ratios and 95%-CIs from published data. For the eventuality that HRs are not available, we will make every effort to estimate the HR as accurately as possible from other available data, i.e., Kaplan-Meier curves.

Continuous outcomes: we will record mean, standard deviation, and number of participants per group. We will calculate mean differences with 95%-CIs, in case trials referring to one scale, and standardized

mean differences, in case trials referring to different scales. We will perform analyses using the inverse variance method under a random-effects model.

Binary outcomes: we will record the number of affected participants and the number of participants per group. We will perform analyses using the Mantel-Haenszel method under a random-effects model to report pooled risk ratios with 95%-CIs.

We will use R for analysis.

We will measure statistical heterogeneity using the χ^2 test, the I^2 statistic, and the 95% prediction interval (PI) for random-effects meta-analysis. We will restrict the calculation of a 95% PI to meta-analyses with ≥ 4 studies and ≥ 200 participants, since the interval would be imprecise when a summary estimate was based on only a few small studies. We will use the R package meta to calculate 95% PIs. We will declare statistical heterogeneity if $P < 0.05$ for the χ^2 statistic, or $I^2 \geq 40\%$ (40% - 60%: moderate heterogeneity; 50% - 90%: substantial heterogeneity; 75% - 100%: considerable heterogeneity), or the range of the 95%PI revealed a different clinical interpretation of the effect estimate compared to the 95%CI. We will explore heterogeneity by subgroup analysis).

We will use the GRADE approach to assess certainty in the evidence for outcomes of the main outcome set.

29. Analysis of subgroups or subsets

We plan to perform subgroup analyses (independent of heterogeneity) for the main/primary outcomes to calculate RR, HR, MD, or SMD in conjunction with the corresponding CI for each of the following characteristics if data is available:

Specific characteristics of participants, i.e.:

- Disease-related conditions of the participants (see #19)
- Situation-related conditions of the participants (see #19)

Specific intervention details for the comparison TM vs SoC, i.e.

Type of TM:

- TM using audio-video vs TM using audio only
    - TM with remote-controlled camera in the patient's room vs TM with only audio-video connection
    - TM with consistent EHR with data transfer vs TM with only audio-video connection
    - Use of information and communication technology for telemedical purpose (a complete architecture or parts of it) in a hospital vs only audio-video connection

Mode of TM:

- TM with quality indicator based expert interview / structured indicator based expert interview vs TM with unstructured or not defined expert interview
- TM in daily rounding vs TM on demand
- Any TM given by a tele-ICU expert (e.g. training in TM or practical experience in TM) vs same TM given by any other ICU personal

- Any TM taken by any ICU personal with experience in TM (e.g. training in TM or practical experience in TM) vs same TM taken by any other ICU personal
- Any TM consultation by multiprofessionals vs interdisciplinary teams vs intensivists alone

Sensitivity analysis:

- Risk of bias (analysis including only studies at low and unclear risk of bias compared with the analysis including all studies of this comparison and outcome)

- RCTs only (without cluster-randomized trials)

- If only cluster-randomized trials are available, estimates from properly conducted studies vs adjusted effect estimates will be compared
- Fixed-effects model meta-analysis.

30. Type and method of review

Type of review, choose from the following:

- Intervention
- Meta-analysis

Health area of the review, choose from the following:

- Cancer
- Cardiovascular
- Care of the elderly
- COVID-19
- Infections and infestations
- Nursing
- Perioperative care
- Surgery
