# Supplementary material for: Telemedicine in adult intensive care: A systematic review of patient-relevant outcomes and methodological considerations
Source: PLOS Digit Health. 2025 Dec 15;4(12):e0001126. doi: 10.1371/journal.pdig.0001126 (PMC12704867; doi:10.1371/journal.pdig.0001126)
Supplement: S1 Text — (DOCX) [file pdig.0001126.s003.docx]

**Searches for primary studies (Randomized Controlled Trials and others)**

1. Search for primary studies (RCTs and others), comprising:
1a: Telemedicine/ICU; Search date: 09/01/2024
1b: Telemedicine/Acute diseases; Search date: 18/04/2024
1c: 1a + 1b + RCT-Filter; search limited to MEDLINE and CENTRAL; Search date: 26/10/2024

1d: 1a + 1b + RCT-Filter; search limited to MEDLINE and CENTRAL; Search date: 29/09/2025

**1a: Telemedicine/ICU**

Search date: 09/01/2024

**Ovid MEDLINE(R) ALL**

1. telemedicine/ or telepathology/ or teleradiology/ or telerehabilitation/ or telemetry/

2. (telemedicine or tele-medicine or telemonitoring or tele-monitoring or telehealth or tele-health or telecare or tele-care or teleradiology or tele-radiology or telepathology or tele-pathology or telerehabilitation or tele-rehabilitation or telereferral or tele-referral or digital health or digital intervention* or remote intervention* or remote care or remote consultation* or remote counceling or remote monitoring or remote nursing or remote presence or remote screening or remote therapy or mobile health or mhealth or m-health or electronic health or ehealth or e-health or e-therapy or etherapy or electronic counceling or electronic monitoring or electronic intervention* or robotic telepresence or distance counceling or distance monitoring or virtual monitoring or virtual medicine or virtual care or virtual nursing or virtual telepresence or internet-based intervention* or internet intervention* or online intervention* or online-based intervention* or web-based intervention* or web intervention* or electronic consultation* or cyberconsultation* or e-consultation* or econsultation* or internet consultation* or internet-based consultation* or online consultation* or web consultation* or web-based consultation* or webbased consultation* or long distance consultation* or long-distance health care* or off-site health care* or off-site care).tw.

3. 1 or 2

4. intensive care units/ or burn units/ or coronary care units/ or recovery room/ or respiratory care units/

5. (intensive care or icu or icus or respiratory care unit* or coronary care unit* or burn unit* or acute trauma care or trauma cent* or trauma unit* or trauma resuscitation unit* or acute care or critical care or critically ill or critical illness or acutely ill or acute illness).tw.

6. 4 or 5

7. 3 and 6

8. (tele-ICU or tele-ICUs or teleICU or teleICUs or e-ICU or e-ICUs or eICU or eICUs or tele-intensive care or remote ICU or remote ICUs or electronic ICU or electronic ICUs or electronic intensive care or digital ICU or digital ICUs or digital intensive care or remote intensive care).tw.

9. 7 or 8

= 4660

**CENTRAL via Cochrane Library**

#1 MeSH descriptor: [Telemedicine] this term only

#2 MeSH descriptor: [Telepathology] this term only

#3 MeSH descriptor: [Teleradiology] this term only

#4 MeSH descriptor: [Telerehabilitation] this term only

#5 (telemedicine or tele-medicine or telemonitoring or tele-monitoring or telehealth or tele-health or telecare or tele-care or teleradiology or tele-radiology or telepathology or tele-pathology or telerehabilitation or tele-rehabilitation or telereferral or tele-referral or (digital NEXT health) or (digital NEXT intervention*) or (remote NEXT intervention*) or (remote NEXT care) or (remote NEXT consultation*) or (remote NEXT counceling) or (remote NEXT monitoring) or (remote NEXT nursing) or (remote NEXT presence) or (remote NEXT screening) or (remote NEXT therapy) or (mobile NEXT health) or mhealth or m-health or (electronic NEXT health) or ehealth or e-health or e-therapy or etherapy or (electronic NEXT counceling) or (electronic NEXT monitoring) or (electronic NEXT intervention*) or (robotic NEXT telepresence) or (distance NEXT counceling) or (distance NEXT monitoring) or (virtual NEXT monitoring) or (virtual NEXT medicine) or (virtual NEXT care) or (virtual NEXT nursing) or (virtual NEXT telepresence) or (internet-based NEXT intervention*) or (internet NEXT intervention*) or (online NEXT intervention*) or (online-based NEXT intervention*) or (web-based NEXT intervention*) or (web NEXT intervention*) or (electronic NEXT consultation*) or cyberconsultation* or e-consultation* or econsultation* or (internet NEXT consultation*) or (internet-based NEXT consultation*) or (online NEXT consultation*) or (web NEXT consultation*) or (web-based NEXT consultation*) or (webbased NEXT consultation*) or (long NEXT distance NEXT consultation*) or (long-distance NEXT health NEXT care*) or (off-site NEXT health NEXT care*) or (off-site NEXT care)):ti,ab,kw

#6 #1 or #2 or #3 or #4 or #5

#7 MeSH descriptor: [Intensive Care Units] this term only

#8 MeSH descriptor: [Burn Units] this term only

#9 MeSH descriptor: [Coronary Care Units] this term only

#10 MeSH descriptor: [Recovery Room] this term only

#11 ((intensive NEXT care) or icu or icus or (respiratory NEXT care NEXT unit*) or (coronary NEXT care NEXT unit*) or (burn NEXT unit*) or (acute NEXT trauma NEXT care) or (trauma NEXT cent*) or (trauma NEXT unit*) or (trauma NEXT resuscitation NEXT unit*) or (acute NEXT care) or (critical NEXT care) or (critically NEXT ill) or (critical NEXT illness) or (acutely NEXT ill) or (acute NEXT illness)):ti,ab,kw

#12 #7 or #8 or #9 or #10 or #11

#13 #6 and #12

#14 (tele-ICU or tele-ICUs or teleICU or teleICUs or e-ICU or e-ICUs or eICU or eICUs or (tele-intensive NEXT care) or (remote NEXT ICU) or (remote NEXT ICUs) or (electronic NEXT ICU) or (electronic NEXT ICUs) or (electronic NEXT intensive NEXT care) or (digital NEXT ICU) or (digital NEXT ICUs) or (digital NEXT intensive NEXT care) or (remote NEXT intensive NEXT care)):ti,ab,kw

#15 #13 or #14

#16 #15 in Trials

= 656

**CINAHL**

#1 (MH "Telemedicine+") OR (MH "Telenursing")

#2 TI ( telemedicine or tele-medicine or telemonitoring or tele-monitoring or telehealth or tele-health or telecare or tele-care or teleradiology or tele-radiology or telepathology or tele-pathology or telerehabilitation or tele-rehabilitation or telereferral or tele-referral or "digital health" or "digital intervention*" or "remote intervention*" or "remote care" or "remote consultation*" or "remote counceling" or "remote monitoring" or "remote nursing" or "remote presence" or "remote screening" or "remote therapy" or "mobile health" or mhealth or m-health or "electronic health" or ehealth or e-health or e-therapy or etherapy or "electronic counceling" or "electronic monitoring" or "electronic intervention*" or "robotic telepresence" or "distance counceling" or "distance monitoring" or "virtual monitoring" or "virtual medicine" or "virtual care" or "virtual nursing" or "virtual telepresence" or "internet-based intervention*" or "internet intervention*" or "online intervention*" or "online-based intervention*" or "web-based intervention*" or "web intervention*" or "electronic consultation*" or cyberconsultation* or e-consultation* or econsultation* or "internet consultation*" or "internet-based consultation*" or "online consultation*" or "web consultation*" or "web-based consultation*" or "webbased consultation*" or "long distance consultation*" or "long-distance health care*" or "off-site health care*" or "off-site care" ) OR AB ( telemedicine or tele-medicine or telemonitoring or tele-monitoring or telehealth or tele-health or telecare or tele-care or teleradiology or tele-radiology or telepathology or tele-pathology or telerehabilitation or tele-rehabilitation or telereferral or tele-referral or "digital health" or "digital intervention*" or "remote intervention*" or "remote care" or "remote consultation*" or "remote counceling" or "remote monitoring" or "remote nursing" or "remote presence" or "remote screening" or "remote therapy" or "mobile health" or mhealth or m-health or "electronic health" or ehealth or e-health or e-therapy or etherapy or "electronic counceling" or "electronic monitoring" or "electronic intervention*" or "robotic telepresence" or "distance counceling" or "distance monitoring" or "virtual monitoring" or "virtual medicine" or "virtual care" or "virtual nursing" or "virtual telepresence" or "internet-based intervention*" or "internet intervention*" or "online intervention*" or "online-based intervention*" or "web-based intervention*" or "web intervention*" or "electronic consultation*" or cyberconsultation* or e-consultation* or econsultation* or "internet consultation*" or "internet-based consultation*" or "online consultation*" or "web consultation*" or "web-based consultation*" or "webbased consultation*" or "long distance consultation*" or "long-distance health care*" or "off-site health care*" or "off-site care" )

#3 #1 OR #2

#4 (MH "Intensive Care Units") OR (MH "Coronary Care Units") OR (MH "Respiratory Care Units") OR (MH "Stroke Units")

#5 TI ( "intensive care" or icu or icus or "respiratory care unit*" or "coronary care unit*" or "burn unit*" or "acute trauma care" or "trauma cent*" or "trauma unit*" or "trauma resuscitation unit*" or "acute care" or "critical care" or "critically ill" or "critical illness" or "acutely ill" or "acute illness" ) OR AB ( "intensive care" or icu or icus or "respiratory care unit*" or "coronary care unit*" or "burn unit*" or "acute trauma care" or "trauma cent*" or "trauma unit*" or "trauma resuscitation unit*" or "acute care" or "critical care" or "critically ill" or "critical illness" or "acutely ill" or "acute illness" )

#6 #4 OR #5

#7 #3 AND #6

#8 TI ( tele-icu OR tele-icus OR teleicu OR teleicus OR e-icu OR e-icus OR eicu OR eicus OR "tele-intensive care" OR "remote ICU" OR "remote ICUs" OR "electronic ICU" OR "electronic ICUs" OR "electronic intensive care" OR "digital ICU" OR "digital ICUs" OR "digital intensive care" OR "remote care" ) OR AB ( tele-icu OR tele-icus OR teleicu OR teleicus OR e-icu OR e-icus OR eicu OR eicus OR "tele-intensive care" OR "remote ICU" OR "remote ICUs" OR "electronic ICU" OR "electronic ICUs" OR "electronic intensive care" OR "digital ICU" OR "digital ICUs" OR "digital intensive care" OR "remote care" )

#9 #7 OR #8

= 2'632

**Scopus**

(TITLE-ABS ( telemedicine OR tele-medicine OR telemonitoring OR tele-monitoring OR telehealth OR tele-health OR telecare OR tele-care OR teleradiology OR tele-radiology OR telepathology OR tele-pathology OR telerehabilitation OR tele-rehabilitation OR telereferral OR tele-referral OR "digital health" OR "digital intervention*" OR "remote intervention*" OR "remote care" OR "remote consultation*" OR "remote counceling" OR "remote monitoring" OR "remote nursing" OR "remote presence" OR "remote screening" OR "remote therapy" OR "mobile health" OR mhealth OR m-health OR "electronic health" OR ehealth OR e-health OR e-therapy OR etherapy OR "electronic counceling" OR "electronic monitoring" OR "electronic intervention*" OR "robotic telepresence" OR "distance counceling" OR "distance monitoring" OR "virtual monitoring" OR "virtual medicine" OR "virtual care" OR "virtual nursing" OR "virtual telepresence" OR "internet-based intervention*" OR "internet intervention*" OR "online intervention*" OR "online-based intervention*" OR "web-based intervention*" OR "web intervention*" OR "electronic consultation*" OR cyberconsultation* OR e-consultation* OR econsultation* OR "internet consultation*" OR "internet-based consultation*" OR "online consultation*" OR "web consultation*" OR "web-based consultation*" OR "webbased consultation*" OR "long distance consultation*" OR "long-distance health care*" OR "off-site health care*" OR "off-site care" ) AND TITLE-ABS ( "intensive care" OR icu OR icus OR "respiratory care unit*" OR "coronary care unit*" OR "burn unit*" OR "acute trauma care" OR "trauma cent*" OR "trauma unit*" OR "trauma resuscitation unit*" OR "acute care" OR "critical care" OR "critically ill" OR "critical illness" OR "acutely ill" OR "acute illness" )) OR TITLE-ABS ( tele-icu OR tele-icus OR teleicu OR teleicus OR e-icu OR e-icus OR eicu OR eicus OR "tele-intensive care" OR "remote ICU" OR "remote ICUs" OR "electronic ICU" OR "electronic ICUs" OR "electronic intensive care" OR "digital ICU" OR "digital ICUs" OR "digital intensive care" OR "remote care" )

= 4'815

**WHO ICTRP Database**

(tele-icu OR tele-icus OR teleicu OR teleicus OR e-icu OR e-icus OR eicu OR eicus OR "tele-intensive care" OR "remote ICU" OR "remote ICUs" OR "electronic ICU" OR "electronic ICUs" OR "electronic intensive care" OR "digital ICU" OR "digital ICUs" OR "digital intensive care" OR "remote care") OR ((telemedicine OR tele-medicine OR telemonitoring OR tele-monitoring OR telehealth OR tele-health OR telecare OR tele-care OR teleradiology OR tele-radiology OR telepathology OR tele-pathology OR telerehabilitation OR tele-rehabilitation OR telereferral OR tele-referral OR "digital health" OR "digital intervention" OR "remote intervention" OR "remote care" OR "remote consultation" OR "remote counceling" OR "remote monitoring" OR "remote nursing" OR "remote presence" OR "remote screening" OR "remote therapy" OR "mobile health" OR mhealth OR m-health OR "electronic health" OR ehealth OR e-health OR e-therapy OR etherapy OR "electronic counceling" OR "electronic monitoring" OR "electronic intervention" OR "robotic telepresence" OR "distance counceling" OR "distance monitoring" OR "virtual monitoring" OR "virtual medicine" OR "virtual care" OR "virtual nursing" OR "virtual telepresence" OR "internet-based intervention" OR "internet intervention" OR "online intervention" OR "online-based intervention" OR "web-based intervention" OR "web intervention" OR "electronic consultation" OR cyberconsultation OR e-consultation OR econsultation OR "internet consultation" OR "internet-based consultation" OR "online consultation" OR "web consultation" OR "web-based consultation" OR "webbased consultation" OR "long distance consultation" OR "long-distance health care" OR "off-site health care" OR "off-site care") AND ("intensive care" OR icu OR icus OR "respiratory care unit" OR "coronary care unit" OR "burn unit" OR "acute trauma care" OR "trauma center" OR "trauma centre" OR "trauma unit" OR "trauma resuscitation unit" OR "acute care" OR "critical care" OR "critically ill" OR "critical illness" OR "acutely ill" OR "acute illness"))
= 87

**ClinicalTrials.gov**

ALL ((tele-icu OR tele-icus OR teleicu OR teleicus OR e-icu OR e-icus OR eicu OR eicus OR "tele-intensive care" OR "remote ICU" OR "remote ICUs" OR "electronic ICU" OR "electronic ICUs" OR "electronic intensive care" OR "digital ICU" OR "digital ICUs" OR "digital intensive care" OR "remote care") OR ((telemedicine OR tele-medicine OR telemonitoring OR tele-monitoring OR telehealth OR tele-health OR telecare OR tele-care OR teleradiology OR tele-radiology OR telepathology OR tele-pathology OR telerehabilitation OR tele-rehabilitation OR telereferral OR tele-referral OR "digital health" OR "digital intervention" OR "remote intervention" OR "remote care" OR "remote consultation" OR "remote counceling" OR "remote monitoring" OR "remote nursing" OR "remote presence" OR "remote screening" OR "remote therapy" OR "mobile health" OR mhealth OR m-health OR "electronic health" OR ehealth OR e-health OR e-therapy OR etherapy OR "electronic counceling" OR "electronic monitoring" OR "electronic intervention" OR "robotic telepresence" OR "distance counceling" OR "distance monitoring" OR "virtual monitoring" OR "virtual medicine" OR "virtual care" OR "virtual nursing" OR "virtual telepresence" OR "internet-based intervention" OR "internet intervention" OR "online intervention" OR "online-based intervention" OR "web-based intervention" OR "web intervention" OR "electronic consultation" OR cyberconsultation OR e-consultation OR econsultation OR "internet consultation" OR "internet-based consultation" OR "online consultation" OR "web consultation" OR "web-based consultation" OR "webbased consultation" OR "long distance consultation" OR "long-distance health care" OR "off-site health care" OR "off-site care") AND ("intensive care" OR icu OR icus OR "respiratory care unit" OR "coronary care unit" OR "burn unit" OR "acute trauma care" OR "trauma center" OR "trauma centre" OR "trauma unit" OR "trauma resuscitation unit" OR "acute care" OR "critical care" OR "critically ill" OR "critical illness" OR "acutely ill" OR "acute illness")))

= 491

--------------------------------------------------------------------------------------------------------------------------------------

**1b: Telemedicine/Acute diseases**

**Ovid MEDLINE(R) ALL**

1. telemedicine/ or telepathology/ or teleradiology/ or telerehabilitation/ or telemetry/

2. (telemedic* or tele-medic* or telemonitor* or tele-monitor* or telehealth or tele-health or telecare or tele-care or teleradiology or tele-radiology or telepathology or tele-pathology or telerehabilitation or tele-rehabilitation or telereferral or tele-referral or digital health or digital intervention* or remote intervention* or remote care or remote consultation* or remote counceling or remote monitoring or remote nursing or remote presence or remote screening or remote therapy or mobile health or mhealth or m-health or electronic health or ehealth or e-health or e-therapy or etherapy or electronic counceling or electronic monitoring or electronic intervention* or robotic telepresence or distance counceling or distance monitoring or virtual monitoring or virtual medicine or virtual care or virtual nursing or virtual telepresence or internet-based intervention* or internet intervention* or online intervention* or online-based intervention* or web-based intervention* or web intervention* or electronic consultation* or cyberconsultation* or e-consultation* or econsultation* or internet consultation* or internet-based consultation* or online consultation* or web consultation* or web-based consultation* or webbased consultation* or long distance consultation* or long-distance health care* or off-site health care* or off-site care).tw. (99746)

3. 1 or 2

4. intensive care units/ or burn units/ or coronary care units/ or recovery room/ or respiratory care units/

5. (intensive care or icu or icus or respiratory care unit* or coronary care unit* or burn unit* or acute trauma care or trauma cent* or trauma unit* or trauma resuscitation unit* or acute care or critical care or critically ill or critical illness or acutely ill or acute illness).tw.

6. 4 or 5

7. 3 and 6

8. (tele-ICU or tele-ICUs or teleICU or teleICUs or e-ICU or e-ICUs or eICU or eICUs or tele-intensive care or remote ICU or remote ICUs or electronic ICU or electronic ICUs or electronic intensive care or digital ICU or digital ICUs or digital intensive care or remote intensive care).tw.

9. 7 or 8

10. exp Sepsis/ or (sepsis or septicemia* or septic shock* or endotoxic shock* or endotoxin shock*).tw.

11. Respiratory Distress Syndrome/ or (ARDS or respiratory distress syndrome* or acute lung injur* or acute respiratory failur* or shock lung* or barotrauma).tw.

12. exp ventilation, artificial/ or (artificial ventilation* or artificial respiration* or mechanical ventilation*).tw. or (ventilator adj3 weaning*).tw.

13. Extracorporeal Membrane Oxygenation/ or (extracorporeal membrane oxygenation* or ECMO or extracorporeal life support* or ECLS).tw.

14. exp Multiple Trauma/ or exp Trauma Centers/ or polytrauma*.tw. or multiple trauma*.tw. or major trauma*.tw. or multiple injur*.tw. or multisystem trauma.tw. or trauma patient*.tw. or trauma population.tw. or trauma care.tw. or trauma cent*.tw. or exp Critical Illness/ or exp Critical Care/ or critical illness*.tw.

15. exp Multiple Organ Failure/ or (multiple organ failure* or multiorgan failure* or multi-organ failure* or multiorgan dysfunction* or multi-organ dysfunction* or multiple organ dysfunction*).tw.

16. exp Brain Injuries, Traumatic/ or (traumatic brain injur* or traumatic brain encephalopath* or brain trauma).tw.

17. cardiogenic shock/ or cardiogenic shock*.tw.

18. exp Liver Failure/ or (acute liver or acute hepatic or liver failure* or hepatic failure*).tw.

19. exp Acute Kidney Injury/ or (acute kidney or acute renal or kidney failure* or renal failure*).tw.

20. or/10-19

21. 3 and 20

22. 9 or 21

= 6423

**CENTRAL via Cochrane Library**

#1 MeSH descriptor: [Telemedicine] this term only

#2 MeSH descriptor: [Telepathology] this term only

#3 MeSH descriptor: [Teleradiology] this term only

#4 MeSH descriptor: [Telerehabilitation] this term only

#5 (telemedicine or tele-medic* or telemonitor* or tele-monitor* or telehealth or tele-health or telecare or tele-care or teleradiology or tele-radiology or telepathology or tele-pathology or telerehabilitation or tele-rehabilitation or telereferral or tele-referral or (digital NEXT health) or (digital NEXT intervention*) or (remote NEXT intervention*) or (remote NEXT care) or (remote NEXT consultation*) or (remote NEXT counceling) or (remote NEXT monitoring) or (remote NEXT nursing) or (remote NEXT presence) or (remote NEXT screening) or (remote NEXT therapy) or (mobile NEXT health) or mhealth or m-health or (electronic NEXT health) or ehealth or e-health or e-therapy or etherapy or (electronic NEXT counceling) or (electronic NEXT monitoring) or (electronic NEXT intervention*) or (robotic NEXT telepresence) or (distance NEXT counceling) or (distance NEXT monitoring) or (virtual NEXT monitoring) or (virtual NEXT medicine) or (virtual NEXT care) or (virtual NEXT nursing) or (virtual NEXT telepresence) or (internet-based NEXT intervention*) or (internet NEXT intervention*) or (online NEXT intervention*) or (online-based NEXT intervention*) or (web-based NEXT intervention*) or (web NEXT intervention*) or (electronic NEXT consultation*) or cyberconsultation* or e-consultation* or econsultation* or (internet NEXT consultation*) or (internet-based NEXT consultation*) or (online NEXT consultation*) or (web NEXT consultation*) or (web-based NEXT consultation*) or (webbased NEXT consultation*) or (long NEXT distance NEXT consultation*) or (long-distance NEXT health NEXT care*) or (off-site NEXT health NEXT care*) or (off-site NEXT care)):ti,ab,kw

#6 #1 or #2 or #3 or #4 or #5

#7 MeSH descriptor: [Intensive Care Units] this term only

#8 MeSH descriptor: [Burn Units] this term only

#9 MeSH descriptor: [Coronary Care Units] this term only

#10 MeSH descriptor: [Recovery Room] this term only

#11 ((intensive NEXT care) or icu or icus or (respiratory NEXT care NEXT unit*) or (coronary NEXT care NEXT unit*) or (burn NEXT unit*) or (acute NEXT trauma NEXT care) or (trauma NEXT cent*) or (trauma NEXT unit*) or (trauma NEXT resuscitation NEXT unit*) or (acute NEXT care) or (critical NEXT care) or (critically NEXT ill) or (critical NEXT illness) or (acutely NEXT ill) or (acute NEXT illness)):ti,ab,kw

#12 #7 or #8 or #9 or #10 or #11

#13 #6 and #12

#14 (tele-ICU or tele-ICUs or teleICU or teleICUs or e-ICU or e-ICUs or eICU or eICUs or (tele-intensive NEXT care) or (remote NEXT ICU) or (remote NEXT ICUs) or (electronic NEXT ICU) or (electronic NEXT ICUs) or (electronic NEXT intensive NEXT care) or (digital NEXT ICU) or (digital NEXT ICUs) or (digital NEXT intensive NEXT care) or (remote NEXT intensive NEXT care)):ti,ab,kw

#15 #13 or #14

#16 MeSH descriptor: [Sepsis] explode all trees

#17 (sepsis or septicemia* or (septic NEXT shock*) or (endotoxic NEXT shock*) or (endotoxin NEXT shock*)):ti,ab,kw

#18 MeSH descriptor: [Respiratory Distress Syndrome] explode all trees

#19 (ARDS or (respiratory NEXT distress NEXT syndrome*) or (acute NEXT lung NEXT injur*) or (acute NEXT respiratory NEXT failur*) or (shock NEXT lung*) or barotrauma):ti,ab,kw

#20 MeSH descriptor: [Ventilation] explode all trees

#21 ((artificial NEXT ventilation*) or (artificial NEXT respiration*) or (mechanical NEXT ventilation*) or (ventilator NEAR/3 weaning*)):ti,ab,kw

#22 MeSH descriptor: [Extracorporeal Membrane Oxygenation] explode all trees

#23 ((extracorporeal NEXT membrane NEXT oxygenation*) or ECMO or (extracorporeal NEXT life NEXT support*) or ECLS):ti,ab,kw

#24 MeSH descriptor: [Multiple Trauma] explode all trees

#25 MeSH descriptor: [Trauma Centers] explode all trees

#26 MeSH descriptor: [Critical Illness] explode all trees

#27 MeSH descriptor: [Critical Care] explode all trees

#28 (polytrauma* or (multiple NEXT trauma*) or (major NEXT trauma*) or (multiple NEXT injur*) or (multisystem NEXT trauma) or (trauma NEXT patient*) or (trauma NEXT population) or (trauma NEXT care) or (trauma NEXT cent*)):ti,ab,kw

#29 MeSH descriptor: [Multiple Organ Failure] explode all trees

#30 ((multiple NEXT organ NEXT failure*) or (multiorgan NEXT failure*) or (multi-organ NEXT failure*) or (multiorgan NEXT dysfunction*) or (multi-organ NEXT dysfunction*) or (multiple NEXT organ NEXT dysfunction*)):ti,ab,kw

#31 MeSH descriptor: [Brain Injuries, Traumatic] explode all trees

#32 ((traumatic NEXT brain NEXT injur*) or (traumatic NEXT brain NEXT encephalopath*) or (brain NEXT trauma)):ti,ab,kw

#33 MeSH descriptor: [Shock, Cardiogenic] this term only

#34 ((cardiogenic NEXT shock*)):ti,ab,kw

#35 MeSH descriptor: [Liver Failure] explode all trees

#36 ((acute NEXT liver) or (acute NEXT hepatic) or (liver NEXT failure*) or (hepatic NEXT failure*)):ti,ab,kw

#37 MeSH descriptor: [Acute Kidney Injury] explode all trees

#38 ((acute NEXT kidney) or (acute NEXT renal) or (kidney NEXT failure*) or (renal NEXT failure*)):ti,ab,kw

#39 #16 or #17 or #18 or #19 or #20 or #21 or #22 or #23 or #24 or #25 or #26 or #27 or #28 or #29 or #30 or #31 or #32 or #33 or #34 or #35 or #36 or #37 or #38

#40 #6 and #39

#41 #15 or #40

#42 #41 in Trials

= 1164

**Scopus**

**Part 1: Telemedicine / ICU / Tele-ICU**

(TITLE-ABS(telemedic* OR tele-medic* OR telemonitor* OR tele-monitor* OR telehealth OR tele-health OR telecare OR tele-care OR teleradiology OR tele-radiology OR telepathology OR tele-pathology OR telerehabilitation OR tele-rehabilitation OR telereferral OR tele-referral OR "digital health" OR "digital intervention*" OR "remote intervention*" OR "remote care" OR "remote consultation*" OR "remote counceling" OR "remote monitoring" OR "remote nursing" OR "remote presence" OR "remote screening" OR "remote therapy" OR "mobile health" OR mhealth OR m-health OR "electronic health" OR ehealth OR e-health OR e-therapy OR etherapy OR "electronic councel*" OR "electronic monitoring" OR "electronic intervention*" OR telepresence OR "distance councel*" OR "distance monitoring" OR "virtual monitoring" OR "virtual medicine" OR "virtual care" OR "virtual nursing" OR "virtual telepresence" OR "internet-based intervention*" OR "internet intervention*" OR "online intervention*" OR "online-based intervention*" OR "web-based intervention*" OR "web intervention*" OR "electronic consultation*" OR cyberconsultation* OR e-consultation* OR econsultation* OR "internet consultation*" OR "internet-based consultation*" OR "online consultation*" OR "web consultation*" OR "web-based consultation*" OR "webbased consultation*" OR "long distance consultation*" OR "long-distance health care*" OR "off-site health care*" OR "off-site care") AND TITLE-ABS("intensive care" OR icu OR icus OR "respiratory care unit*" OR "coronary care unit*" OR "burn unit*" OR "acute trauma care" OR "trauma cent*" OR "trauma unit*" OR "trauma resuscitation unit*" OR "acute care" OR "critical care" OR "critically ill" OR "critical illness" OR "acutely ill" OR "acute illness")) OR TITLE-ABS(tele-icu OR tele-icus OR teleicu OR teleicus OR e-icu OR e-icus OR eicu OR eicus OR "tele-intensive care" OR "remote ICU" OR "remote ICUs" OR "electronic ICU" OR "electronic ICUs" OR "electronic intensive care" OR "digital ICU" OR "digital ICUs" OR "digital intensive care" OR "remote care") AND TITLE-ABS-KEY("systematic review" OR "meta analysis" OR "meta-analysis")

=217

**Part 2: Telemedicine / Critical Illness, Acute Symptoms**

(TITLE-ABS(telemedic* OR tele-medic* OR telemonitor* OR tele-monitor* OR telehealth OR tele-health OR telecare OR tele-care OR teleradiology OR tele-radiology OR telepathology OR tele-pathology OR telerehabilitation OR tele-rehabilitation OR telereferral OR tele-referral OR "digital health" OR "digital intervention*" OR "remote intervention*" OR "remote care" OR "remote consultation*" OR "remote councel*" OR "remote monitoring" OR "remote nursing" OR "remote presence" OR "remote screening" OR "remote therapy" OR "mobile health" OR mhealth OR m-health OR "electronic health" OR ehealth OR e-health OR e-therapy OR etherapy OR "electronic councel*" OR "electronic monitoring" OR "electronic intervention*" OR telepresence OR "distance councel*" OR "distance monitoring" OR "virtual monitoring" OR "virtual medicine" OR "virtual care" OR "virtual nursing" OR "virtual telepresence" OR "internet-based intervention*" OR "internet intervention*" OR "online intervention*" OR "online-based intervention*" OR "web-based intervention*" OR "web intervention*" OR "electronic consultation*" OR cyberconsultation* OR e-consultation* OR econsultation* OR "internet consultation*" OR "internet-based consultation*" OR "online consultation*" OR "web consultation*" OR "web-based consultation*" OR "webbased consultation*" OR "long distance consultation*" OR "long-distance health care*" OR "off-site health care*" OR "off-site care") AND TITLE-ABS(ards OR "respiratory distress syndrome*" OR "acute lung injur*" OR "acute respiratory failur*" OR "acute respiratory distress*" OR "shock lung*" OR barotrauma OR "artificial ventilation*" OR "artificial respiration*" OR "mechanical ventilation*" OR "ventilator weaning*" OR "extracorporeal membrane oxygenation*" OR ecmo OR "extracorporeal life support" OR ecls OR polytrauma* OR "multiple trauma*" OR "major trauma*" OR "multiple injur*" OR "multisystem trauma" OR "trauma patient*" OR "trauma population" OR "trauma care" OR "trauma cent*" OR "critical illness*" OR "multiple organ failure*" OR "multiorgan failure*" OR "multi-organ failure*" OR "multiorgan dysfunction*" OR "multi-organ dysfunction*" OR "multiple organ dysfunction*" OR "traumatic brain injur*" OR "traumatic brain encephalopath*" OR "brain trauma" OR "liver failure*" OR "hepatic failure*" OR "acute liver" OR "acute hepatic" OR "acute kidney" OR "acute renal" OR "renal failure*" OR "kidney failure*")) AND TITLE-ABS-KEY("systematic review" OR "meta analysis" OR "meta-analysis")
=65

**CINAHL**

#1 (MH "Telemedicine+") OR (MH "Telenursing")

#2 TI ( telemedicine or tele-medicine or telemonitoring or tele-monitoring or telehealth or tele-health or telecare or tele-care or teleradiology or tele-radiology or telepathology or tele-pathology or telerehabilitation or tele-rehabilitation or telereferral or tele-referral or "digital health" or "digital intervention*" or "remote intervention*" or "remote care" or "remote consultation*" or "remote counceling" or "remote monitoring" or "remote nursing" or "remote presence" or "remote screening" or "remote therapy" or "mobile health" or mhealth or m-health or "electronic health" or ehealth or e-health or e-therapy or etherapy or "electronic counceling" or "electronic monitoring" or "electronic intervention*" or "robotic telepresence" or "distance counceling" or "distance monitoring" or "virtual monitoring" or "virtual medicine" or "virtual care" or "virtual nursing" or "virtual telepresence" or "internet-based intervention*" or "internet intervention*" or "online intervention*" or "online-based intervention*" or "web-based intervention*" or "web intervention*" or "electronic consultation*" or cyberconsultation* or e-consultation* or econsultation* or "internet consultation*" or "internet-based consultation*" or "online consultation*" or "web consultation*" or "web-based consultation*" or "webbased consultation*" or "long distance consultation*" or "long-distance health care*" or "off-site health care*" or "off-site care" ) OR AB ( telemedicine or tele-medicine or telemonitoring or tele-monitoring or telehealth or tele-health or telecare or tele-care or teleradiology or tele-radiology or telepathology or tele-pathology or telerehabilitation or tele-rehabilitation or telereferral or tele-referral or "digital health" or "digital intervention*" or "remote intervention*" or "remote care" or "remote consultation*" or "remote counceling" or "remote monitoring" or "remote nursing" or "remote presence" or "remote screening" or "remote therapy" or "mobile health" or mhealth or m-health or "electronic health" or ehealth or e-health or e-therapy or etherapy or "electronic counceling" or "electronic monitoring" or "electronic intervention*" or "robotic telepresence" or "distance counceling" or "distance monitoring" or "virtual monitoring" or "virtual medicine" or "virtual care" or "virtual nursing" or "virtual telepresence" or "internet-based intervention*" or "internet intervention*" or "online intervention*" or "online-based intervention*" or "web-based intervention*" or "web intervention*" or "electronic consultation*" or cyberconsultation* or e-consultation* or econsultation* or "internet consultation*" or "internet-based consultation*" or "online consultation*" or "web consultation*" or "web-based consultation*" or "webbased consultation*" or "long distance consultation*" or "long-distance health care*" or "off-site health care*" or "off-site care" )

#3 #1 OR #2

#4 (MH "Intensive Care Units") OR (MH "Coronary Care Units") OR (MH "Respiratory Care Units") OR (MH "Stroke Units")

#5 TI ("intensive care" or icu or icus or "respiratory care unit*" or "coronary care unit*" or "burn unit*" or "acute trauma care" or "trauma cent*" or "trauma unit*" or "trauma resuscitation unit*" or "acute care" or "critical care" or "critically ill" or "critical illness" or "acutely ill" or "acute illness" ) OR AB ("intensive care" or icu or icus or "respiratory care unit*" or "coronary care unit*" or "burn unit*" or "acute trauma care" or "trauma cent*" or "trauma unit*" or "trauma resuscitation unit*" or "acute care" or "critical care" or "critically ill" or "critical illness" or "acutely ill" or "acute illness" )

#6 #4 OR #5

#7 #3 AND #6

#8 TI (tele-icu OR tele-icus OR teleicu OR teleicus OR e-icu OR e-icus OR eicu OR eicus OR "tele-intensive care" OR "remote ICU" OR "remote ICUs" OR "electronic ICU" OR "electronic ICUs" OR "electronic intensive care" OR "digital ICU" OR "digital ICUs" OR "digital intensive care" OR "remote care") OR AB (tele-icu OR tele-icus OR teleicu OR teleicus OR e-icu OR e-icus OR eicu OR eicus OR "tele-intensive care" OR "remote ICU" OR "remote ICUs" OR "electronic ICU" OR "electronic ICUs" OR "electronic intensive care" OR "digital ICU" OR "digital ICUs" OR "digital intensive care" OR "remote care")

#9 #7 OR #8

#10 (MH "Sepsis+")

#11 TI (sepsis or septicemia* or "septic shock*" or "endotoxic shock*" or "endotoxin shock*") OR AB (sepsis or septicemia* or "septic shock*" or "endotoxic shock*" or "endotoxin shock*")

#12 (MH "Respiratory Distress Syndrome, Acute")

#13 Ti (ARDS or "respiratory distress syndrome*" or "acute lung injur*" or "acute respiratory failur*" or "shock lung*" or barotrauma) OR AB (ARDS or "respiratory distress syndrome*" or "acute lung injur*" or "acute respiratory failur*" or "shock lung*" or barotrauma)

#14 (MH "Ventilator Weaning") OR (MH "Ventilation, Mechanical, Differentiated")

#15 Ti ("artificial ventilation*" or "artificial respiration*" or "mechanical ventilation*" or "ventilator weaning*") OR AB ("artificial ventilation*" or "artificial respiration*" or "mechanical ventilation*" or "ventilator weaning*")

#16 (MH "Extracorporeal Carbon Dioxide Removal")

#17 TI ("extracorporeal membrane oxygenation*" or ECMO or "extracorporeal life support*" or ECLS ) OR AB ("extracorporeal membrane oxygenation*" or ECMO or "extracorporeal life support*" or ECLS)

#18 (MH "Multiple Trauma") OR (MH "Trauma Centers")

#19 Ti (polytrauma* or "multiple trauma*" or "major trauma*" or "multiple injur*" or "multisystem trauma" or "trauma patient*" or "trauma population" or "trauma care" or "trauma cent*") OR AB ( polytrauma* or "multiple trauma*" or "major trauma*" or "multiple injur*" or "multisystem trauma" or "trauma patient*" or "trauma population" or "trauma care" or "trauma cent*")

#20 (MH "Critical Illness") OR (MH "Critical Care")

#21 TI ("critical illness*" or "critical care") OR AB ("critical illness*" or "critical care")

#22 (MH "Multiple Organ Dysfunction Syndrome")

#23 TI ("multiple organ failure*" or "multiorgan failure*" or "multi-organ failure*" or "multiorgan dysfunction*" or "multi-organ dysfunction*" or "multiple organ dysfunction*" ) OR AB ( "multiple organ failure*" or "multiorgan failure*" or "multi-organ failure*" or "multiorgan dysfunction*" or "multi-organ dysfunction*" or "multiple organ dysfunction*")

#24 (MH "Brain Injuries")

#25 TI ("traumatic brain injur*" or "traumatic brain encephalopath*" or "brain trauma") OR AB ( traumatic brain injur* or traumatic brain encephalopath* or brain trauma)

#26 (MH "Shock, Cardiogenic")

#27 TI "cardiogenic shock*" OR AB "cardiogenic shock*"

#28 (MH "Liver Failure, Acute")

#29 TI ( "acute liver" or "acute hepatic" or "liver failure*" or "hepatic failure*" ) OR AB ( "acute liver" or "acute hepatic" or "liver failure*" or "hepatic failure*" )

#30 (MH "Kidney Failure, Acute")

#31 TI ( "acute kidney" or "acute renal" or "kidney failure*" or "renal failure*" ) OR AB ( "acute kidney" or "acute renal" or "kidney failure*" or "renal failure*" )

#32 S10 or S11 or S12 or S13 or S14 or S15 or S16 or S17 or S18 or S19 or S20 or S21 or S22 or S23 or S24 or S25 or S26 or S27 or S28 or S29 or S30 or S31

#33 S3 and S32

#34 S9 or S33

=3'858

**WHO ICTRP Database**

**Part 1: Telemedicine / ICU / Tele-ICU**

(tele-icu OR tele-icus OR teleicu OR teleicus OR e-icu OR e-icus OR eicu OR eicus OR "tele-intensive care" OR "remote ICU" OR "remote ICUs" OR "electronic ICU" OR "electronic ICUs" OR "electronic intensive care" OR "digital ICU" OR "digital ICUs" OR "digital intensive care" OR "remote care") OR ((telemedicine OR tele-medicine OR telemonitoring OR tele-monitoring OR telehealth OR tele-health OR telecare OR tele-care OR teleradiology OR tele-radiology OR telepathology OR tele-pathology OR telerehabilitation OR tele-rehabilitation OR telereferral OR tele-referral OR "digital health" OR "digital intervention" OR "remote intervention" OR "remote care" OR "remote consultation" OR "remote counceling" OR "remote monitoring" OR "remote nursing" OR "remote presence" OR "remote screening" OR "remote therapy" OR "mobile health" OR mhealth OR m-health OR "electronic health" OR ehealth OR e-health OR e-therapy OR etherapy OR "electronic counceling" OR "electronic monitoring" OR "electronic intervention" OR "robotic telepresence" OR "distance counceling" OR "distance monitoring" OR "virtual monitoring" OR "virtual medicine" OR "virtual care" OR "virtual nursing" OR "virtual telepresence" OR "internet-based intervention" OR "internet intervention" OR "online intervention" OR "online-based intervention" OR "web-based intervention" OR "web intervention" OR "electronic consultation" OR cyberconsultation OR e-consultation OR econsultation OR "internet consultation" OR "internet-based consultation" OR "online consultation" OR "web consultation" OR "web-based consultation" OR "webbased consultation" OR "long distance consultation" OR "long-distance health care" OR "off-site health care" OR "off-site care") AND ("intensive care" OR icu OR icus OR "respiratory care unit" OR "coronary care unit" OR "burn unit" OR "acute trauma care" OR "trauma center" OR "trauma centre" OR "trauma unit" OR "trauma resuscitation unit" OR "acute care" OR "critical care" OR "critically ill" OR "critical illness" OR "acutely ill" OR "acute illness"))
=137

**Part 2: Telemedicine / Critical Illness, Acute Symptoms**

(tele-icu OR tele-icus OR teleicu OR teleicus OR e-icu OR e-icus OR eicu OR eicus OR "tele-intensive care" OR "remote ICU" OR "remote ICUs" OR "electronic ICU" OR "electronic ICUs" OR "electronic intensive care" OR "digital ICU" OR "digital ICUs" OR "digital intensive care" OR "remote care") OR ((telemedicine OR tele-medicine OR telemonitoring OR tele-monitoring OR telehealth OR tele-health OR telecare OR tele-care OR teleradiology OR tele-radiology OR telepathology OR tele-pathology OR telerehabilitation OR tele-rehabilitation OR telereferral OR tele-referral OR "digital health" OR "digital intervention" OR "remote intervention" OR "remote care" OR "remote consultation" OR "remote counceling" OR "remote monitoring" OR "remote nursing" OR "remote presence" OR "remote screening" OR "remote therapy" OR "mobile health" OR mhealth OR m-health OR "electronic health" OR ehealth OR e-health OR e-therapy OR etherapy OR "electronic counceling" OR "electronic monitoring" OR "electronic intervention" OR "robotic telepresence" OR "distance counceling" OR "distance monitoring" OR "virtual monitoring" OR "virtual medicine" OR "virtual care" OR "virtual nursing" OR "virtual telepresence" OR "internet-based intervention" OR "internet intervention" OR "online intervention" OR "online-based intervention" OR "web-based intervention" OR "web intervention" OR "electronic consultation" OR cyberconsultation OR e-consultation OR econsultation OR "internet consultation" OR "internet-based consultation" OR "online consultation" OR "web consultation" OR "web-based consultation" OR "webbased consultation" OR "long distance consultation" OR "long-distance health care" OR "off-site health care" OR "off-site care") AND (ards OR "respiratory distress syndrome" OR "acute lung injury" OR "acute respiratory failure" OR "acute respiratory distress" OR "shock lung" OR barotrauma OR "artificial ventilation" OR "artificial respiration" OR "mechanical ventilation" OR "ventilator weaning" OR "extracorporeal membrane oxygenation" OR ecmo OR "extracorporeal life support" OR ecls OR polytrauma OR "multiple trauma" OR "major trauma" OR "multiple injuries" OR "multisystem trauma" OR "trauma patient" OR "trauma population" OR "trauma care" OR "trauma center" OR "trauma centre" OR "critical illness" OR "multiple organ failure" OR "multiorgan failure" OR "multi-organ failure" OR "multiorgan dysfunction" OR "multi-organ dysfunction" OR "multiple organ dysfunction" OR "traumatic brain injury" OR "traumatic brain encephalopathy" OR "brain trauma" OR "liver failure" OR "hepatic failure" OR "acute liver" OR "acute hepatic" OR "acute kidney" OR "acute renal" OR "renal failure" OR "kidney failure")))

=152

**ClinicalTrials.gov**

**Part 1: Telemedicine / ICU / Tele-ICU**

ALL ((tele-icu OR tele-icus OR teleicu OR teleicus OR e-icu OR e-icus OR eicu OR eicus OR "tele-intensive care" OR "remote ICU" OR "remote ICUs" OR "electronic ICU" OR "electronic ICUs" OR "electronic intensive care" OR "digital ICU" OR "digital ICUs" OR "digital intensive care" OR "remote care") OR ((telemedicine OR tele-medicine OR telemonitoring OR tele-monitoring OR telehealth OR tele-health OR telecare OR tele-care OR teleradiology OR tele-radiology OR telepathology OR tele-pathology OR telerehabilitation OR tele-rehabilitation OR telereferral OR tele-referral OR "digital health" OR "digital intervention" OR "remote intervention" OR "remote care" OR "remote consultation" OR "remote counceling" OR "remote monitoring" OR "remote nursing" OR "remote presence" OR "remote screening" OR "remote therapy" OR "mobile health" OR mhealth OR m-health OR "electronic health" OR ehealth OR e-health OR e-therapy OR etherapy OR "electronic counceling" OR "electronic monitoring" OR "electronic intervention" OR "robotic telepresence" OR "distance counceling" OR "distance monitoring" OR "virtual monitoring" OR "virtual medicine" OR "virtual care" OR "virtual nursing" OR "virtual telepresence" OR "internet-based intervention" OR "internet intervention" OR "online intervention" OR "online-based intervention" OR "web-based intervention" OR "web intervention" OR "electronic consultation" OR cyberconsultation OR e-consultation OR econsultation OR "internet consultation" OR "internet-based consultation" OR "online consultation" OR "web consultation" OR "web-based consultation" OR "webbased consultation" OR "long distance consultation" OR "long-distance health care" OR "off-site health care" OR "off-site care") AND ("intensive care" OR icu OR icus OR "respiratory care unit" OR "coronary care unit" OR "burn unit" OR "acute trauma care" OR "trauma center" OR "trauma centre" OR "trauma unit" OR "trauma resuscitation unit" OR "acute care" OR "critical care" OR "critically ill" OR "critical illness" OR "acutely ill" OR "acute illness")))

=529

**Part 2: Telemedicine / Critical Illness, Acute Symptoms**

ALL ((tele-icu OR tele-icus OR teleicu OR teleicus OR e-icu OR e-icus OR eicu OR eicus OR "tele-intensive care" OR "remote ICU" OR "remote ICUs" OR "electronic ICU" OR "electronic ICUs" OR "electronic intensive care" OR "digital ICU" OR "digital ICUs" OR "digital intensive care" OR "remote care") OR ((telemedicine OR tele-medicine OR telemonitoring OR tele-monitoring OR telehealth OR tele-health OR telecare OR tele-care OR teleradiology OR tele-radiology OR telepathology OR tele-pathology OR telerehabilitation OR tele-rehabilitation OR telereferral OR tele-referral OR "digital health" OR "digital intervention" OR "remote intervention" OR "remote care" OR "remote consultation" OR "remote counceling" OR "remote monitoring" OR "remote nursing" OR "remote presence" OR "remote screening" OR "remote therapy" OR "mobile health" OR mhealth OR m-health OR "electronic health" OR ehealth OR e-health OR e-therapy OR etherapy OR "electronic counceling" OR "electronic monitoring" OR "electronic intervention" OR "robotic telepresence" OR "distance counceling" OR "distance monitoring" OR "virtual monitoring" OR "virtual medicine" OR "virtual care" OR "virtual nursing" OR "virtual telepresence" OR "internet-based intervention" OR "internet intervention" OR "online intervention" OR "online-based intervention" OR "web-based intervention" OR "web intervention" OR "electronic consultation" OR cyberconsultation OR e-consultation OR econsultation OR "internet consultation" OR "internet-based consultation" OR "online consultation" OR "web consultation" OR "web-based consultation" OR "webbased consultation" OR "long distance consultation" OR "long-distance health care" OR "off-site health care" OR "off-site care") AND (ards OR "respiratory distress syndrome" OR "acute lung injury" OR "acute respiratory failure" OR "acute respiratory distress" OR "shock lung" OR barotrauma OR "artificial ventilation" OR "artificial respiration" OR "mechanical ventilation" OR "ventilator weaning" OR "extracorporeal membrane oxygenation" OR ecmo OR "extracorporeal life support" OR ecls OR polytrauma OR "multiple trauma" OR "major trauma" OR "multiple injuries" OR "multisystem trauma" OR "trauma patient" OR "trauma population" OR "trauma care" OR "trauma center" OR "trauma centre" OR "critical illness" OR "multiple organ failure" OR "multiorgan failure" OR "multi-organ failure" OR "multiorgan dysfunction" OR "multi-organ dysfunction" OR "multiple organ dysfunction" OR "traumatic brain injury" OR "traumatic brain encephalopathy" OR "brain trauma" OR "liver failure" OR "hepatic failure" OR "acute liver" OR "acute hepatic" OR "acute kidney" OR "acute renal" OR "renal failure" OR "kidney failure"))

=535

--------------------------------------------------------------------------------------------------------------------------------

**1c: 1a + 1b + RCT-Filter (limited to MEDLINE and CENTRAL)**

Search date: 26/10/2024

**Ovid MEDLINE(R) ALL**

**1**  telemedicine/ or telepathology/ or teleradiology/ or telerehabilitation/ or telemetry/
**2**  (telemedicine or tele-medicine or telemonitoring or tele-monitoring or telehealth or tele-health or telecare or tele-care or teleradiology or tele-radiology or telepathology or tele-pathology or telerehabilitation or tele-rehabilitation or telereferral or tele-referral or digital health or digital intervention* or remote intervention* or remote care or remote consultation* or remote counceling or remote monitoring or remote nursing or remote presence or remote screening or remote therapy or mobile health or mhealth or m-health or electronic health or ehealth or e-health or e-therapy or etherapy or electronic counceling or electronic monitoring or electronic intervention* or robotic telepresence or distance counceling or distance monitoring or virtual monitoring or virtual medicine or virtual care or virtual nursing or virtual telepresence or internet-based intervention* or internet intervention* or online intervention* or online-based intervention* or web-based intervention* or web intervention* or electronic consultation* or cyberconsultation* or e-consultation* or econsultation* or internet consultation* or internet-based consultation* or online consultation* or web consultation* or web-based consultation* or webbased consultation* or long distance consultation* or long-distance health care* or off-site health care* or off-site care).tw.
**3**  1 or 2
**4**  intensive care units/ or burn units/ or coronary care units/ or recovery room/ or respiratory care units/
**5**  (intensive care or icu or icus or respiratory care unit* or coronary care unit* or burn unit* or acute trauma care or trauma cent* or trauma unit* or trauma resuscitation unit* or acute care or critical care or critically ill or critical illness or acutely ill or acute illness).tw.
**6**  4 or 5
**7**  3 and 6
**8**  (tele-ICU or tele-ICUs or teleICU or teleICUs or e-ICU or e-ICUs or eICU or eICUs or tele-intensive care or remote ICU or remote ICUs or electronic ICU or electronic ICUs or electronic intensive care or digital ICU or digital ICUs or digital intensive care or remote intensive care).tw.
**9**  7 or 8
**10**  exp Sepsis/ or (sepsis or septicemia* or septic shock* or endotoxic shock* or endotoxin shock*).tw.
**11**  Respiratory Distress Syndrome/ or (ARDS or respiratory distress syndrome* or acute lung injur* or acute respiratory failur* or shock lung* or barotrauma).tw.
**12**  exp ventilation, artificial/ or (artificial ventilation* or artificial respiration* or mechanical ventilation*).tw. or (ventilator adj3 weaning*).tw.
**13**  Extracorporeal Membrane Oxygenation/ or (extracorporeal membrane oxygenation* or ECMO or extracorporeal life support* or ECLS).tw.
**14**  exp Multiple Trauma/ or exp Trauma Centers/ or polytrauma*.tw. or multiple trauma*.tw. or major trauma*.tw. or multiple injur*.tw. or multisystem trauma.tw. or trauma patient*.tw. or trauma population.tw. or trauma care.tw. or trauma cent*.tw. or exp Critical Illness/ or exp Critical Care/ or critical illness*.tw.
**15**  exp Multiple Organ Failure/ or (multiple organ failure* or multiorgan failure* or multi-organ failure* or multiorgan dysfunction* or multi-organ dysfunction* or multiple organ dysfunction*).tw.
**16**  exp Brain Injuries, Traumatic/ or (traumatic brain injur* or traumatic brain encephalopath* or brain trauma).tw.
**17**  cardiogenic shock/ or cardiogenic shock*.tw.
**18**  exp Liver Failure/ or (acute liver or acute hepatic or liver failure* or hepatic failure*).tw.
**19**  exp Acute Kidney Injury/ or (acute kidney or acute renal or kidney failure* or renal failure*).tw.
**20**  or/10-19
**21**  3 and 20
**22**  9 or 21

Cochrane RCT Filter, sensitivity max.

**23**  randomized controlled trial.pt.
**24**  controlled clinical trial.pt.
**25**  randomi?ed.ab.
**26**  placebo.ab.
**27**  drug therapy.fs.
**28**  randomly.ab.
**29**  trial.ab.
**30**  groups.ab.
**31**  or/23-30
**32**  exp animals/ not humans/
**33**  31 not 32
**34**  22 and 33

= 1545

**CENTRAL via Cochrane Library**

#1 MeSH descriptor: [Telemedicine] this term only

#2 MeSH descriptor: [Telepathology] this term only

#3 MeSH descriptor: [Teleradiology] this term only

#4 MeSH descriptor: [Telerehabilitation] this term only

#5 (telemedicine or tele-medicine or telemonitoring or tele-monitoring or telehealth or tele-health or telecare or tele-care or teleradiology or tele-radiology or telepathology or tele-pathology or telerehabilitation or tele-rehabilitation or telereferral or tele-referral or (digital NEXT health) or (digital NEXT intervention*) or (remote NEXT intervention*) or (remote NEXT care) or (remote NEXT consultation*) or (remote NEXT counceling) or (remote NEXT monitoring) or (remote NEXT nursing) or (remote NEXT presence) or (remote NEXT screening) or (remote NEXT therapy) or (mobile NEXT health) or mhealth or m-health or (electronic NEXT health) or ehealth or e-health or e-therapy or etherapy or (electronic NEXT counceling) or (electronic NEXT monitoring) or (electronic NEXT intervention*) or (robotic NEXT telepresence) or (distance NEXT counceling) or (distance NEXT monitoring) or (virtual NEXT monitoring) or (virtual NEXT medicine) or (virtual NEXT care) or (virtual NEXT nursing) or (virtual NEXT telepresence) or (internet-based NEXT intervention*) or (internet NEXT intervention*) or (online NEXT intervention*) or (online-based NEXT intervention*) or (web-based NEXT intervention*) or (web NEXT intervention*) or (electronic NEXT consultation*) or cyberconsultation* or e-consultation* or econsultation* or (internet NEXT consultation*) or (internet-based NEXT consultation*) or (online NEXT consultation*) or (web NEXT consultation*) or (web-based NEXT consultation*) or (webbased NEXT consultation*) or (long NEXT distance NEXT consultation*) or (long-distance NEXT health NEXT care*) or (off-site NEXT health NEXT care*) or (off-site NEXT care)):ti,ab,kw

#6 #1 or #2 or #3 or #4 or #5

#7 MeSH descriptor: [Intensive Care Units] this term only

#8 MeSH descriptor: [Burn Units] this term only

#9 MeSH descriptor: [Coronary Care Units] this term only

#10 MeSH descriptor: [Recovery Room] this term only

#11 ((intensive NEXT care) or icu or icus or (respiratory NEXT care NEXT unit*) or (coronary NEXT care NEXT unit*) or (burn NEXT unit*) or (acute NEXT trauma NEXT care) or (trauma NEXT cent*) or (trauma NEXT unit*) or (trauma NEXT resuscitation NEXT unit*) or (acute NEXT care) or (critical NEXT care) or (critically NEXT ill) or (critical NEXT illness) or (acutely NEXT ill) or (acute NEXT illness)):ti,ab,kw

#12 #7 or #8 or #9 or #10 or #11

#13 #6 and #12

#14 (tele-ICU or tele-ICUs or teleICU or teleICUs or e-ICU or e-ICUs or eICU or eICUs or (tele-intensive NEXT care) or (remote NEXT ICU) or (remote NEXT ICUs) or (electronic NEXT ICU) or (electronic NEXT ICUs) or (electronic NEXT intensive NEXT care) or (digital NEXT ICU) or (digital NEXT ICUs) or (digital NEXT intensive NEXT care) or (remote NEXT intensive NEXT care)):ti,ab,kw

#15 #13 or #14

#16 MeSH descriptor: [Sepsis] explode all trees

#17 (sepsis or septicemia* or (septic NEXT shock*) or (endotoxic NEXT shock*) or (endotoxin NEXT shock*)):ti,ab,kw

#18 MeSH descriptor: [Respiratory Distress Syndrome] explode all trees

#19 (ARDS or (respiratory NEXT distress NEXT syndrome*) or (acute NEXT lung NEXT injur*) or (acute NEXT respiratory NEXT failur*) or (shock NEXT lung*) or barotrauma):ti,ab,kw

#20 MeSH descriptor: [Ventilation] explode all trees

#21 ((artificial NEXT ventilation*) or (artificial NEXT respiration*) or (mechanical NEXT ventilation*) or (ventilator NEAR/3 weaning*)):ti,ab,kw

#22 MeSH descriptor: [Extracorporeal Membrane Oxygenation] explode all trees

#23 ((extracorporeal NEXT membrane NEXT oxygenation*) or ECMO or (extracorporeal NEXT life NEXT support*) or ECLS):ti,ab,kw

#24 MeSH descriptor: [Multiple Trauma] explode all trees

#25 MeSH descriptor: [Trauma Centers] explode all trees

#26 MeSH descriptor: [Critical Illness] explode all trees

#27 MeSH descriptor: [Critical Care] explode all trees

#28 (polytrauma* or (multiple NEXT trauma*) or (major NEXT trauma*) or (multiple NEXT injur*) or (multisystem NEXT trauma) or (trauma NEXT patient*) or (trauma NEXT population) or (trauma NEXT care) or (trauma NEXT cent*)):ti,ab,kw

#29 MeSH descriptor: [Multiple Organ Failure] explode all trees

#30 ((multiple NEXT organ NEXT failure*) or (multiorgan NEXT failure*) or (multi-organ NEXT failure*) or (multiorgan NEXT dysfunction*) or (multi-organ NEXT dysfunction*) or (multiple NEXT organ NEXT dysfunction*)):ti,ab,kw

#31 MeSH descriptor: [Brain Injuries, Traumatic] explode all trees

#32 ((traumatic NEXT brain NEXT injur*) or (traumatic NEXT brain NEXT encephalopath*) or (brain NEXT trauma)):ti,ab,kw

#33 MeSH descriptor: [Shock, Cardiogenic] this term only

#34 ((cardiogenic NEXT shock*)):ti,ab,kw

#35 MeSH descriptor: [Liver Failure] explode all trees

#36 ((acute NEXT liver) or (acute NEXT hepatic) or (liver NEXT failure*) or (hepatic NEXT failure*)):ti,ab,kw

#37 MeSH descriptor: [Acute Kidney Injury] explode all trees

#38 ((acute NEXT kidney) or (acute NEXT renal) or (kidney NEXT failure*) or (renal NEXT failure*)):ti,ab,kw

#39 #16 or #17 or #18 or #19 or #20 or #21 or #22 or #23 or #24 or #25 or #26 or #27 or #28 or #29 or #30 or #31 or #32 or #33 or #34 or #35 or #36 or #37 or #38

#40 #6 and #39

#41 #15 or #40

#42 #41 in Trials

= 1261

**1d: 1a + 1b + RCT-Filter (limited to MEDLINE and CENTRAL)**

Search date: 29/09/2025

Database: Ovid MEDLINE(R) ALL <1946 to September 26, 2025>

Search Strategy:

1 telemedicine/ or telepathology/ or teleradiology/ or telerehabilitation/ or telemetry/ (59524)

2 (telemedicine or tele-medicine or telemonitoring or tele-monitoring or telehealth or tele-health or telecare or tele-care or teleradiology or tele-radiology or telepathology or tele-pathology or telerehabilitation or tele-rehabilitation or telereferral or tele-referral or digital health or digital intervention* or remote intervention* or remote care or remote consultation* or remote counceling or remote monitoring or remote nursing or remote presence or remote screening or remote therapy or mobile health or mhealth or m-health or electronic health or ehealth or e-health or e-therapy or etherapy or electronic counceling or electronic monitoring or electronic intervention* or robotic telepresence or distance counceling or distance monitoring or virtual monitoring or virtual medicine or virtual care or virtual nursing or virtual telepresence or internet-based intervention* or internet intervention* or online intervention* or online-based intervention* or web-based intervention* or web intervention* or electronic consultation* or cyberconsultation* or e-consultation* or econsultation* or internet consultation* or internet-based consultation* or online consultation* or web consultation* or web-based consultation* or webbased consultation* or long distance consultation* or long-distance health care* or off-site health care* or off-site care).tw. (122817)

3 1 or 2 (147306)

4 intensive care units/ or burn units/ or coronary care units/ or recovery room/ or respiratory care units/ (88470)

5 (intensive care or icu or icus or respiratory care unit* or coronary care unit* or burn unit* or acute trauma care or trauma cent* or trauma unit* or trauma resuscitation unit* or acute care or critical care or critically ill or critical illness or acutely ill or acute illness).tw. (388669)

6 4 or 5 (404783)

7 3 and 6 (5250)

8 (tele-ICU or tele-ICUs or teleICU or teleICUs or e-ICU or e-ICUs or eICU or eICUs or tele-intensive care or remote ICU or remote ICUs or electronic ICU or electronic ICUs or electronic intensive care or digital ICU or digital ICUs or digital intensive care or remote intensive care).tw. (1093)

9 7 or 8 (6057)

10 exp Sepsis/ or (sepsis or septicemia* or septic shock* or endotoxic shock* or endotoxin shock*).tw. (248345)

11 Respiratory Distress Syndrome/ or (ARDS or respiratory distress syndrome* or acute lung injur* or acute respiratory failur* or shock lung* or barotrauma).tw. (77000)

12 exp ventilation, artificial/ or (artificial ventilation* or artificial respiration* or mechanical ventilation*).tw. or (ventilator adj3 weaning*).tw. (70951)

13 Extracorporeal Membrane Oxygenation/ or (extracorporeal membrane oxygenation* or ECMO or extracorporeal life support* or ECLS).tw. (28695)

14 exp Multiple Trauma/ or exp Trauma Centers/ or polytrauma*.tw. or multiple trauma*.tw. or major trauma*.tw. or multiple injur*.tw. or multisystem trauma.tw. or trauma patient*.tw. or trauma population.tw. or trauma care.tw. or trauma cent*.tw. or exp Critical Illness/ or exp Critical Care/ or critical illness*.tw. (182473)

15 exp Multiple Organ Failure/ or (multiple organ failure* or multiorgan failure* or multi-organ failure* or multiorgan dysfunction* or multi-organ dysfunction* or multiple organ dysfunction*).tw. (32832)

16 exp Brain Injuries, Traumatic/ or (traumatic brain injur* or traumatic brain encephalopath* or brain trauma).tw. (66404)

17 cardiogenic shock/ or cardiogenic shock*.tw. (21548)

18 exp Liver Failure/ or (acute liver or acute hepatic or liver failure* or hepatic failure*).tw. (60700)

19 exp Acute Kidney Injury/ or (acute kidney or acute renal or kidney failure* or renal failure*).tw. (176152)

20 or/10-19 (842702)

21 3 and 20 (3769)

22 9 or 21 (8025)

23 randomized controlled trial.pt. (646680)

24 controlled clinical trial.pt. (95742)

25 randomi?ed.ab. (846551)

26 placebo.ab. (262343)

27 drug therapy.fs. (2855421)

28 randomly.ab. (469684)

29 trial.ab. (776252)

30 groups.ab. (2919567)

31 or/23-30 (6440089)

32 exp animals/ not humans/ (5379039)

33 31 not 32 (5651745)

34 22 and 33

= 1836

**CENTRAL via Cochrane Library**

#1 MeSH descriptor: [Telemedicine] this term only 4704

#2 MeSH descriptor: [Telepathology] this term only 12

#3 MeSH descriptor: [Teleradiology] this term only 16

#4 MeSH descriptor: [Telerehabilitation] this term only 441

#5 (telemedicine or tele-medicine or telemonitoring or tele-monitoring or telehealth or tele-health or telecare or tele-care or teleradiology or tele-radiology or telepathology or tele-pathology or telerehabilitation or tele-rehabilitation or telereferral or tele-referral or (digital NEXT health) or (digital NEXT intervention*) or (remote NEXT intervention*) or (remote NEXT care) or (remote NEXT consultation*) or (remote NEXT counceling) or (remote NEXT monitoring) or (remote NEXT nursing) or (remote NEXT presence) or (remote NEXT screening) or (remote NEXT therapy) or (mobile NEXT health) or mhealth or m-health or (electronic NEXT health) or ehealth or e-health or e-therapy or etherapy or (electronic NEXT counceling) or (electronic NEXT monitoring) or (electronic NEXT intervention*) or (robotic NEXT telepresence) or (distance NEXT counceling) or (distance NEXT monitoring) or (virtual NEXT monitoring) or (virtual NEXT medicine) or (virtual NEXT care) or (virtual NEXT nursing) or (virtual NEXT telepresence) or (internet-based NEXT intervention*) or (internet NEXT intervention*) or (online NEXT intervention*) or (online-based NEXT intervention*) or (web-based NEXT intervention*) or (web NEXT intervention*) or (electronic NEXT consultation*) or cyberconsultation* or e-consultation* or econsultation* or (internet NEXT consultation*) or (internet-based NEXT consultation*) or (online NEXT consultation*) or (web NEXT consultation*) or (web-based NEXT consultation*) or (webbased NEXT consultation*) or (long NEXT distance NEXT consultation*) or (long-distance NEXT health NEXT care*) or (off-site NEXT health NEXT care*) or (off-site NEXT care)):ti,ab,kw 30263

#6 #1 or #2 or #3 or #4 or #5 30263

#7 MeSH descriptor: [Intensive Care Units] this term only 4254

#8 MeSH descriptor: [Burn Units] this term only 55

#9 MeSH descriptor: [Coronary Care Units] this term only 179

#10 MeSH descriptor: [Recovery Room] this term only 97

#11 ((intensive NEXT care) or icu or icus or (respiratory NEXT care NEXT unit*) or (coronary NEXT care NEXT unit*) or (burn NEXT unit*) or (acute NEXT trauma NEXT care) or (trauma NEXT cent*) or (trauma NEXT unit*) or (trauma NEXT resuscitation NEXT unit*) or (acute NEXT care) or (critical NEXT care) or (critically NEXT ill) or (critical NEXT illness) or (acutely NEXT ill) or (acute NEXT illness)):ti,ab,kw 55249

#12 #7 or #8 or #9 or #10 or #11 55339

#13 #6 and #12 776

#14 (tele-ICU or tele-ICUs or teleICU or teleICUs or e-ICU or e-ICUs or eICU or eICUs or (tele-intensive NEXT care) or (remote NEXT ICU) or (remote NEXT ICUs) or (electronic NEXT ICU) or (electronic NEXT ICUs) or (electronic NEXT intensive NEXT care) or (digital NEXT ICU) or (digital NEXT ICUs) or (digital NEXT intensive NEXT care) or (remote NEXT intensive NEXT care)):ti,ab,kw 75

#15 #13 or #14 842

#16 MeSH descriptor: [Sepsis] explode all trees 6603

#17 (sepsis or septicemia* or (septic NEXT shock*) or (endotoxic NEXT shock*) or (endotoxin NEXT shock*)):ti,ab,kw 17383

#18 MeSH descriptor: [Respiratory Distress Syndrome] explode all trees 3643

#19 (ARDS or (respiratory NEXT distress NEXT syndrome*) or (acute NEXT lung NEXT injur*) or (acute NEXT respiratory NEXT failur*) or (shock NEXT lung*) or barotrauma):ti,ab,kw 9956

#20 MeSH descriptor: [Ventilation] explode all trees 114

#21 ((artificial NEXT ventilation*) or (artificial NEXT respiration*) or (mechanical NEXT ventilation*) or (ventilator NEAR/3 weaning*)):ti,ab,kw 19471

#22 MeSH descriptor: [Extracorporeal Membrane Oxygenation] explode all trees 386

#23 ((extracorporeal NEXT membrane NEXT oxygenation*) or ECMO or (extracorporeal NEXT life NEXT support*) or ECLS):ti,ab,kw 1585

#24 MeSH descriptor: [Multiple Trauma] explode all trees 327

#25 MeSH descriptor: [Trauma Centers] explode all trees 310

#26 MeSH descriptor: [Critical Illness] explode all trees 3864

#27 MeSH descriptor: [Critical Care] explode all trees 3078

#28 (polytrauma* or (multiple NEXT trauma*) or (major NEXT trauma*) or (multiple NEXT injur*) or (multisystem NEXT trauma) or (trauma NEXT patient*) or (trauma NEXT population) or (trauma NEXT care) or (trauma NEXT cent*)):ti,ab,kw 4447

#29 MeSH descriptor: [Multiple Organ Failure] explode all trees 559

#30 ((multiple NEXT organ NEXT failure*) or (multiorgan NEXT failure*) or (multi-organ NEXT failure*) or (multiorgan NEXT dysfunction*) or (multi-organ NEXT dysfunction*) or (multiple NEXT organ NEXT dysfunction*)):ti,ab,kw 2420

#31 MeSH descriptor: [Brain Injuries, Traumatic] explode all trees 1861

#32 ((traumatic NEXT brain NEXT injur*) or (traumatic NEXT brain NEXT encephalopath*) or (brain NEXT trauma)):ti,ab,kw 5360

#33 MeSH descriptor: [Shock, Cardiogenic] this term only 522

#34 ((cardiogenic NEXT shock*)):ti,ab,kw 1719

#35 MeSH descriptor: [Liver Failure] explode all trees 1241

#36 ((acute NEXT liver) or (acute NEXT hepatic) or (liver NEXT failure*) or (hepatic NEXT failure*)):ti,ab,kw 3332

#37 MeSH descriptor: [Acute Kidney Injury] explode all trees 2499

#38 ((acute NEXT kidney) or (acute NEXT renal) or (kidney NEXT failure*) or (renal NEXT failure*)):ti,ab,kw 27032

#39 #16 or #17 or #18 or #19 or #20 or #21 or #22 or #23 or #24 or #25 or #26 or #27 or #28 or #29 or #30 or #31 or #32 or #33 or #34 or #35 or #36 or #37 or #38 85905

#40 #6 and #39 792

#41 #15 or #40 1390

#42 #41 in Trials **=** 1380
