# Supplementary material for: Telemedicine in adult intensive care: A systematic review of patient-relevant outcomes and methodological considerations
Source: PLOS Digit Health. 2025 Dec 15;4(12):e0001126. doi: 10.1371/journal.pdig.0001126 (PMC12704867; doi:10.1371/journal.pdig.0001126)
Supplement: S1 Table — (DOCX) [file pdig.0001126.s004.docx]

**Table 1: Excluded studies with reasons of the primary search.**

| Study ID | Title | Authors | Published Year | Journal | DOI | Reason for exlusion |
| --- | --- | --- | --- | --- | --- | --- |
| Alasheev 2016 | Case-control study of impact of telemedicine consultations on the outcome of patients with intracerebral hemorrhage | Alasheev, A.; Belkin, A.; Badaev, F.; Chadova, E. | 2016 | European stroke journal | 10.1177/2396987316642909 | Conference abstract |
| Blakesley 2006 | The buck stops in the E-ICU: increasing compliance with a sepsis bundle | Blakesley, D.; Fahey, F.; Hannah, R.; Tucker, K.; Roberts, G. | 2006 | Critical Care Nurse | NR | Conference abstract |
| Farrukh 2023 | 982: secure chat by telemedicine provider to improve sepsis bundle compliance: quality improvement study | Farrukh, Ayesha; James, Loretta; Palagiri, Ashok; Sadaka, Farid | 2023 | Critical Care Medicine | 10.1097/01.ccm.0000909656.02347.9a | Conference abstract |
| Kissee 2018 | (43) A randomized trial of pediatric critical care tele-emergency consultations | Kissee, J. L.; Kuppermann, N.; Dayal, P.; Sigal, I.; Tancredi, D.; Yoo, B. K.; Marcin, J. | 2018 | Pediatrics | 10.1542/peds.142.1_MeetingAbstract.53 | Conference abstract |
| Macauley 2020 | The effect of electronic icu implementation on weekend extubation rates | Macauley, Precious; Yaqoob, Hamid; Hafeez, Zeeshan; Chandy, Dipak; Becker, Christian | 2020 | CHEST | 10.1016/j.chest.2020.08.592 | Conference abstract |
| Mukherjee 2015 | Closing the Door to Tele-ICU: Analysis of Outcomes Before and After Closing a Single ICU to Tele-Medicine | Mukherjee, Sumit; Narsingam, Saiprasad; Hamarshi, Majdi; Ahmed, Zaheer | 2015 | CHEST | 10.1378/chest.2265062 | Conference abstract |
| Oyri 2005 | Application of technology across the life continuum: new technologies in the intensive care unit | Oyri, K. | 2005 | CONNECT: The World of Critical Care Nursing | NR | Conference abstract |
| Sackrowitz 2016 | Improving outcomes across a spectrum of hospitals | Sackrowitz, R. | 2016 | Critical care medicine | 10.1097/01.ccm.0000509873.37214.2f | Conference abstract |
| Yager 2012 | Comparison of face-to-face versus telemedicine patient assessment in a pediatric intensive care unit | Yager, P.; Dapul, H.; Murphy, S.; Clark, M.; Zheng, H.; Noviski, N. | 2012 | Critical care medicine | 10.1097/01.ccm.0000425605.04623.4b | Conference abstract |
| Yager 2013 | The reliability of telemedicine in assessing respiratory variables during patient assessment in a pediatric intensive care unit (PICU) | Yager, P.; Clark, M.; Noviski, N. | 2013 | Intensive care medicine | 10.1007/s00134-013-2950-8 | Conference abstract |
| Zaidi 2015 | Accuracy of Interpretation of Point of Care Ultrasound Images in Critically Ill Patients via Telemedicine | Zaidi, Gulrukh; Dhar, Sean; Chen, Lisa; Chandra, Saurabh; Koenig, Seth | 2015 | CHEST | 10.1378/chest.2250563 | Conference abstract |
| NR | Telemedicine Reduces Inter-Hospital Transfers | NR | 2018 | AARC Times | NR | Full text not retrievable |
| NR | Pharmacist role in remote care under investigation | NR | 2011 | Pharmacy News | NR | Full text not retrievable |
| NR | Study focuses on v-ICU practice | NR | 2006 | AACN News | NR | Full text not retrievable |
| Actrn 2012 | A randomised controlled trial to evaluate paediatric critical care telemedicine in Queensland | Actrn | 2012 | For children who have a remote intensive care consultation does telemedicine compared with standard telephone consultation improve clinical outcomes and provide economic savings to the health service? | NR | Full text not retrievable |
| Amba 2011 | Acute care NPs have new opportunities in virtual ICUs | Amba, Katheryne Tifuh | 2011 | Nurse Practitioner World News | NR | Full text not retrievable |
| Beck 2008 | Pediatrics E-link to critical care | Beck, L.; Freitag, C. | 2008 | Dynamics | NR | Full text not retrievable |
| Care 2001 | RC currents. Virtual doctors in the ICU | Care, American Association for Respiratory | 2001 | AARC Times | NR | Full text not retrievable |
| Childers 2002 | Instant messengers: tomorrow's telehealth technology has arrived in the ICU, allowing critical care teams to assess and treat patients near and far | Childers, L. | 2002 | NurseWeek (15475131) | NR | Full text not retrievable |
| Childers 2002 | Instant messengers: tomorrow's telehealth technology has arrived in the ICU, allowing critical care teams to assess and treat patients near and far | Childers, L. | 2002 | NurseWeek California | NR | Full text not retrievable |
| Drks 2023 | Usability, structural assessment, and self-reporting tools in Tele-Intensive Care Units | Drks | 2023 | Usability, structural assessment, and self-reporting tools in Tele-Intensive Care Units - U START TICU | NR | Full text not retrievable |
| Fahey 2013 | Tele-ICU: state of the art care | Fahey, A. J. | 2013 | Ohio Nurses Rev | NR | Full text not retrievable |
| Federwisch 2006 | From a distance: eICU system expands reach of high-level care | Federwisch, A. | 2006 | NurseWeek (15470571) | NR | Full text not retrievable |
| H 2016 | Telemedicine and the Seriously Ill Child | H, T. | 2016 | School Health Alert | NR | Full text not retrievable |
| Lucey 2006 | Electronic intensive care units | Lucey, J. F. | 2006 | Pediatrics | NR | Full text not retrievable |
| Meier 2006 | eICU...article about the eICU here at Sutter Health | Meier, B. A. | 2006 | NurseWeek California | NR | Full text not retrievable |
| Meyers 2006 | Remote control: nurses staff electronic ICUs to back up busy bedside RNs | Meyers, S. | 2006 | NurseWeek | NR | Full text not retrievable |
| Sloane 2008 | Robo-coworker: remote-presence robot attends patients at Ryder Trauma Center | Sloane, M. M. | 2008 | Nursing Spectrum -- Florida Edition | NR | Full text not retrievable |
| Veline 2009 | Through the wire. Telemedicine program helps improve critical care in rural region | Veline, J. | 2009 | Mod Healthc | NR | Full text not retrievable |
| Volkert 2004 | The future is now: remote monitoring of ICU patients comes to Wisconsin | Volkert, P. | 2004 | Nursingmatters | NR | Full text not retrievable |
| Wood 2005 | The eICU's eye never blinks | Wood, D. A. | 2005 | Nursing Spectrum -- Florida Edition | NR | Full text not retrievable |
| NR | Determining ICU Telemedicine Effectiveness | NR | 2019 | AACN Bold Voices | NR | Narrative review, commentary, editorial, etc. |
| NR | Telemedicine: Pharmaceutical experts support intensive care physicians | NR | 2019 | Anasthesiol. Intensivmed. Notf.med. Schmerzther. | 10.1055/a-0870-5982 | Narrative review, commentary, editorial, etc. |
| NR | We Have Consensus: TeleICU Continues to Evolve | NR | 2018 | AACN Bold Voices | NR | Narrative review, commentary, editorial, etc. |
| NR | Globalization of Healthcare: Treating ICU Patients Remotely | NR | 2018 | AACN Bold Voices | NR | Narrative review, commentary, editorial, etc. |
| NR | Critical Care Specialists Visit Remote Hospitals via Tele-ICU | NR | 2017 | AACN Bold Voices | NR | Narrative review, commentary, editorial, etc. |
| NR | AACN Tele-ICU Resources | NR | 2017 | AACN Bold Voices | NR | Narrative review, commentary, editorial, etc. |
| NR | AACN Resources for Care in Tele-ICUs | NR | 2016 | AACN Bold Voices | NR | Narrative review, commentary, editorial, etc. |
| NR | Expanding veterans' care access with tele-ICU | NR | 2015 | Health Management Technology | NR | Narrative review, commentary, editorial, etc. |
| NR | Reducing ICU Length of Stay: the Effect of Tele-ICU | NR | 2014 | Online Journal of Nursing Informatics | NR | Narrative review, commentary, editorial, etc. |
| NR | Tele-ICU Decreases Mortality, ICU Length of Stay | NR | 2014 | AACN Bold Voices | NR | Narrative review, commentary, editorial, etc. |
| NR | Aacn drives tele-ICU care | NR | 2011 | AACN Bold Voices | NR | Narrative review, commentary, editorial, etc. |
| NR | CCSC sets ICU telemedicine research agenda | NR | 2011 | AACN Bold Voices | NR | Narrative review, commentary, editorial, etc. |
| NR | Telecommunications and computerization in the ICU | NR | 2001 | Critical Care Medicine | NR | Narrative review, commentary, editorial, etc. |
| Akhtar 2012 | Tele-ICU: Is It Worth It? | Akhtar, Saadia R. | 2012 | Critical Care Alert | NR | Narrative review, commentary, editorial, etc. |
| Anonymous 2008 | The critical link. Remote monitoring of ICU patients can save lives and decrease patient length of stay across rural America | Anonymous | 2008 | Health Manag Technol | NR | Narrative review, commentary, editorial, etc. |
| Asplund 2000 | E-ICU. Hands-off care | Asplund, L. | 2000 | Hosp Health Netw | NR | Narrative review, commentary, editorial, etc. |
| Badawi 2015 | Telemedicine and the patient with sepsis | Badawi, O.; Hassan, E. | 2015 | Crit Care Clin | https://dx.doi.org/10.1016/j.ccc.2014.12.007 | Narrative review, commentary, editorial, etc. |
| Becker 2000 | Technology. Telemedicine system helps manage ICUs | Becker, C. | 2000 | Modern Healthcare | NR | Narrative review, commentary, editorial, etc. |
| Becker 2002 | Remote control. Specialists are running intensive-care units from remote sites via computers, and at least one health system with the e-ICU is reaping financial rewards--and saving lives | Becker, C. | 2002 | Mod Healthc | NR | Narrative review, commentary, editorial, etc. |
| Carlson 2015 | Telemedicine in the ICU | Carlson, R. W.; Scurlock, C. | 2015 | Crit Care Clin | https://dx.doi.org/10.1016/j.ccc.2015.01.001 | Narrative review, commentary, editorial, etc. |
| Caronia 2019 | EICU involvement improves compliance with sepsis bundle and substantially reduces in-hospital mortality in a tertiary-care teaching hospital | Caronia, Jonathan; Zheng, Huo Xiang; Fernandez, Sara; Schmid-Carbonaro, Robyn; Hertz, Craig | 2019 | CHEST | 10.1016/j.chest.2019.08.876 | Narrative review, commentary, editorial, etc. |
| Cohen 2019 | Telemedicine helps rural hospitals meet intensivist shortage | Cohen, Jessica Kim | 2019 | Modern Healthcare | NR | Narrative review, commentary, editorial, etc. |
| CotÃ®rlet 2014 | Use of telemedicine in intensive care unit wards - Case study from emergency hospital Moinesti 9 | CotÃ®rlet, A.; Tincu, E.; ZalÇŽ, A.; Andreianu, P. | 2014 | Arch. Balk. Med. Union | NR | Narrative review, commentary, editorial, etc. |
| Craig 2021 | Telemedicine consults to assess neonatal encephalopathy are feasible in the neonatal intensive care unit | Craig, A. K.; McAllister, L. M.; Evans, S.; Melendi, M. E. | 2021 | J Perinatol | https://dx.doi.org/10.1038/s41372-020-00828-3 | Narrative review, commentary, editorial, etc. |
| Cregan 2005 | The ViCCU Project -- achieving virtual presence using Ultrabroadband internet in a Critical Clinical application -- initial results | Cregan, P.; Stapleton, S.; Wilson, L.; Qiao, R. Y.; Li, J.; Percival, T. | 2005 | Stud Health Technol Inform | NR | Narrative review, commentary, editorial, etc. |
| Dharmar 2009 | Telemedicine for children in need of intensive care | Dharmar, M.; Smith, A. C.; Armfield, N. R.; Trujano, J.; Sadorra, C.; Marcin, J. P. | 2009 | Pediatr Ann | https://dx.doi.org/10.3928/00904481-20090918-08 | Narrative review, commentary, editorial, etc. |
| Dohmen 2021 | [Quality Improvement in Intensive Care Through Telemedicine: the TELnet@NRW Example] | Dohmen, S.; Benstoem, C.; Wahl, A.; Offermann, A.; Marx, G. | 2021 | Anasthesiol Intensivmed Notfallmed Schmerzther | https://dx.doi.org/10.1055/a-1288-4587 | Narrative review, commentary, editorial, etc. |
| Federation 2003 | Australia's most remote ICU upgrade ready | Federation, Australian Nursing; Midwifery | 2003 | Australian Nursing Journal | NR | Narrative review, commentary, editorial, etc. |
| Ferenc 2016 | Spreading telehealth know-how to other hospitals | Ferenc, J. | 2016 | Hosp Health Netw | NR | Narrative review, commentary, editorial, etc. |
| Ferguson 2016 | Intensive care: Using virtual consultations | Ferguson, Adele | 2016 | Kai Tiaki Nursing New Zealand | NR | Narrative review, commentary, editorial, etc. |
| Fraga 2012 | Evidence-based telemedicine: trauma & acute care surgery (EBT-TACS) | Fraga, G. P.; Nascimento, B., Jr.; Rizoli, S. | 2012 | Rev | NR | Narrative review, commentary, editorial, etc. |
| Gamble 2009 | Critical care network | Gamble, K. H. | 2009 | Healthcare Informatics | NR | Narrative review, commentary, editorial, etc. |
| Getz 2009 | Overnight sensation: technology allows doctors to remotely monitor ICU patients around the clock | Getz, L. | 2009 | For the Record (Great Valley Publishing Company, Inc.) | NR | Narrative review, commentary, editorial, etc. |
| Girard 2007 | Military and VA telemedicine systems for patients with traumatic brain injury | Girard, P. | 2007 | J Rehabil Res Dev | https://dx.doi.org/10.1682/jrrd.2006.12.0174 | Narrative review, commentary, editorial, etc. |
| Gorman 2011 | Tele-ICU comes of age: studies, hospital five-year results validate effectiveness of the technology | Gorman, M. J. | 2011 | Health Manag Technol | NR | Narrative review, commentary, editorial, etc. |
| GrahamIi 2022 | ICU telemedicine turns 40 | Graham Ii, Jeffrey D.; Peltan, Ithan D. | 2022 | Chest Physician | NR | Narrative review, commentary, editorial, etc. |
| Haranath 2021 | eNeuroIntensive Care in India: The Need of the Hour | Haranath, S. P.; Ganapathy, K.; Kesavarapu, S. R.; Kuragayala, S. D. | 2021 | Neurol India | https://dx.doi.org/10.4103/0028-3886.314591 | Narrative review, commentary, editorial, etc. |
| Hilker 2022 | Operational model to increase intensive care unit telemedicine capacity rapidly during a pandemic: experience in India | Hilker, S.; Mathias, S.; Anand, S.; Krishnamurthy, S. B.; Patel, S.; Unnikrishnan, D.; Raman, D.; Britto, C. D. | 2022 | Br J Anaesth | https://dx.doi.org/10.1016/j.bja.2022.02.036 | Narrative review, commentary, editorial, etc. |
| Hoesing 2009 | Question of the month. How would an electronic ICU affect your patient care? | Hoesing, G.; Connors, D. | 2009 | RN | NR | Narrative review, commentary, editorial, etc. |
| Joy 2011 | Journal watch | Joy, S. D. S. | 2011 | AJN American Journal of Nursing | NR | Narrative review, commentary, editorial, etc. |
| K 2012 | Remote intensive care monitoring is cost effective for sickest patients | K, B. | 2012 | AHRQ Research Activities | NR | Narrative review, commentary, editorial, etc. |
| Karpowicz 2008 | Continuum of competence: trials of educating in a Tele-ICU | Karpowicz, M. | 2008 | Critical Care Nurse | NR | Narrative review, commentary, editorial, etc. |
| Kashani 2019 | Telemedicine in Intensive Care Units: A Luxury or Necessity? | Kashani, K. B. | 2019 | Crit Care Clin | https://dx.doi.org/10.1016/j.ccc.2019.03.001 | Narrative review, commentary, editorial, etc. |
| Latifi 2015 | Telemedicine for trauma and intensive care: Changing the paradigm of telepresence | Latifi, R. | 2015 | Technol. Adv. in Surg., Trauma and Crit. Care | 10.1007/978-1-4939-2671-8_5 | Narrative review, commentary, editorial, etc. |
| Lawrence 2008 | I see you. As focus sharpens on the intensive care unit, remote monitoring capabilities and integration issues are under the microscope | Lawrence, D. | 2008 | Healthc Inform | NR | Narrative review, commentary, editorial, etc. |
| Leong 2005 | eICU program favorably affects clinical and economic outcomes | Leong, J. R.; Sirio, C. A.; Rotondi, A. J. | 2005 | Crit Care | NR | Narrative review, commentary, editorial, etc. |
| Lisk 2020 | Developing a Virtual Nursing Team to Support Predictive Analytics and Gaps in Patient Care | Lisk, L. E.; Buckley, J. D.; Wilson, K.; Martinez, V. A.; Cadiz, V. R.; Poropat, L.; Scruth, E. A. | 2020 | Clin Nurse Spec | https://dx.doi.org/10.1097/NUR.0000000000000496 | Narrative review, commentary, editorial, etc. |
| Loyola 2011 | An innovative approach to meeting early goal-directed therapy using telemedicine | Loyola, S.; Wilhelm, J.; Fornos, J. | 2011 | Crit Care Nurs Q | https://dx.doi.org/10.1097/CNQ.0b013e31821df105 | Narrative review, commentary, editorial, etc. |
| May 2002 | Remote ICU management: one healthcare system uses virtual rounds to help solve the intensivist shortage | May, S. | 2002 | Healthcare Informatics | NR | Narrative review, commentary, editorial, etc. |
| McCarten 2011 | Robots and remote monitoring programs earn national recognition | McCarten, Kim | 2011 | NurseZone Newsletter | NR | Narrative review, commentary, editorial, etc. |
| Morse 2014 | Tele-Intensivist Augmented Critical Care: Report of Three-Year Experience with a Remote Tele-ICU System | Morse, H. G.; Hunt, B.; Wheeler, C.; Kopec, I.; Hand, S.; Somers, W.; Tillirson, M. | 2014 | J S C Med Assoc | NR | Narrative review, commentary, editorial, etc. |
| Myers 2015 | Cost, Outcomes Mixed for Tele-ICU | Myers, N.; Wehrwein, P. | 2015 | Manag Care | NR | Narrative review, commentary, editorial, etc. |
| Penn 2015 | Intensive care gets a boost in several Arkansas hospitals. Baptist Health's eICU care services adding to care for critical care patients | Penn, C. L. | 2015 | J Ark Med Soc | NR | Narrative review, commentary, editorial, etc. |
| Peters 2004 | Con: Is the tele-intensive care unit ready for prime time? | Peters, S. G.; Farmer, J. C. | 2004 | Crit Care Med | NR | Narrative review, commentary, editorial, etc. |
| Pfrimmer 2011 | The Tele-ICU: a new dimension in critical care nursing education and practice | Pfrimmer, D. M.; Roslien, J. J. | 2011 | J Contin Educ Nurs | https://dx.doi.org/10.3928/00220124-20110722-03 | Narrative review, commentary, editorial, etc. |
| Popely 2009 | Telemedicine delivers healthy medical and financial benefits to ICUs | Popely, D. | 2009 | Healthc Exec | NR | Narrative review, commentary, editorial, etc. |
| Powell 2016 | Telemedicine to Reduce Medical Risk in Austere Medical Environments: The Virtual Critical Care Consultation (VC3) Service | Powell, D.; McLeroy, R. D.; Riesberg, J. C.; Vasios, W. N., 3rd; Miles, E. A.; Dellavolpe, J.; Keenan, S.; Pamplin, J. C. | 2016 | J Spec Oper Med | https://dx.doi.org/10.55460/6TPC-K6KL | Narrative review, commentary, editorial, etc. |
| Rajecki 2008 | eICU: Big brother, great friend. Remote monitoring of patients is a boon for nurses, patients, and families | Rajecki, R. | 2008 | Rn | NR | Narrative review, commentary, editorial, etc. |
| Roberts 2008 | Lights, camera, collaboration: implementation of an eICU orientation program | Roberts, G.; Dewoody, S. | 2008 | Critical Care Nurse | NR | Narrative review, commentary, editorial, etc. |
| Rosenberg 2016 | Telemedicine Seen as a Boon to Critical Care Nursing | Rosenberg, K. | 2016 | Am | https://dx.doi.org/10.1097/01.NAJ.0000482970.77413.70 | Narrative review, commentary, editorial, etc. |
| Rufo 2008 | Critical care: virtual ICUs, lower operational costs | Rufo, R. J. Z. | 2008 | Nursing Management | NR | Narrative review, commentary, editorial, etc. |
| Rufo 2011 | Tele-ICUs, part 2: Adding value to the health care equation | Rufo, R. | 2011 | Crit Care Nurs Q | https://dx.doi.org/10.1097/CNQ.0b013e3182201b7a | Narrative review, commentary, editorial, etc. |
| Rufo 2011 | Behind the scenes with integrated telemedicine | Rufo, Rebecca | 2011 | Nursing Management | dx.doi.org/special section | Narrative review, commentary, editorial, etc. |
| Runy 2005 | In box. Digital allies: Wisconsin hospitals come together to form a regional electronic intensive care unit | Runy, L. A. | 2005 | H&HN: Hospitals & Health Networks | NR | Narrative review, commentary, editorial, etc. |
| SchÃ¼rholz 2023 | Teleintensivmedizin bei Sepsis - Zukunftsvision oder schon Realität? | SchÃ¼rholz, Tobias; Dohmen, Sandra; Marx, Gernot | 2023 | DIVI | 10.53180/DIVI.2023.0220-0225 | Narrative review, commentary, editorial, etc. |
| Sheldon 2013 | Tele-ICU: Is This the Future? | Sheldon, Richard L. | 2013 | AARC Times | NR | Narrative review, commentary, editorial, etc. |
| Surapat 2021 | Role of clinical pharmacists in telemonitoring for patients with Coronavirus Disease 2019 (COVID-19) | Surapat, B.; Sungkanuparph, S.; Kirdlarp, S.; Lekpittaya, N.; Chunnguleum, K. | 2021 | J Clin Pharm Ther | https://dx.doi.org/10.1111/jcpt.13293 | Narrative review, commentary, editorial, etc. |
| Taylor 2013 | Improving the patient experience with an eICU. Telehealth helps provide round-the-clock patient care | Taylor, G. | 2013 | Healthc Exec | NR | Narrative review, commentary, editorial, etc.. |
| Thielst 2011 | Critical care 24/7: quick access brings better outcomes | Thielst, C. | 2011 | Health Manag Technol | NR | Narrative review, commentary, editorial, etc. |
| Trenary 2007 | Advances in technology affects nursing: iCare Intensive Care, Banner Health: remote telepresence in the critical care setting | Trenary, K. | 2007 | Arizona Nurse | NR | Narrative review, commentary, editorial, etc. |
| Varma 2015 | Telehealth in command | Varma, Manu | 2015 | Health Management Technology | NR | Narrative review, commentary, editorial, etc. |
| Venditti 2015 | Transformation of ICU and tele-ICU annual competencies | Venditti, A.; Edelstein, T.; Brock, A. J. | 2015 | Nurs Manage | https://dx.doi.org/10.1097/01.NUMA.0000471583.85819.f7 | Narrative review, commentary, editorial, etc. |
| Vespa 2005 | Robotic telepresence in the intensive care unit | Vespa, P. | 2005 | Crit Care | NR | Narrative review, commentary, editorial, etc. |
| Weiss 2021 | Qualitätssteigerung in der Intensivmedizin durch Telemedizin: Beispiel ERIC | Weiss, Björn; Paul, Nicolas; Kraufmann, Ben; Spies, Claudia D. | 2021 | AINS - Anästhesiologie Intensivmedizin Notfallmedizin Schmerztherapie | 10.1055/a-1130-4996 | Narrative review, commentary, editorial, etc. |
| Wetzel 2004 | Telemedicine and intensive care: are we ready and willing? | Wetzel, R. C. | 2004 | J Intensive Care Med | NR | Narrative review, commentary, editorial, etc. |
| Wilcox 2014 | Telemedicine in the intensive care unit: effect of a remote intensivist on outcomes | Wilcox, M. E.; Wiener-Kronish, J. P. | 2014 | JAMA Intern Med | https://dx.doi.org/10.1001/jamainternmed.2014.289 | Narrative review, commentary, editorial, etc. |
| Berrens 2019 | Efficacy and Safety of Pediatric Critical Care Physician Telemedicine Involvement in Rapid Response Team and Code Response in a Satellite Facility | Berrens, Z. J.; Gosdin, C. H.; Brady, P. W.; Tegtmeyer, K. | 2019 | Pediatr Crit Care Med | https://dx.doi.org/10.1097/PCC.0000000000001796 | Wrong comparator |
| Deisz 2019 | Additional Telemedicine Rounds as a Successful Performance-Improvement Strategy for Sepsis Management: Observational Multicenter Study | Deisz, R.; Rademacher, S.; Gilger, K.; Jegen, R.; Sauerzapfe, B.; Fitzner, C.; Stoppe, C.; Benstoem, C.; Marx, G. | 2019 | J Med Internet Res | https://dx.doi.org/10.2196/11161 | Wrong comparator |
| Mullen-Fortino 2012 | Innovative use of tele-ICU in long-term acute care hospitals | Mullen-Fortino, M.; Sites, F. D.; Soisson, M.; Galen, J. | 2012 | AACN Adv Crit Care | NR | Wrong comparator |
| Rincon 2011 | Standardizing sepsis screening and management via a tele-ICU program improves patient care | Rincon, T. A.; Bourke, G.; Seiver, A. | 2011 | Telemed J E Health | https://dx.doi.org/10.1089/tmj.2010.0225 | Wrong comparator |
| Scudeller 2023 | Tele-Intensive Care Unit Program in Brazil: Implementation and Expansion | Scudeller, P. G.; Lamas, C. A.; Alvarenga, A. M.; Garcia, M. L.; Amaral, T. F.; de Oliveira, M. R.; de Macedo, B. R.; Testa, C. B.; Baptista, F. S.; Francisco, R. P. V.; de Carvalho, C. R. R. | 2023 | Telemed Rep | https://dx.doi.org/10.1089/tmr.2023.0017 | Wrong comparator |
| Williams 2019 | A Mixed Methods Study of Tele-ICU Nursing Interventions to Prevent Failure to Rescue of Patients in Critical Care | Williams, L. S.; Johnson, E.; Armaignac, D. L.; Nemeth, L. S.; Magwood, G. S. | 2019 | Telemed J E Health | https://dx.doi.org/10.1089/tmj.2018.0086 | Wrong comparator |
| Brigham 2026 | Real-time Symptom Monitoring Using ePROs to Prevent Adverse Events During Care Transitions | Brigham; Women's, Hospital; Rand | 2026 | NR | NR | Wrong Intervention |
| Cedars-SinaiMedical 2021 | Utilizing Technology for Optimization of Pain Management and Mobilization in High Risk Cardiac Surgical ICU Patients | Cedars-Sinai Medical, Center | 2021 | NR | NR | Wrong Intervention |
| Columbia 2024 | Sepsis Electronic Prompting for Timely Intervention and Care for Inpatients | Columbia, University | 2024 | NR | NR | Wrong Intervention |
| Gregory 2018 | Study protocol for the Anesthesiology Control Tower-Feedback Alerts to Supplement Treatments (ACTFAST-3) trial: a pilot randomized controlled trial in intraoperative telemedicine | Gregory, S.; Murray-Torres, T. M.; Fritz, B. A.; Ben Abdallah, A.; Helsten, D. L.; Wildes, T. S.; Sharma, A.; Avidan, M. S. | 2018 | F1000Res | https://dx.doi.org/10.12688/f1000research.14897.2 | Wrong Intervention |
| KanuniSultanSuleyman 2020 | Experience of an Emergency Intensive Care Unit During COVID-19 Pandemic | Kanuni Sultan Suleyman, Training; Research, Hospital | 2020 | NR | NR | Wrong Intervention |
| MedicalUniversity 2025 | Development and Validation of a Telemonitoring System for High-risk Cardiovascular Patients | Medical University, Innsbruck | 2025 | NR | NR | Wrong Intervention |
| NationalCheng-KungUniversity 2021 | i-Dashboard for Multi-disciplinary Rounds in SICU | National Cheng-Kung University, Hospital | 2021 | NR | NR | Wrong Intervention |
| PaulsStradinsClinicalUniversity 2023 | Telemedical Assistance in Automatic Titration of Oxygen for Intensive Care Patients | Pauls Stradins Clinical University, Hospital | 2023 | NR | NR | Wrong Intervention |
| Universityof 2023 | Facilitating Communication Study | University of, Washington | 2023 | NR | NR | Wrong Intervention |
| Universityof 2025 | A Pilot Randomized Controlled Trial: CoINTEGRATE | University of, Michigan | 2025 | NR | NR | Wrong Intervention |
| UniversityofCalifornia 2022 | UC Health Care Planning Study | University of California, Los Angeles; University of California, San Francisco; University of California, Irvine | 2022 | NR | NR | Wrong Intervention |
| DragiÄ‡ 2016 | Video consultation in a medical intensive care unit based on CERTAIN platform- a pilot study | Dragić, S.; Kovačević , P.; Momčičević, D.; Zlojutro, B.; Topolovac, S.; Đajić, V.; Kovačević, T.; Festić, E.; Gajić, O. | 2016 | Infektol. Glas. | NR | Wrong language |
| Li 2013 | [Implementation of telemedicine services in the earthquake disaster relief: the best medical experts provide direct medical service to the affected people] | Li, T. S.; Chai, J. K. | 2013 | Zhonghua Wei Zhong Bing Ji Jiu Yi Xue | https://dx.doi.org/10.3760/cma.j.issn.2095-4352.2013.05.003 | Wrong language |
| Mekhantieva 2021 | Experience of applying telemedicine technologies in the work of voronezh regional clinical center for disaster medicine | Mekhantieva, L. E.; Artemov, A. N.; Ilyichev, V. P. | 2021 | Medicina Katastrof | 10.33266/2070-1004-2021-4-17-22 | Wrong language |
| Arneson 2020 | Answering the Call: Impact of Tele-ICU Nurses During the COVID-19 Pandemic | Arneson, S. L.; Tucker, S. J.; Mercier, M.; Singh, J. | 2020 | Crit Care Nurse | https://dx.doi.org/10.4037/ccn2020126 | Wrong outcomes |
| Berg 2003 | Remote critical care consultation: telehealth projection of clinical specialty expertise | Berg, B. W.; Vincent, D. S.; Hudson, D. A. | 2003 | J Telemed Telecare |  | Wrong outcomes |
| Chandra 2021 | Collaboration Between Tele-ICU Programs Has the Potential to Rapidly Increase the Availability of Critical Care Physicians-Our Experience Was During Coronavirus Disease 2019 Nomenclature | Chandra, S.; Hertz, C.; Khurana, H.; Doerfler, M. E. | 2021 | Crit | https://dx.doi.org/10.1097/CCE.0000000000000363 | Wrong outcomes |
| Fadaizadeh 2018 | Role of Telemedicine in Pace of Consultation and Physicians' Satisfaction in Thoracic Surgery ICU | Fadaizadeh, L.; Shajareh, E.; Taheri, M. J.; Heydari, G.; Fazanegan, B.; Sistani, M. | 2018 | Tanaffos | NR | Wrong outcomes |
| Fang 2023 | Evaluating the feasibility of a multicenter teleneonatology clinical effectiveness trial | Fang, J. L.; Umoren, R. A.; Whyte, H.; Limjoco, J.; Makkar, A.; Behl, S.; Lo, M. D.; White, L.; Culjat, M.; Taylor, J. S.; Kathuria, S.; Webb, M. O.; Schad, T.; Shafranski, S.; Yankanah, R.; Herrin, J.; Demaerschalk, B. M. | 2023 | Pediatr Res | https://dx.doi.org/10.1038/s41390-023-02659-2 | Wrong outcomes |
| Kalb 2015 | Increasing quality through telemedicine in the intensive care unit | Kalb, T. H. | 2015 | Crit Care Clin | https://dx.doi.org/10.1016/j.ccc.2014.12.005 | Wrong outcomes |
| McLeroy 2022 | Advanced Virtual Support for Operational Forces: A 3-Year Summary | McLeroy, R. D.; Kile, M. T.; Yourk, D.; Hipp, S.; Pamplin, J. C. | 2022 | Mil Med | https://dx.doi.org/10.1093/milmed/usab388 | Wrong outcomes |
| Meidl 2008 | Implementation of pharmacy services in a telemedicine intensive care unit | Meidl, T. M.; Woller, T. W.; Iglar, A. M.; Brierton, D. G. | 2008 | Am J Health-Syst Pharm | https://dx.doi.org/10.2146/ajhp070682 | Wrong outcomes |
| Melo 2019 | Telemonitoring of Neonatal Intensive Care Units: Preliminary Experience in the State of Minas Gerais, Brazil | Melo, Mdcb; Santos, A. F.; Alves, H. J.; Queiroz, T. C. N.; Silva, N. L. C. | 2019 | Am J Perinatol | https://dx.doi.org/10.1055/s-0038-1668558 | Wrong outcomes |
| Otero 2014 | International telemedicine in pediatric cardiac critical care: a multicenter experience | Otero, A. V.; Lopez-Magallon, A. J.; Jaimes, D.; Motoa, M. V.; Ruz, M.; Erdmenger, J.; Munoz, R. A. | 2014 | Telemed J E Health | https://dx.doi.org/10.1089/tmj.2013.0307 | Wrong outcomes |
| Rak 2017 | Identifying Strategies for Effective Telemedicine Use in Intensive Care Units: The ConnECCT Study Protocol | Rak, K. J.; Kuza, C. C.; Ashcraft, L. E.; Morrison, P. K.; Angus, D. C.; Barnato, A. E.; Hravnak, M.; Hershey, T. B.; Kahn, J. M. | 2017 | Int | https://dx.doi.org/10.1177/1609406917733387 | Wrong outcomes |
| Ramnath 2021 | Designing a critical care solution using in-person and telemedicine approaches in the US-Mexico border area during COVID-19 | Ramnath, V. R.; Hill, L.; Schultz, J.; Mandel, J.; Smith, A.; Holberg, S.; Horton, L. E.; Malhotra, A.; Friedman, L. S. | 2021 | Health Policy Open | https://dx.doi.org/10.1016/j.hpopen.2021.100051 | Wrong outcomes |
| Scales 2011 | A Multifaceted Intervention for Quality Improvement in a Network of Intensive Care Units: A Cluster Randomized Trial | Scales, Damon C.; Dainty, Katie; Hales, Brigette; Pinto, Ruxandra; Fowler, Robert A.; Adhikari, Neill K. J.; Zwarenstein, Merrick | 2011 | JAMA | 10.1001/jama.2010.2000 | Wrong outcomes |
| Scales 2011 | An innovative telemedicine knowledge translation program to improve quality of care in intensive care units: protocol for a cluster randomized pragmatic trial | Scales, D. C.; Dainty, K.; Hales, B.; Pinto, R.; Fowler, R. A.; Adhikari, N. K.; Zwarenstein, M. | 2009 | Implement Sci | https://dx.doi.org/10.1186/1748-5908-4-5 | Wrong outcomes |
| Singh 2010 | Four years of experience of telemedicine for paediatric care in three Punjab hospitals, North India: achievements and lessons | Singh, M.; Das, R. R. | 2010 | Postgrad Med J | https://dx.doi.org/10.1136/pgmj.2009.082735 | Wrong outcomes |
| Singh 2021 | Telecritical Care Clinical and Operational Strategies in Response to COVID-19 | Singh, J.; Green, M. B.; Lindblom, S.; Reif, M. S.; Thakkar, N. P.; Papali, A. | 2021 | Telemed J E Health | https://dx.doi.org/10.1089/tmj.2020.0186 | Wrong outcomes |
| UniversityofBritish 2017 | The Monitoring Messenger: Mobile Patient Monitoring for the Pediatric Intensive Care Unit | University of British, Columbia | 2017 | NR | NR | Wrong outcomes |
| Wenger 2014 | Telemedicine for genetic and neurologic evaluation in the neonatal intensive care unit | Wenger, T. L.; Gerdes, J.; Taub, K.; Swarr, D. T.; Deardorff, M. A.; Abend, N. S. | 2014 | J Perinatol | https://dx.doi.org/10.1038/jp.2013.159 | Wrong outcomes |
| Albritton 2018 | The Effect Of A Newborn Telehealth Program On Transfers Avoided: A Multiple-Baseline Study | Albritton, J.; Maddox, L.; Dalto, J.; Ridout, E.; Minton, S. | 2018 | Health Aff (Millwood) | https://dx.doi.org/10.1377/hlthaff.2018.05133 | Wrong population |
| Armfield 2014 | The effectiveness of telemedicine for paediatric retrieval consultations: rationale and study design for a pragmatic multicentre randomised controlled trial | Armfield, N. R.; Coulthard, M. G.; Slater, A.; McEniery, J.; Elcock, M.; Ware, R. S.; Scuffham, P. A.; Bensink, M. E.; Smith, A. C. | 2014 | BMC Health Serv Res | https://dx.doi.org/10.1186/s12913-014-0546-9 | Wrong population |
| Bell 2016 | Telemedicine Versus Face-to-Face Evaluations by Respiratory Therapists of Mechanically Ventilated Neonates and Children: A Pilot Study | Bell, R. C.; Yager, P. H.; Clark, M. E.; Roumiantsev, S.; Venancio, H. L.; Chipman, D. W.; Kacmarek, R. M.; Noviski, N. N. | 2016 | Respir Care | https://dx.doi.org/10.4187/respcare.04080 | Wrong population |
| Coffey 2022 | Telemedicine Consultation to Assess Neonatal Encephalopathy in Rural Community Hospitals and Tertiary Care Centers | Coffey, R.; Melendi, M.; Cutler, A. K.; Craig, A. K. | 2022 | J | https://dx.doi.org/10.46804/2641-2225.1115 | Wrong population |
| Cooper 2023 | Quality Indices and Outcomes of a Neonatology Telerounding Program in a Level II Neonatal Intensive Care Unit: Single-Center Experience during the COVID-19 Pandemic | Cooper, C.; Mastroianni, R.; Bosque, E.; Chabra, S.; Campbell, J.; Perez, J. A.; White, C. F.; James, J. E.; Umoren, R. A. | 2023 | Am J Perinatol | https://dx.doi.org/10.1055/a-2115-8530 | Wrong population |
| Ctri 2022 | Effect of Tele-medicine in addition to standard monitoring and care in management and outcome of paediatric ICU patients: a tertiary care hospital based,open-label randomized control trial | Ctri | 2022 | https://trialsearch.who.int/Trial2.aspx?TrialID=CTRI/2022/12/047927 | NR | Wrong population |
| Garingo 2012 | The use of mobile robotic telemedicine technology in the neonatal intensive care unit | Garingo, A.; Friedlich, P.; Tesoriero, L.; Patil, S.; Jackson, P.; Seri, I. | 2012 | J Perinatol | https://dx.doi.org/10.1038/jp.2011.72 | Wrong population |
| Ghbeis 2018 | Tele-Pediatric Intensive Care for Critically Ill Children in Syria | Ghbeis, M. B.; Steffen, K. M.; Braunlin, E. A.; Beilman, G. J.; Dahman, J.; Ostwani, W.; Steiner, M. E. | 2018 | Telemed J E Health | https://dx.doi.org/10.1089/tmj.2017.0216 | Wrong population |
| Gitaka 2018 | Evaluating quality neonatal care, call Centre service, tele-health and community engagement in reducing newborn morbidity and mortality in Bungoma county, Kenya | Gitaka, J.; Natecho, A.; Mwambeo, H. M.; Gatungu, D. M.; Githanga, D.; Abuya, T. | 2018 | BMC Health Serv Res | https://dx.doi.org/10.1186/s12913-018-3293-5 | Wrong population |
| Hall 2010 | Neonatal regionalization through telemedicine using a community-based research and education core facility | Hall, R. W.; Hall-Barrow, J.; Garcia-Rill, E. | 2010 | Ethn Dis | NR | Wrong population |
| Harvey 2017 | The Impact of Telemedicine on Pediatric Critical Care Triage | Harvey, J. B.; Yeager, B. E.; Cramer, C.; Wheeler, D.; McSwain, S. D. | 2017 | Pediatr Crit Care Med | https://dx.doi.org/10.1097/PCC.0000000000001330 | Wrong population |
| Haynes 2021 | The Use of Telemedicine for Stabilization of Neonates Transferred from Rural Community Hospitals | Haynes, S. C.; Hoffman, K. R.; Patel, S.; Smith, S.; Romano, P. S.; Marcin, J. P. | 2021 | Telemed J E Health | https://dx.doi.org/10.1089/tmj.2020.0503 | Wrong population |
| HospitalMoinhosde 2022 | Evaluation of the Impact of Telemedicine in Pediatric Intensive Care Units | Hospital Moinhos de, Vento | 2022 | NR | NR | Wrong population |
| HospitalMoinhosde 2022 | Evaluation of the Impact of Telemedicine in Pediatric Intensive Care Units: cluster Randomized Controlled Trial | Nct | 2022 | https://clinicaltrials.gov/show/NCT05260710 | NR | Wrong population |
| Jagarapu 2024 | TeleNICU: Extending the reach of level IV care and optimizing the triage of patient transfers | Jagarapu, J.; Kapadia, V.; Mir, I.; Kakkilaya, V.; Carlton, K.; Fokken, M.; Brown, S.; Hall-Barrow, J.; Savani, R. C. | 2024 | J Telemed Telecare | https://dx.doi.org/10.1177/1357633X211038153 | Wrong population |
| Kawaguchi 2021 | Remote triage in paediatric critical care: A Canadian provincial-wide cohort study | Kawaguchi, Atsushi; Guerra, Gonzalo Garcia; Gilad, Eli; Jain, Praveen; DeCaen, Allan | 2021 | Paediatrics & Child Health (1205-7088) | 10.1093/pch/pxaa036 | Wrong population |
| Kim 2013 | Telemedicine collaboration improves perinatal regionalization and lowers statewide infant mortality | Kim, E. W.; Teague-Ross, T. J.; Greenfield, W. W.; Keith Williams, D.; Kuo, D.; Hall, R. W. | 2013 | J Perinatol | https://dx.doi.org/10.1038/jp.2013.37 | Wrong population |
| Kovacikova 2017 | Transatlantic medical consultation and second opinion in pediatric cardiology has benefit past patient care: A case study in videoconferencing | Kovacikova, L.; Zahorec, M.; Skrak, P.; Hanna, B. D.; Lee Vogel, R. | 2017 | Congenit | https://dx.doi.org/10.1111/chd.12480 | Wrong population |
| Labarbera 2013 | The impact of telemedicine intensivist support and a pediatric hospitalist program on a community hospital | Labarbera, J. M.; Ellenby, M. S.; Bouressa, P.; Burrell, J.; Flori, H. R.; Marcin, J. P. | 2013 | Telemed J E Health | https://dx.doi.org/10.1089/tmj.2012.0303 | Wrong population |
| Lopez-Magallon 2015 | Patient Outcomes of an International Telepediatric Cardiac Critical Care Program | Lopez-Magallon, A. J.; Otero, A. V.; Welchering, N.; Bermon, A.; Castillo, V.; Duran, A.; Castro, J.; Munoz, R. | 2015 | Telemed J E Health | https://dx.doi.org/10.1089/tmj.2014.0188 | Wrong population |
| Lopez-Magallon 2018 | Telemedicine in Pediatric Critical Care: A Retrospective Study in an International Extracorporeal Membrane Oxygenation Program | Lopez-Magallon, A. J.; Saenz, L.; Lara Gutierrez, J.; Florez, C. X.; Althouse, A. D.; Sharma, M. S.; Duran, A.; Salazar, L.; Munoz, R. | 2018 | Telemed J E Health | https://dx.doi.org/10.1089/tmj.2017.0223 | Wrong population |
| Maddox 2021 | Implementation and Outcomes of a Telehealth Neonatology Program in a Single Healthcare System | Maddox, L. J.; Albritton, J.; Morse, J.; Latendresse, G.; Meek, P.; Minton, S. | 2021 | Front | https://dx.doi.org/10.3389/fped.2021.648536 | Wrong population |
| Makkar 2018 | A Hybrid Form of Telemedicine: A Unique Way to Extend Intensive Care Service to Neonates in Medically Underserved Areas | Makkar, A.; McCoy, M.; Hallford, G.; Escobedo, M.; Szyld, E. | 2018 | Telemed J E Health | https://dx.doi.org/10.1089/tmj.2017.0155 | Wrong population |
| Makkar 2020 | Evaluation of Neonatal Services Provided in a Level II NICU Utilizing Hybrid Telemedicine: A Prospective Study | Makkar, A.; McCoy, M.; Hallford, G.; Foulks, A.; Anderson, M.; Milam, J.; Wehrer, M.; Doerfler, E.; Szyld, E. | 2020 | Telemed J E Health | https://dx.doi.org/10.1089/tmj.2018.0262 | Wrong population |
| Marcin 2004 | The use of telemedicine to provide pediatric critical care consultations to pediatric trauma patients admitted to a remote trauma intensive care unit: a preliminary report | Marcin, J. P.; Schepps, D. E.; Page, K. A.; Struve, S. N.; Nagrampa, E.; Dimand, R. J. | 2004 | Pediatr Crit Care Med | NR | Wrong population |
| Marcin 2004 | Use of telemedicine to provide pediatric critical care inpatient consultations to underserved rural Northern California | Marcin, J. P.; Nesbitt, T. S.; Kallas, H. J.; Struve, S. N.; Traugott, C. A.; Dimand, R. J. | 2004 | J Pediatr | NR | Wrong population |
| Mayo 2028 | The TELENEO Trial: A Multicenter Trial of Telemedicine for Advanced Neonatal Resuscitations in Community Hospitals | Mayo, Clinic; Eunice Kennedy Shriver National Institute of Child, Health; Human, Development | 2028 | NR | NR | Wrong population |
| Okada 2020 | Video-call based newborn triage system for local birth centres can be established without major instalment costs using commercially available smartphones | Okada, J.; Hisano, T.; Unno, M.; Tanaka, Y.; Saikusa, M.; Kinoshita, M.; Harada, E.; Iwata, S.; Iwata, O. | 2020 | Sci | https://dx.doi.org/10.1038/s41598-020-64223-w | Wrong population |
| Patel 2023 | The impact of teleneonatology on the Transport Risk Index of Physiologic Stability score for outborn neonates: A prospective, observational study | Patel, R. K.; Kreofsky, B. L.; Hentz, R. C.; Fang, J. L. | 2023 | J Telemed Telecare | https://dx.doi.org/10.1177/1357633X231196334 | Wrong population |
| Schräder 2023 | Telemedicine in neonatal primary care: Influence on staff satisfaction and transfer numbers | Schräder, L.; Schneider, W.; Schunck, K. U.; Girschick, H. | 2023 | Monatsschr. Kinderheilkd. | 10.1007/s00112-023-01813-8 | Wrong population |
| Shimizu 2019 | Usefulness of eICU system in pediatric critical care consultation | Shimizu, N.; Obonai, T.; Saito, O.; Ikeyama, T. | 2019 | Chiba Med. J. | 10.20776/S03035476-95E-2-P21 | Wrong population |
| Silva 2023 | Impact of Telemedicine use on clinical care indicators of pediatric intensive care units: protocol for a cluster randomized clinical trial | Silva, Mmdd; Klever, E. K.; Rocha, J. C. D.; Silva, G. O. L.; Amorim, J. D. R.; Dode, A. D.; Simionato, B. M.; Cunha, L. G. D.; Zaupa, A. P. B.; Krauzer, J. R. M.; Pires, A. A.; Cabral, F. C.; Moreira, T. C.; Constant, Hmrm | 2023 | Crit Care Sci | https://dx.doi.org/10.5935/2965-2774.20230223-en | Wrong population |
| Taylor 2021 | Pediatric trauma telemedicine in a rural state: Lessons learned from a 1-year experience | Taylor, M. A.; Knochel, M. L.; Proctor, S. J.; Brockmeyer, D. L.; Runyon, L. A.; Fenton, S. J.; Russell, K. W. | 2021 | J Pediatr Surg | https://dx.doi.org/10.1016/j.jpedsurg.2020.10.020 | Wrong population |
| Variane 2022 | Protecting brains and saving futures guidelines: A prospective, multicenter, and observational study on the use of telemedicine for neonatal neurocritical care in Brazil | Variane, G. F. T.; Magalhaes, M.; Pietrobom, R. F. R.; Netto, A.; Rodrigues, D. P.; Gasperini, R.; Sant'Anna, G. M. | 2022 | PLoS ONE | https://dx.doi.org/10.1371/journal.pone.0262581 | Wrong population |
| Webb 2013 | Impact of telemedicine on hospital transport, length of stay, and medical outcomes in infants with suspected heart disease: a multicenter study | Webb, C. L.; Waugh, C. L.; Grigsby, J.; Busenbark, D.; Berdusis, K.; Sahn, D. J.; Sable, C. A. | 2013 | J Am Soc Echocardiogr | https://dx.doi.org/10.1016/j.echo.2013.05.018 | Wrong population |
| xj4wyp 2022 | Evaluation of the use of Telemedicine in Pediatric Intensive Care Units: randomized Controlled Trial | xj4wyp, R. B. R. | 2022 | https://trialsearch.who.int/Trial2.aspx?TrialID=RBR-7xj4wyp | NR | Wrong population |
| NR | UPenn Health System uses eICU to lower VAP rates |  | 2009 | Briefings on Patient Safety | NR | Wrong setting |
| NR | A Trial of Two On-Line Interventions for Child Brain Injury | University of, Pittsburgh; Children's Hospital Medical Center, Cincinnati; Centers for Disease, Control; Prevention | NR | NR | NR | Wrong setting |
| AlbertEinsteinHealthcare 2018 | Telemedicine for Improving Outcome in Inner City Patient Population With Hypercapneic Respiratory Failure | Albert Einstein Healthcare, Network | 2018 | NR | NR | Wrong setting |
| Alfraij 2023 | The effect of Telehealth Antimicrobial Stewardship Program (Tele-ASP) on antimicrobial use in a pediatric intensive care unit: Pre- and post-implementation single center study | Alfraij, A.; Abdelmoniem, A.; Elseadawy, M.; Surour, M.; Basuni, M.; Papenburg, J.; Alghounaim, M. | 2023 | J Infect Public Health | https://dx.doi.org/10.1016/j.jiph.2023.06.010 | Wrong setting |
| Bergquist 2017 | THE CONNECT TRIAL: Remotely connecting Traumatic brain injury experts with Those Who Need Them | Bergquist, Thomas F.; Moessner, Anne M.; Brown, Allen W. | 2017 | Brain Injury Professional | NR | Wrong setting |
| DonetskNationalMedical 2008 | Telemedicine Consultation in Trauma and Orthopedic | Donetsk National Medical, University | 2008 | NR | NR | Wrong setting |
| Duchesne 2008 | Impact of telemedicine upon rural trauma care | Duchesne, J. C.; Kyle, A.; Simmons, J.; Islam, S.; Schmieg, R. E., Jr.; Olivier, J.; McSwain, N. E., Jr. | 2008 | J Trauma | https://dx.doi.org/10.1097/TA.0b013e31815dd4c4 | Wrong setting |
| Emanuela 2025 | ICU Cockpit Apps: Interventional Study With First ICU Cockpit Software Applications | Emanuela, Keller; University of, Zurich | 2025 | NR | NR | Wrong setting |
| Forsblom 2021 | Infectious diseases specialist consultation in Staphylococcus lugdunensis bacteremia | Forsblom, E.; Hognas, E.; Syrjanen, J.; Jarvinen, A. | 2021 | PLoS ONE | https://dx.doi.org/10.1371/journal.pone.0258511 | Wrong setting |
| Fusaro 2021 | ICU Telemedicine Implementation and Risk-Adjusted Mortality Differences Between Daytime and Nighttime Coverage | Fusaro, M. V.; Becker, C.; Miller, D.; Hassan, I. F.; Scurlock, C. | 2021 | Chest | https://dx.doi.org/10.1016/j.chest.2020.10.055 | Wrong setting |
| Garingo 2016 | "Tele-rounding" with a remotely controlled mobile robot in the neonatal intensive care unit | Garingo, A.; Friedlich, P.; Chavez, T.; Tesoriero, L.; Patil, S.; Jackson, P.; Seri, I. | 2016 | J Telemed Telecare | https://dx.doi.org/10.1177/1357633X15589478 | Wrong setting |
| Glasgow 2021 | A Comparison of 3D and 2D Telemedicine During Covid 19 | Glasgow, N. H. S. Greater; Clyde; University of, Strathclyde; Glasgow Royal, Infirmary | 2021 | NR | NR | Wrong setting |
| Hofstetter 2023 | Immobilization in Emergency Medical Service - Are CSR and NEXUS-Criteria Considered? A Matched-Pairs Analysis Between Trauma Patients Treated by Onsite EMS Physicians and Patients Treated by Tele-EMS Physicians | Hofstetter, P.; Schroder, H.; Beckers, S. K.; Borgs, C.; Rossaint, R.; Felzen, M. | 2023 | Open access emerg | https://dx.doi.org/10.2147/OAEM.S386650 | Wrong setting |
| Huang 2008 | The availability of telecardiology consultations and transfer patterns from a remote neonatal intensive care unit | Huang, T.; Moon-Grady, A. J.; Traugott, C.; Marcin, J. | 2008 | J Telemed Telecare | https://dx.doi.org/10.1258/jtt.2008.080102 | Wrong setting |
| Ieronimakis 2021 | The Trifecta of Tele-Critical Care: Intrahospital, Operational, and Mass Casualty Applications | Ieronimakis, K. M.; Colombo, C. J.; Valovich, J.; Griffith, M.; Davis, K. L.; Pamplin, J. C. | 2021 | Mil Med | https://dx.doi.org/10.1093/milmed/usaa298 | Wrong setting |
| Increase 2022 | Clinical Decision Support System Based on Non-invasive Tele-monitoring of COVID-19 Patients | Increase, Tech; Hospital Clinico Universitario de, Valladolid; University of, Valladolid; Sanidad de Castilla y, León | 2022 | NR | NR | Wrong setting |
| Ishani 2016 | Telehealth by an Interprofessional Team in Patients With CKD: a Randomized Controlled Trial | Ishani, A.; Christopher, J.; Palmer, D.; Otterness, S.; Clothier, B.; Nugent, S.; Nelson, D.; Rosenberg, M. E. | 2016 | American journal of kidney diseases | 10.1053/j.ajkd.2016.01.018 | Wrong setting |
| Iwashita 2023 | The Experience and the Characteristics of Patients With Tele-ICU Implementation in a Rural Community Hospital | Iwashita, Y.; Ishigame-Kitayama, A.; Yamamoto, A.; Itoh, K.; Takenaka, M.; Morimoto, S.; Yamamoto, Y. | 2023 | Cureus | https://dx.doi.org/10.7759/cureus.41971 | Wrong setting |
| Kennedy 2021 | Perspectives on Telephone and Video Communication in the Intensive Care Unit during COVID-19 | Kennedy, N. R.; Steinberg, A.; Arnold, R. M.; Doshi, A. A.; White, D. B.; DeLair, W.; Nigra, K.; Elmer, J. | 2021 | Ann Am Thorac Soc | https://dx.doi.org/10.1513/AnnalsATS.202006-729OC | Wrong setting |
| Kennedy 2021 | Inpatient Telehealth and Coronavirus Disease 2019 Outcomes: Experiences in Alabama | Kennedy, K. C.; Hearld, K. R.; May, B.; Hall, A. G.; Feldman, S. S.; McKnight, K.; Kraus, A.; Feng, W.; Opoku-Agyeman, W. | 2021 | Telemed Rep | https://dx.doi.org/10.1089/tmr.2021.0004 | Wrong setting |
| Klein 2010 | Management of patients with traumatic intracranial injury in hospitals without neurosurgical service | Klein, Y.; Donchik, V.; Jaffe, D.; Simon, D.; Kessel, B.; Levy, L.; Kashtan, H.; Peleg, K. | 2010 | Journal of Trauma | 10.1097/TA.0b013e3181c99936 | Wrong setting |
| Koruga 2022 | Telemedicine in Neurosurgical Trauma during the COVID-19 Pandemic: A Single-Center Experience | Koruga, N.; Soldo Koruga, A.; Roncevic, R.; Turk, T.; Kopacin, V.; Kretic, D.; Rotim, T.; Roncevic, A. | 2022 | Diagnostics (Basel) | https://dx.doi.org/10.3390/diagnostics12092061 | Wrong setting |
| Latifi 2009 | Initial experiences and outcomes of telepresence in the management of trauma and emergency surgical patients | Latifi, R.; Hadeed, G. J.; Rhee, P.; O'Keeffe, T.; Friese, R. S.; Wynne, J. L.; Ziemba, M. L.; Judkins, D. | 2009 | Am J Surg | https://dx.doi.org/10.1016/j.amjsurg.2009.08.011 | Wrong setting |
| Latifi 2016 | Access to Specialized Care Through Telemedicine in Limited-Resource Country: Initial 1,065 Teleconsultations in Albania | Latifi, R.; Gunn, J. K.; Bakiu, E.; Boci, A.; Dasho, E.; Olldashi, F.; Pipero, P.; Stroster, J. A.; Qesteri, O.; Kucani, J.; Sulo, A.; Oshafi, M.; Osmani, K. L.; Dogjani, A.; Doarn, C. R.; Shatri, Z.; Kociraj, A.; Merrell, R. C. | 2016 | Telemed J E Health | https://dx.doi.org/10.1089/tmj.2016.0050 | Wrong setting |
| Latifi 2018 | Telemedicine for Neurotrauma in Albania: Initial Results from Case Series of 146 Patients | Latifi, R.; Olldashi, F.; Dogjani, A.; Dasho, E.; Boci, A.; El-Menyar, A. | 2018 | World Neurosurg | https://dx.doi.org/10.1016/j.wneu.2018.01.146 | Wrong setting |
| Laudanski 2022 | Deployment of Tele-ICU Respiratory Therapy and the Creation of an eRT Service Line | Laudanski, Krzysztof; Scott, Michael; Huffenberger, Ann Marie; Wain, Justin; Hanson Iii, C. William | 2022 | NEJM Catalyst Innovations in Care Delivery | 10.1056/CAT.21.0239 | Wrong setting |
| LeidenUniversityMedical 2023 | The ICU-recover Box, Using Smart Technology for Monitoring Health Status After ICU Admission | Leiden University Medical, Center | 2023 | NR | NR | Wrong setting |
| Levine 2015 | Tele-intensivists can instruct non-physicians to acquire high-quality ultrasound images | Levine, A. R.; McCurdy, M. T.; Zubrow, M. T.; Papali, A.; Mallemat, H. A.; Verceles, A. C. | 2015 | J Crit Care | https://dx.doi.org/10.1016/j.jcrc.2015.05.030 | Wrong setting |
| Liverpool 2024 | Telehealth Interventions for Cardiac Surgery | Liverpool, Heart; Chest Hospital, N. H. S. Foundation Trust; Liverpool Centre for Cardiovascular, Science | 2024 | NR | NR | Wrong setting |
| Maitre 2021 | Standardized Neurodevelopmental Surveillance of High-risk Infants Using Telehealth: Implementation Study during COVID-19 | Maitre, N. L.; Benninger, K. L.; Neel, M. L.; Haase, J. A.; Pietruszewski, L.; Levengood, K.; Adderley, K.; Batterson, N.; Hague, K.; Lightfoot, M.; Weiss, S.; Lewandowski, D. J.; Larson, H. | 2021 | Pediatr Qual Saf | https://dx.doi.org/10.1097/pq9.0000000000000439 | Wrong setting |
| Marcin 2022 | Measuring the impact of a "Virtual Pediatric Trauma Center" (VPTC) model of care using telemedicine for acutely injured children versus the standard of care: study protocol for a prospective stepped-wedge trial | Marcin, J. P.; Tancredi, D. J.; Galante, J. M.; Rinderknecht, T. N.; Haus, B. M.; Leshikar, H. B.; Zwienenberg, M.; Rosenthal, J. L.; Grether-Jones, K. L.; Hamline, M. Y.; Hoch, J. S.; Kuppermann, N. | 2022 | Trials | https://dx.doi.org/10.1186/s13063-022-06996-1 | Wrong setting |
| Marcin 2024 | Telemedicine vs Telephone Consultations and Medication Prescribing Errors Among Referring Physicians: A Cluster Randomized Crossover Trial | Marcin, J. P.; Lieng, M. K.; Mouzoon, J.; Sauers-Ford, H. S.; Tancredi, D.; Cabri, A.; Pandya, V. A.; Park, A. S.; Kuppermann, N. | 2024 | JAMA netw | https://dx.doi.org/10.1001/jamanetworkopen.2024.0275 | Wrong setting |
| MassachusettsGeneral 2020 | A Randomized Controlled Trial of an Advanced Care Planning Video Decision Support Tool for Patients With End-Stage Liver Disease | Massachusetts General, Hospital | 2020 | NR | NR | Wrong setting |
| Matiello 2021 | Teleneurology-Enabled Determination of Death by Neurologic Criteria After Cardiac Arrest or Severe Neurologic Injury | Matiello, M.; Turner, A. C.; Estrada, J.; Whitney, C. M.; Kitch, B. T.; Lee, P. T.; Girkar, U.; Palacios, R.; Singla, P.; Schwamm, L. | 2021 | Neurology | https://dx.doi.org/10.1212/WNL.0000000000011751 | Wrong setting |
| Mohr 2018 | Telemedicine Use Decreases Rural Emergency Department Length of Stay for Transferred North Dakota Trauma Patients | Mohr, N. M.; Vakkalanka, J. P.; Harland, K. K.; Bell, A.; Skow, B.; Shane, D. M.; Ward, M. M. | 2018 | Telemed J E Health | https://dx.doi.org/10.1089/tmj.2017.0083 | Wrong setting |
| Munusamy 2021 | Telemedicine via Smart Glasses in Critical Care of the Neurosurgical Patient-COVID-19 Pandemic Preparedness and Response in Neurosurgery | Munusamy, T.; Karuppiah, R.; Bahuri, N. F. A.; Sockalingam, S.; Cham, C. Y.; Waran, V. | 2021 | World Neurosurg | https://dx.doi.org/10.1016/j.wneu.2020.09.076 | Wrong setting |
| Nagayoshi 2016 | Clinical Impact of Telemedicine Network System at Rural Hospitals Without On-Site Cardiac Surgery Backup | Nagayoshi, Y.; Oshima, S.; Ogawa, H. | 2016 | Telemed J E Health |  | Wrong setting |
| Nct 2019 | TelePORT Pilot Study | Nct | 2019 | Telehealth-Enhanced Patient-Oriented Recovery Trajectory After Intensive Care Pilot Feasibility Study | NR | Wrong setting |
| Nct 2020 | Improving Family-Centered Pediatric Trauma Care: the Standard of Care Versus the Virtual Pediatric Trauma Center | Nct | 2020 | https://clinicaltrials.gov/show/NCT04469036 | NR | Wrong setting |
| Nct 2021 | Telehealth-Enabled, Real-time Audit and Feedback for Clinician AdHerence (TEACH) | Nct | 2021 | https://clinicaltrials.gov/show/NCT05141396 | NR | Wrong setting |
| Nicholas 2023 | Evaluating Tele-Emergency Care in Costs and Outcomes for Rural Sepsis Patients | Nicholas, M. Mohr; Health, Resources; Services, Administration; University of, Iowa | 2023 | NR | NR | Wrong setting |
| Olldashi 2019 | Telemedicine for Neurotrauma Prevents Unnecessary Transfers: An Update from a Nationwide Program in Albania and Analysis of 590 Patients | Olldashi, F.; Latifi, R.; Parsikia, A.; Boci, A.; Qesteri, O.; Dasho, E.; Bakiu, E. | 2019 | World Neurosurg | https://dx.doi.org/10.1016/j.wneu.2019.04.150 | Wrong setting |
| Pierce 2022 | The Rapid Implementation of Ad Hoc Tele-Critical Care Respiratory Therapy (eRT) Service in the Wake of the COVID-19 Surge | Pierce, M.; Gudowski, S. W.; Roberts, K. J.; Jackominic, A.; Zumstein, K. K.; Shuttleworth, A.; Ho, J.; Susser, P.; Parikh, A.; Chandler, J. M.; Huffenberger, A. M.; Scott, M. J.; Hanson, C. W., 3rd; Laudanski, K. | 2022 | J | https://dx.doi.org/10.3390/jcm11030718 | Wrong setting |
| QueenMaryUniversityof 2022 | Digital Assessment Routing Tool (DART): Pilot Study | Queen Mary University of, London; Optima, Health | 2022 | NR | NR | Wrong setting |
| Roth 2006 | Teleconsultation for cardiac patients: a comparison between nurses and physicians: the SHL experience in Israel | Roth, A.; Rogowski, O.; Yanay, Y.; Kehati, M.; Malov, N.; Golovner, M. | 2006 | Telemed J E Health | NR | Wrong setting |
| Ruesch 2012 | Using nursing expertise and telemedicine to increase nursing collaboration and improve patient outcomes | Ruesch, C.; Mossakowski, J.; Forrest, J.; Hayes, M.; Jahrsdoerfer, M.; Comeau, E.; Singleton, M. | 2012 | Telemed J E Health | https://dx.doi.org/10.1089/tmj.2011.0274 | Wrong setting |
| Savitz 2023 | Evaluation of safety and care outcomes after the introduction of a virtual registered nurse model | Savitz, S. T.; Frederick, R. K.; Sangaralingham, L. R.; Lampman, M. A.; Anderson, S. S.; Habermann, E. B.; Bell, S. J. | 2023 | Health Serv Res | https://dx.doi.org/10.1111/1475-6773.14208 | Wrong setting |
| Silvera 2023 | Draws and windfalls: Comparing patient experiences in inpatient telehealth and non-telehealth acute care units | Silvera, G. A.; Blanchard, E.; Natarajan, V.; Wallace, E.; Stigler, W. S.; Kelly, B.; Scarborough, T.; Feldman, S. S. | 2023 | Patient. Exp. J. | 10.35680/2372-0247.1754 | Wrong setting |
| Takao 2021 | A Smartphone Application as a Telemedicine Tool for Stroke Care Management | Takao, H.; Sakai, K.; Mitsumura, H.; Komatsu, T.; Yuki, I.; Takeshita, K.; Sakuta, K.; Ishibashi, T.; Sakano, T.; Yeh, Y.; Karagiozov, K.; Fisher, M.; Iguchi, Y.; Murayama, Y. | 2021 | Neurol Med Chir (Tokyo) | https://dx.doi.org/10.2176/nmc.oa.2020-0302 | Wrong setting |
| Universityof 2020 | Project to Improve Communication About Serious Illness - Pilot Study | University of, Washington | 2020 | NR | NR | Wrong setting |
| Vespa 2007 | Intensive care unit robotic telepresence facilitates rapid physician response to unstable patients and decreased cost in neurointensive care | Vespa, P. M.; Miller, C.; Hu, X.; Nenov, V.; Buxey, F.; Martin, N. A. | 2007 | Surg Neurol | NR | Wrong setting |
| Vladzymyrskyy 2004 | Our experience with telemedicine in traumatology and orthopedics | Vladzymyrskyy, A. V. | 2004 | Ulus Travma Acil Cerrahi Derg | NR | Wrong setting |
| Watanabe 2023 | An evaluation of the impact of the implementation of the Tele-ICU: a retrospective observational study | Watanabe, T.; Ohsugi, K.; Suminaga, Y.; Somei, M.; Kikuyama, K.; Mori, M.; Maruo, H.; Kono, N.; Kotani, T. | 2023 | J Intensive Care | https://dx.doi.org/10.1186/s40560-023-00657-4 | Wrong setting |
| Wibbenmeyer 2016 | Video-Enhanced Telemedicine Improves the Care of Acutely Injured Burn Patients in a Rural State | Wibbenmeyer, Lucy; Kluesner, Karen; Hongqian, Wu; Eid, Anas; Heard, Jason; Mann, Benjamin; Pauley, Alison; Peek-Asa, Corrine; Wu, Hongqian | 2016 | Journal of Burn Care & Research | 10.1097/BCR.0000000000000268 | Wrong setting |
| Yager 2014 | Reliability of circulatory and neurologic examination by telemedicine in a pediatric intensive care unit | Yager, P. H.; Clark, M. E.; Dapul, H. R.; Murphy, S.; Zheng, H.; Noviski, N. | 2014 | J Pediatr | https://dx.doi.org/10.1016/j.jpeds.2014.07.002 | Wrong setting |
| Yurkiewicz 2012 | Outcomes from a US military neurology and traumatic brain injury telemedicine program | Yurkiewicz, I. R.; Lappan, C. M.; Neely, E. T.; Hesselbrock, R. R.; Girard, P. D.; Alphonso, A. L.; Tsao, J. W. | 2012 | Neurology | https://dx.doi.org/10.1212/WNL.0b013e31826aac33 | Wrong setting |
| NR | Telemedicine's Role in the Intensive Care Setting | NR | 2019 | AACN Bold Voices | NR | Wrong study design |
| NR | Telemedicine Program Reduces ICU Interhospital Transfers | NR | 2018 | AACN Bold Voices | NR | Wrong study design |
| NR | Tele-ICU Takes Flight at Air Force Base: Offered in partnership with the VA, the technology can fill gaps in medical coverage and provide a second set of eyes for ICUs | NR | 2017 | AACN Bold Voices | NR | Wrong study design |
| NR | First $50,000 AACN Impact Research Grant: Tele-ICUs |  | 2011 | AACN Bold Voices | NR | Wrong study design |
| NR | CCRN-E initial certification validates tele-ICU knowledge |  | 2011 | AACN Bold Voices | NR | Wrong study design |
| NR | Tele-ICUs offer potential to improve patient care |  | 2010 | AACN Bold Voices | NR | Wrong study design |
| Amkreutz 2020 | Medication safety in a German telemedicine centre: Implementation of a telepharmaceutical expert consultation in addition to existing tele-intensive care unit services | Amkreutz, J.; Lenssen, R.; Marx, G.; Deisz, R.; Eisert, A. | 2020 | J Telemed Telecare | https://dx.doi.org/10.1177/1357633X18799796 | Wrong study design |
| Belcher 2023 | Implementation and Impact of Critical Care Pharmacist Addition to a Telecritical Care Network | Belcher, R. M.; Blair, A.; Chauv, S.; Hoang, Q.; Hickman, A. W.; Peng, M.; Baldwin, M.; Koch, L.; Nguyen, M.; Guidry, D.; Fontaine, G. V. | 2023 | Crit | https://dx.doi.org/10.1097/CCE.0000000000000839 | Wrong study design |
| Boots 2012 | The tyranny of distance: telemedicine for the critically ill in rural Australia | Boots, R. J.; Singh, S. J.; Lipman, J. | 2012 | Anaesth Intensive Care | NR | Wrong study design |
| CapitalMedical 2021 | Management of PADIS in Emergency Intensive Care Unit | Capital Medical, University; Beijing Tiantan, Hospital; The First Affiliated Hospital of Anhui Medical, University; The First Affiliated Hospital of Dalian Medical, University; Fudan, University; Affiliated hospital of Guilin medical university, China; The Second Affiliated Hospital of Hainan Medical, University; The First Hospital of Hebei Medical, University; The First Hospital of Jilin, University; Southwest Hospital, China; General hospital of shenyang military, command; Beijing Anzhen, Hospital; Mianyang Central, Hospital; Tianjin Medical University General, Hospital; The First Affiliated Hospital of Zhengzhou, University; The Jiangxi Provincial People's, Hospital | 2021 | NR | NR | Wrong study design |
| Degaspari 2012 | An alternative approach to 24/7 ICU coverage. Can tele-ICU monitoring fill the intensivist shortage? | Degaspari, J. | 2012 | Healthc Inform | NR | Wrong study design |
| Dohmen 2021 | Measurable patient benefit through digital healthcare networks for COVID-19 patients requiring intensive care in the Virtual Hospital NRW | Dohmen, S.; Benstoem, C.; Lemmen, S. W.; Eisert, A.; Zarbock, A.; Marx, G. | 2021 | Anasthesiol. Intensivmed. | 10.19224/ai2021.431 | Wrong study design |
| Fuzaylov 2013 | Use of telemedicine to improve burn care in Ukraine | Fuzaylov, G.; Knittel, J.; Driscoll, D. N. | 2013 | J Burn Care Res | https://dx.doi.org/10.1097/BCR.0b013e3182779b40 | Wrong study design |
| Glasgow 2023 | Clinical Trial of 3D Telemedicine | Glasgow, N. H. S. Greater; Clyde; University of, Strathclyde; Microsoft, Corporation | 2023 | NR | NR | Wrong study design |
| Golembeski 2012 | Perceptions of the care experience in critical care units enhanced by a tele-ICU | Golembeski, S.; Willmitch, B.; Kim, S. S. | 2012 | AACN Adv Crit Care | https://dx.doi.org/10.1097/NCI.0b013e31825ed8bb | Wrong study design |
| Griffiths 2022 | Characterization of dayshift tele-ICU pharmacist activities | Griffiths, C. L.; Kosmisky, D. E.; Everhart, S. S. | 2022 | J Telemed Telecare | https://dx.doi.org/10.1177/1357633X20913712 | Wrong study design |
| Khunlertkit 2013 | Contributions of tele-intensive care unit (Tele-ICU) technology to quality of care and patient safety | Khunlertkit, A.; Carayon, P. | 2013 | J Crit Care | https://dx.doi.org/10.1016/j.jcrc.2012.10.005 | Wrong study design |
| Kleinpell 2016 | Assessing the Impact of Telemedicine on Nursing Care in Intensive Care Units | Kleinpell, R.; Barden, C.; Rincon, T.; McCarthy, M.; Zapatochny Rufo, R. J. | 2016 | Am J Crit Care | https://dx.doi.org/10.4037/ajcc2016808 | Wrong study design |
| Koeck 2023 | Comparison of Drug-Related Problems in COVID-19 and Non-COVID-19 Patients Provided by a German Telepharmacy Service for Rural Intensive Care Units | Koeck, J. A.; Dohmen, S. M.; Marx, G.; Eisert, A. | 2023 | J | https://dx.doi.org/10.3390/jcm12144739 | Wrong study design |
| Kovacevic 2019 | Impact of weekly case-based tele-education on quality of care in a limited resource medical intensive care unit | Kovacevic, P.; Dragic, S.; Kovacevic, T.; Momcicevic, D.; Festic, E.; Kashyap, R.; Niven, A. S.; Dong, Y.; Gajic, O. | 2019 | Crit Care | https://dx.doi.org/10.1186/s13054-019-2494-6 | Wrong study design |
| Krouss 2020 | Rapid Implementation of Telecritical Care Support During a Pandemic: Lessons Learned During the Coronavirus Disease 2020 Surge in New York City | Krouss, M.; Allison, M. G.; Rios, S.; Bringardner, B. D.; Langston, M. D.; Sokol, S. I.; McCurdy, M. T. | 2020 | Crit | https://dx.doi.org/10.1097/CCE.0000000000000271 | Wrong study design |
| Lieder 2000 | Telemedicine company brings ICU patients to the physician | Lieder, T. R. | 2000 | Am J Health-Syst Pharm | NR | Wrong study design |
| Martinez 2017 | Evaluation and Treatment of Mild Traumatic Brain Injury Through the Implementation of Clinical Video Telehealth: Provider Perspectives From the Veterans Health Administration | Martinez, R. N.; Hogan, T. P.; Lones, K.; Balbale, S.; Scholten, J.; Bidelspach, D.; Musson, N.; Smith, B. M. | 2017 | Pm R | https://dx.doi.org/10.1016/j.pmrj.2016.07.002 | Wrong study design |
| Munoz 2012 | Telemedicine in pediatric cardiac critical care | Munoz, R. A.; Burbano, N. H.; Motoa, M. V.; Santiago, G.; Klevemann, M.; Casilli, J. | 2012 | Telemed J E Health | https://dx.doi.org/10.1089/tmj.2011.0090 | Wrong study design |
| Nayar 2021 | Novel multidisciplinary hub-and-spoke tertiary service for the management of severe acute pancreatitis | Nayar, M. K.; Bekkali, N. L. H.; Bourne, D.; Young, S.; Leeds, J. S.; Oppong, K. W.; Logue, J. L.; Sen, G.; French, J. J.; Scott, J.; Cressey, D.; Pandanaboyana, S.; Charnley, R. M. | 2021 | BMJ Open Gastroenterol | https://dx.doi.org/10.1136/bmjgast-2020-000501 | Wrong study design |
| Palacios 2023 | Predictive Models to Optimize Resources in Tele-Critical Care in Distributed Hospital Networks | Palacios, R.; SÃ¡nchez-Ãºbeda, E.; Panos, R.; Argaw, P.; Shahrawat, M.; Zhang, D. D.; Zhang, A.; Seiver, A.; Badawi, O.; Gupta, A. | 2023 | Telehealth. Med. Today. | 10.30953/tmt.v8.408 | Wrong study design |
| Pilosof 2021 | Inpatient Telemedicine and New Models of Care during COVID-19: Hospital Design Strategies to Enhance Patient and Staff Safety | Pilosof, N. P.; Barrett, M.; Oborn, E.; Barkai, G.; Pessach, I. M.; Zimlichman, E. | 2021 | Int J Environ Res Public Health | https://dx.doi.org/10.3390/ijerph18168391 | Wrong study design |
| Starr 2024 | Safety and timeliness of telemedicine initiation of continuous kidney replacement therapy | Starr, M. C.; Altemose, K.; Parsley, J.; Cater, D. T.; Hains, D. S.; Soranno, D. E. | 2024 | Pediatr Nephrol | https://dx.doi.org/10.1007/s00467-023-06036-3 | Wrong study design |
| Umin 2017 | Infrastructure of Tele-Intensive Care Unit | Umin | 2017 | https://trialsearch.who.int/Trial2.aspx?TrialID=JPRN-UMIN000025516 | NR | Wrong study design |
| Universityof 2022 | Remote By Default 2: Optimising the Remote-by-default Model in the United Kingdom (UK) General Practice | University of, Oxford; University of, Plymouth; Nuffield, Trust | 2022 | NR | NR | Wrong study design |
| vanderVoort 2016 | Telemedicine in a Dutch intensive care unit: A descriptive study of the first results | van der Voort, P. H.; de Metz, J.; Wester, J. P.; van Stijn, I.; Feijen, H. M.; Balzereit, A.; Rijkenberg, S.; Obster, R.; Bosman, R. J. | 2016 | J Telemed Telecare | https://dx.doi.org/10.1177/1357633X15590751 | Wrong study design |
| Weiss 2021 | [Avoiding Long-term Impairment in Critical Care Using Telemedicine: The ERIC Example] | Weiss, B.; Paul, N.; Kraufmann, B.; Spies, C. D. | 2021 | Anasthesiol Intensivmed Notfallmed Schmerzther | https://dx.doi.org/10.1055/a-1130-4996 | Wrong study design |
| ZapatochnyRufo 2007 | Virtual ICUs: foundations for healthier environments | Zapatochny Rufo, R. J. | 2007 | Nurs Manage | DOI | Wrong study design |
