# Supplementary material for: Telemedicine in adult intensive care: A systematic review of patient-relevant outcomes and methodological considerations
Source: PLOS Digit Health. 2025 Dec 15;4(12):e0001126. doi: 10.1371/journal.pdig.0001126 (PMC12704867; doi:10.1371/journal.pdig.0001126)
Supplement: S2 Table — (DOCX) [file pdig.0001126.s005.docx]

**Table 2: Ongoing studies and studies held in ‘awaiting classification’ of the primary search.**

| Study ID | Title | Authors | Published Year | Journal | DOI | Status | Reason for awaiting classification |
| --- | --- | --- | --- | --- | --- | --- | --- |
| McLeroy 2020 | Implementation of Tele-Critical Care at General Leonard Wood Army Community Hospital | McLeroy, R. D.; Ingersoll, J.; Nielsen, P.; Pamplin, J. | 2020 | Mil Med | https://dx.doi.org/10.1093/milmed/usz147 | Awaiting classification | control group conditions unclear |
| Kalb 2014 | A multicenter population-based effectiveness study of teleintensive care unit-directed ventilator rounds demonstrating improved adherence to a protective lung strategy, decreased ventilator duration, and decreased intensive care unit mortality | Kalb, T.; Raikhelkar, J.; Meyer, S.; Ntimba, F.; Thuli, J.; Gorman, M. J.; Kopec, I.; Scurlock, C. | 2014 | J Crit Care | https://dx.doi.org/10.1016/j.jcrc.2014.02.017 | Awaiting classification | control group conditions unclear, missing patient data |
| Al-Omari 2020 | A Multicenter Case-Historical Control Study on Short-Term Outcomes of Tele-Intensive Care Unit | Al-Omari, A.; Al Mutair, A.; Al Ammary, M.; Aljamaan, F. | 2020 | Telemed J E Health | https://dx.doi.org/10.1089/tmj.2019.0042 | Awaiting classification | missing details regarding the techinical TM system |
| Becker 2020 | Effects of Telemedicine ICU Intervention on Care Standardization and Patient Outcomes: An Observational Study | Becker, C. D.; Fusaro, M. V.; Al Aseri, Z.; Millerman, K.; Scurlock, C. | 2020 | Crit | https://dx.doi.org/10.1097/CCE.0000000000000165 | Awaiting classification | missing details regarding the techinical TM system |
| Essay 2023 | Managed critical care: impact of remote decision-making on patient outcomes | Essay, P.; Zhang, T.; Mosier, J.; Subbian, V. | 2023 | Am J Manag Care | https://dx.doi.org/10.37765/ajmc.2023.89400 | Awaiting classification | missing details regarding the techinical TM system |
| Graves 2024 | Telemedicine critical care availability and outcomes among mechanically ventilated patients | Graves, J. M.; Krings, J. G.; Buss, J. L.; Kallogjeri, D.; Ofoma, U. R. | 2024 | J Crit Care | https://dx.doi.org/10.1016/j.jcrc.2024.154782 | Awaiting classification | missing details regarding the techinical TM system |
| Gupta 2014 | eICU reduces mortality in STEMI patients in resource-limited areas | Gupta, S.; Dewan, S.; Kaushal, A.; Seth, A.; Narula, J.; Varma, A. | 2014 | Glob Heart | https://dx.doi.org/10.1016/j.gheart.2014.07.006 | Awaiting classification | missing details regarding the techinical TM system |
| Hawkins 2016 | ICU Telemedicine Comanagement Methods and Length of Stay | Hawkins, H. A.; Lilly, C. M.; Kaster, D. A.; Groves, R. H., Jr.; Khurana, H. | 2016 | Chest | https://dx.doi.org/10.1016/j.chest.2016.03.030 | Awaiting classification | missing details regarding the techinical TM system |
| Ofoma 2022 | Outcomes of in-hospital cardiac arrest among hospitals with and without telemedicine critical care | Ofoma, U. R.; Drewry, A. M.; Maddox, T. M.; Boyle, W.; Deych, E.; Kollef, M.; Girotra, S.; Joynt Maddox, K. E. | 2022 | Resuscitation | https://dx.doi.org/10.1016/j.resuscitation.2022.06.008 | Awaiting classification | missing details regarding the techinical TM system, missing patient data information |
| Zawada 2009 | Impact of an intensive care unit telemedicine program on a rural health care system | Zawada, E. T., Jr.; Herr, P.; Larson, D.; Fromm, R.; Kapaska, D.; Erickson, D. | 2009 | Postgrad Med | https://dx.doi.org/10.3810/pgm.2009.05.2016 | Awaiting classification | missing details regarding the techinical TM system, missing patient data information |
| Dudas 2023 | Cost Effective Virtual Intensive Care Unit Expanding Capabilities in Critical Access Hospitals | Dudas, L. M.; Bardes, J. M.; Wagner, A. K.; White, T.; Wilson, A. M. | 2023 | Am Surg | https://dx.doi.org/10.1177/00031348211062653 | Awaiting classification | missing patients data |
| Steinman 2015 | Impact of telemedicine in hospital culture and its consequences on quality of care and safety | Steinman, M.; Morbeck, R. A.; Pires, P. V.; Abreu Filho, C. A.; Andrade, A. H.; Terra, J. C.; Teixeira Junior, J. C.; Kanamura, A. H. | 2015 | Einstein | https://dx.doi.org/10.1590/S1679-45082015GS2893 | Awaiting classification | mixed population |
| Jprn 2016 | Evaluation of efficacy of telemedicine ICU (eICU) first in Japan | Hiroshi, Otake | 2016 | Evaluation of efficacy of telemedicine ICU (eICU) first in Japan - Evaluation of efficacy of telemedicine ICU (eICU) first in Japan | Jprn, Umin | Awaiting classification | ongoing study; patients' age unclear |
| Kohl 2012 | The effect of ICU telemedicine on mortality and length of stay | Kohl, B. A.; Fortino-Mullen, M.; Praestgaard, A.; Hanson, C. W.; Dimartino, J.; Ochroch, E. A. | 2012 | J Telemed Telecare | https://dx.doi.org/10.1258/jtt.2012.120208 | Awaiting classification | patients' age unclear |
| Zawada 2006 | Prognostic outcomes after the initiation of an electronic telemedicine intensive care unit (eICU) in a rural health system | Zawada, E. T., Jr.; Kapaska, D.; Herr, P.; Aaronson, M.; Bennett, J.; Hurley, B.; Bishop, D.; Dagher, H.; Kovaleski, D.; Melanson, T.; Burdge, K.; Johnson, T. | 2006 | S D Med |  | Awaiting classification | patients' age unclear |
| Grissom 2023 | Implementation of coordinated spontaneous awakening and breathing trials using telehealth-enabled, real-time audit and feedback for clinician adherence (TEACH): a type II hybrid effectiveness-implementation cluster-randomized trial | Grissom, C. K.; Holubkov, R.; Carpenter, L.; Hanna, B.; Jacobs, J. R.; Jones, C.; Knighton, A. J.; Leither, L.; Lisonbee, D.; Peltan, I. D.; Winberg, C.; Wolfe, D.; Srivastava, R. | 2023 | Implement Sci | https://dx.doi.org/10.1186/s13012-023-01303-1 | Ongoing study | NA |
| HospitalIsraelitaAlbert 2026 | Evaluation of the Clinical Impact of Different Telemedicine Practices in Intensive Care Units | Nct | 2023 | https://clinicaltrials.gov/ct2/show/NCT05960994 |  | Ongoing study | NA |
| HospitalIsraelitaAlbert 2026 | Evaluation of the Clinical Impact of Different Telemedicine Practices in Intensive Care Units | Hospital Israelita Albert, Einstein; Ministry of Health, Brazil | 2026 |  |  | Ongoing study | NA |
| Nct 2022 | A Multifaceted Telemedicine-Based Intervention to Improve Outcomes of Cancer Patients Admitted to the ICU | Nct | 2022 | https://clinicaltrials.gov/show/NCT05423795 |  | Ongoing study | NA |
