# Supplementary material for: Telemedicine in adult intensive care: A systematic review of patient-relevant outcomes and methodological considerations
Source: PLOS Digit Health. 2025 Dec 15;4(12):e0001126. doi: 10.1371/journal.pdig.0001126 (PMC12704867; doi:10.1371/journal.pdig.0001126)
Supplement: S3 Table — (DOCX) [file pdig.0001126.s006.docx]

**Table 3: Excluded studies with reasons of update RCT searches.**

| Study ID | Title | Authors | Published Year | Journal | DOI | Reason for exclusion |
| --- | --- | --- | --- | --- | --- | --- |
| Search date: 26.10.2024 | | | | | | |
| Nct 2024 | UPTAKE - Virtual Care: virtual Home Hospital With Remote Monitoring to Reduce Acute Care Hospitalization | Nct | 2024 | https://clinicaltrials.gov/ct2/show/NCT06537453 | NA | Wrong setting |
| Pamplin 2024 | Randomized Controlled Trial of Telementoring During Resource-Limited Patient Care Simulation Improves Caregiver Performance and Patient Survival | Pamplin, J. C.; Veazey, S. R.; Barczak, S.; Fonda, S. J.; Serio-Melvin, M. L.; Ross, K. S.; Colombo, C. J. | 2024 | Crit | https://dx.doi.org/10.1097/CCE.0000000000001090 | Wrong setting |
| Search date: 29.09.2025 | | | | | | |
| DRKS00035294 | REAching the Calorie goal with Telemedicine in the ICU. A randomized controlled study | Drks | 2025 | https://trialsearch.who.int/Trial2.aspx?TrialID=DRKS00035294 | NA | Wrong intervention |
| jRCT1051240286 | Development of Remote Engagement and Assistance in Modern ICUs | jRct, Jprn | 2025 | https://trialsearch.who.int/Trial2.aspx?TrialID=JPRN-jRCT1051240286 | NA | Wrong intervention |
| Limotai 2025 | Efficacy of delivery of care with Tele-continuous EEG in critically ill patients: a multicenter randomized controlled trial (Tele-cRCT study) study | Limotai, C.  Jirasakuldej, S.  Wongwiangiunt, S.  Tumnark, T.  Suwanpakdee, P.  Wangponpattanasiri, K.  Rakchue, P.  Tungkasereerak, C.  Pleumpanupatand, P.  Tansuhaj, P.  Ekkachon, P.  Kittipanprayoon, S.  Kerddonfag, A.  Pobsuk, T.  Pattanateepapon, A.  Phanthumchinda, K.  Suwanwela, N. C.  Thaipisuttikul, I.  Boonyapisit, K.  Ingsathit, A.  Pattanaprateep, O.  Attia, J.  McKay, G. J.  Rossetti, A. O.  Thakkinstian, A.  Rukrung, C.  Kangsananont, P.  Mokkaew, J.  Phayaph, N.  Pukpraman, S.  Ritrhathon, W.; Jarungjitapinan, Y.; Pinpradab, J.; Khamhoi, N.; Nookaew, M.; Chauywang, P.; Rojdmapitayakorn, P.; Sribussara, P.; Tinroongroj, W.; Teeratantikanon, W.; Chongsuvivatwong, T.; Viratyaporn, W.; Jantararotai, W.; Panyawattanakit, K.; Rujirarongrueng, N.; Damthong, P.; Udom, P.; Siengsuwan, M.; Phonprasori, P.; Wanmuang, K.; Unwanatham, N.; Rattanasiri, S.; Thadanipon, K.; Noivong, P.; Pitipanyakul, S.; Rattanachaisit, W.; Muangthong, W.; Wittayawisawasakul, R.; Deerassamee, S.; Ruayruen, W.; Homgrunjarut, S.; Deerassamee, S.; Ledprased, Y.; Pankong, M.; Rattanayuvakorn, P. | 2025 | Crit Care | https://dx.doi.org/10.1186/s13054-024-05246-x | Wrong intervention |
| King 2025 | Effect of telemedicine support for intraoperative anaesthesia care on postoperative outcomes: the TECTONICS randomised clinical trial | King, C. R.; Fritz, B. A.; Gregory, S. H.; Budelier, T. P.; BenAbdallah, A.; Kronzer, A.; Helsten, D. L.; Torres, B.; McKinnon, S. L.; Tripathi, S.; Abdelhack, M.; Goswami, S.; Montes de Oca, A.; Mehta, D.; Valdez, M. A.; Karanikolas, E.; Higo, O.; Kerby, P.; Henrichs, B.; Wildes, T. S.; Politi, M. C.; Abraham, J.; Avidan, M. S.; Kannampallil, T. | 2025 | British Journal of Anaesthesia | https://dx.doi.org/10.1016/j.bja.2024.11.017 | Wrong population |
