# Supplementary material for: Telemedicine in adult intensive care: A systematic review of patient-relevant outcomes and methodological considerations
Source: PLOS Digit Health. 2025 Dec 15;4(12):e0001126. doi: 10.1371/journal.pdig.0001126 (PMC12704867; doi:10.1371/journal.pdig.0001126)
Supplement: S4 Table — (DOCX) [file pdig.0001126.s007.docx]

**Table 4: Telemedicine characteristics of included studies.**

| Study ID | Type of communication | Mode of communication | Availability of telemedicine provider(s) | Delegations grade | Telemedicine expertise/ training provider | Telemedicine expertise/ training recipient |
| --- | --- | --- | --- | --- | --- | --- |
| Boyle 2023 | "high-tech" audio-video (e.g. specific communication technology for telemedical purpose, i.e. remote-controlled camera in the patients room); Electronic health record with automated data transfer | Emergency contact (without further information on interview); Structured interview during contact on demand ; Structured interview during daily rounding (e.g. routine); Other: 24h-continous monitoring | 24/7 | NR | NR | any other ICU staff (no further information on training) |
| Breslow 2004 | "high-tech" audio-video (e.g. specific communication technology for telemedical purpose, i.e. remote-controlled camera in the patients room); Electronic health record with automated data transfer | Emergency contact (without further information on interview); Daily rounding (without further information on interview) | 19 hrs/day (noon to 7 am) | Consultation; Therapeutic decision making | tele-ICU expert | ICU staff with tele-ICU training |
| Collins 2017 | "high-tech" audio-video (e.g. specific communication technology for telemedical purpose, i.e. remote-controlled camera in the patients room); Other: EHR without further information | Emergency contact (without further information on interview); Contact on demand (without further information on interview) | two ICU registered nurses during the day (7am to 7pm) and a physician intensivist and nurse at night (7pm to7am), from 7 am to 7 pm an in-house SCCS nurse practitioner and intensivist were responsible for managing the patients | Consultation; Therapeutic decision making | any other ICU staff (no further information on training) | ICU staff with tele-ICU training |
| Davis 2017 | "high-tech" audio-video (e.g. specific communication technology for telemedical purpose, i.e. remote-controlled camera in the patients room); Electronic health record without automated data transfer (e.g. screen sharing, email, fax) | Emergency contact (without further information on interview); Contact on demand (without further information on interview); Daily rounding (without further information on interview) | 24/7 | Consultation; Therapeutic decision making | ICU staff with tele-ICU training | NR |
| Forni 2010 | Telephone; "low-tech" audio-video (e.g. laptop, mobile); Electronic health record with automated data transfer | Structured interview during contact on demand ; Structured interview during daily rounding (e.g. routine) | 24/7 | Therapeutic decision making | ICU staff with tele-ICU training | NR |
| Fortis 2014 | "high-tech" audio-video (e.g. specific communication technology for telemedical purpose, i.e. remote-controlled camera in the patients room); Electronic health record with automated data transfer | NR | 24/7 | Consultation; Therapeutic decision making | any other ICU staff (no further information on training) | ICU staff with tele-ICU training |
| Fortis 2018 ^a^ | "high-tech" audio-video (e.g. specific communication technology for telemedical purpose, i.e. remote-controlled camera in the patients room); Electronic health record with automated data transfer | Unstructured interview during contact on demand | During the implementation period, the Minneapolis center moved  toward 24/7 coverage by at least one board certified critical care physician. At Cincinnati center, a  board certified intensivist was present 24/7 except 7 am to 4 pm Monday to Friday. | Consultation | any other ICU staff (no further information on training) | NR |
| Kahn 2016 | "high-tech" audio-video (e.g. specific communication technology for telemedical purpose, i.e. remote-controlled camera in the patients room); Electronic health record with automated data transfer | Within this framework programs may have varied with respect to the make-up of the support center team, the hours the support | Within this framework programs may have varied with respect to the make-up of the support center team, the hours the support center was staffed, and the specific activities of the support  center clinicians. | NR | NR | NR |
| Lilly 2011 | "high-tech" audio-video (e.g. specific communication technology for telemedical purpose, i.e. remote-controlled camera in the patients room); Electronic health record with automated data transfer | Structured interview during emergency contact; Structured interview during daily rounding (e.g. routine); Other: The tele-ICU team serially reviewed the care of individual patients, performed real-time audits of best practice adherence, performed workstation-assisted care plan reviews for patients admitted at night | 24/7 | Consultation; Therapeutic decision making | ICU staff with tele-ICU training | ICU staff with tele-ICU training |
| Lilly 2014 | "low-tech" audio-video (e.g. laptop, mobile); Electronic health record without automated data transfer (e.g. screen sharing, email, fax) | Changes in process of care delivery, ICU admission procedures, rounding and governance structure, communication among caregivers, how performance information was used, how care was documented, how technical support was provided, and other factors varied among implementations. | changes in process of care delivery, ICU  admission procedures, rounding and governance structure, communication among caregivers, how performance information was used, how care was documented, how technical support was provided, and other factors varied among implementations. | changes in process of care delivery, ICU  admission procedures, rounding and governance structure, communication among caregivers, how performance information was used, how care was documented, how technical support was provided, and other factors varied among implementations | NR | NR |
| Lilly 2017 | "high-tech" audio-video (e.g. specific communication technology for telemedical purpose, i.e. remote-controlled camera in the patients room); Electronic health record with automated data transfer | Emergency contact (without further information on interview); Contact on demand (without further information on interview); Daily rounding (without further information on interview) | 27/7 | admission procedures, rounding and governance structure, communication among caregivers, how performance information was used, how care was documented, how technical support was provided, and other factors varied among implementations" | NR | NR |
| Marx 2022 | "high-tech" audio-video (e.g. specific communication technology for telemedical purpose, i.e. remote-controlled camera in the patients room); Electronic health record with automated data transfer | Emergency contact (without further information on interview); Structured interview during contact on demand ; Structured interview during daily rounding (e.g. routine) | Consultants for intensive care medicine participated in key care processes 24/7, whereas infectious disease specialists were available once weekly and on demand,  including participation in rounds, additional expert teleconsultations, emergency consultations, and audits of clinical patient data. | Consultation | tele-ICU expert | NR |
| McCambridge 2010 | "high-tech" audio-video (e.g. specific communication technology for telemedical purpose, i.e. remote-controlled camera in the patients room); Electronic health record with automated data transfer | Emergency contact (without further information on interview); Contact on demand (without further information on interview); Daily rounding (without further information on interview) | From 7 pm to 7 am | NR | any other ICU staff (no further information on training) | any other ICU staff (no further information on training) |
| Morrison 2010 | "high-tech" audio-video (e.g. specific communication technology for telemedical purpose, i.e. remote-controlled camera in the patients room); Electronic health record with automated data transfer | Daily rounding (without further information on interview) | 24/7 | Consultation; Therapeutic decision making | any other ICU staff (no further information on training) | any other ICU staff (no further information on training) |
| Nassar 2014 ^a^ | "high-tech" audio-video (e.g. specific communication technology for telemedical purpose, i.e. remote-controlled camera in the patients room); Electronic health record with automated data transfer | Emergency contact (without further information on interview); Contact on demand (without further information on interview) | 21 hours per day, 7 days per week | Consultation; Therapeutic decision making (in 6 ICUs, TM staff authorized to monitor patients and make interventions.  In 2 ICUs, TM staff could intervene only when explicitly requested. In emergency situations, TM staff were preauthorized to intervene at all sites) | NR | NR |
| O’Shea 2022 ^a^ | "high-tech" audio-video (e.g. specific communication technology for telemedical purpose, i.e. remote-controlled camera in the patients room); Electronic health record with automated data transfer | Emergency contact (without further information on interview); Contact on demand (without further information on interview); Daily rounding (without further information on interview) | NR | Consultation | NR | NR |
| Panlaqui 2017 | "high-tech" audio-video (e.g. specific communication technology for telemedical purpose, i.e. remote-controlled camera in the patients room) | Daily rounding (without further information on interview) | NR | Consultation | any other ICU staff (no further information on training) | any other ICU staff (no further information on training) |
| Pannu 2017 | "low-tech" audio-video (e.g. laptop, mobile); Electronic health record with automated data transfer | NR | 24/7 | The telemedicine center acts as a 24/7 surveillance unit. Comanagement | NR | NR |
| Pereira 2024 | "high-tech" audio-video (e.g. specific communication technology for telemedical purpose, i.e. remote-controlled camera in the patients room) | Emergency contact (without further information on interview); Contact on demand (without further information on interview); Structured interview during daily rounding (e.g. routine) | Monday to Friday | Consultation | ICU staff with tele-ICU training | NR |
| Rosenfeld 2000 | "high-tech" audio-video (e.g. specific communication technology for telemedical purpose, i.e. remote-controlled camera in the patients room); Electronic health record without automated data transfer (e.g. screen sharing, email, fax) | Emergency contact (without further information on interview); Contact on demand (without further information on interview); Daily rounding (without further information on interview) | 24/7 | Consultation; Therapeutic decision making | NR | ICU staff with tele-ICU training |
| Sadaka 2013 | "high-tech" audio-video (e.g. specific communication technology for telemedical purpose, i.e. remote-controlled camera in the patients room); Electronic health record with automated data transfer | Emergency contact (without further information on interview); Contact on demand (without further information on interview) | 24/7 | Consultation; Therapeutic decision making | any other ICU staff (no further information on training) | any other ICU staff (no further information on training) |
| Spies 2023 | "high-tech" audio-video (e.g. specific communication technology for telemedical purpose, i.e. remote-controlled camera in the patients room); Electronic health record with automated data transfer | Emergency contact (without further information on interview); Contact on demand (without further information on interview); Structured interview during daily rounding (e.g. routine) | 24/7 | Consultation | tele-ICU expert | ICU staff with tele-ICU training |
| Thomas 2009 | "high-tech" audio-video (e.g. specific communication technology for telemedical purpose, i.e. remote-controlled camera in the patients room) | Emergency contact (without further information on interview); Daily rounding (without further information on interview) | Two intensivists: from noon to 7 am Monday through Friday and 24 hours a day on Saturday and Sunday  4 nurses and administrative technicians: 24/7 | Consultation; Therapeutic decision making | any other ICU staff (no further information on training) | any other ICU staff (no further information on training) |
| Udeh 2022 | Electronic health record with automated data transfer | Emergency contact (without further information on interview); Contact on demand (without further information on interview) | overnight support | Consultation; Therapeutic decision making | NR | NR |
| VanGent 2018 | "high-tech" audio-video (e.g. specific communication technology for telemedical purpose, i.e. remote-controlled camera in the patients room); Electronic health record with automated data transfer | Contact on demand (without further information on interview) | The NMCSD teleintensivist covered the NHCP ICU when no local intensivist was available. TCC call periods ranged from days to months. | Consultation | NR | NR |
| Willmitch 2012 | "high-tech" audio-video (e.g. specific communication technology for telemedical purpose, i.e. remote-controlled camera in the patients room) | NR | 24/7 | Consultation; Therapeutic decision making | NR | NR |

**Abbreviations:** Intensive care unit (ICU), Naval Hospital Camp Pendleton (NHCP), Naval Medical Center San Diego (NMCSD), not reported (NR), surgical critical care service (SCCS), tele-critical care (TCC), telemedicine (TM).

**Footnotes:**

**^a^** Studies used the same population pool for analyses.
