# Supplementary material for: Telemedicine in adult intensive care: A systematic review of patient-relevant outcomes and methodological considerations
Source: PLOS Digit Health. 2025 Dec 15;4(12):e0001126. doi: 10.1371/journal.pdig.0001126 (PMC12704867; doi:10.1371/journal.pdig.0001126)
Supplement: S5 Table — (DOCX) [file pdig.0001126.s008.docx]

**Table 5: Mean and median age per group of all included studies.**

| Study ID | Group 1: Age (mean) | Group 1: Age (median) | Group 2: Age (mean) | Group 2: Age (median) | Group 3: Age (mean) | Group 3: Age (median) | Group 4: Age (mean) | Group 4: Age (median) |
| --- | --- | --- | --- | --- | --- | --- | --- | --- |
| Boyle 2023 | NR | Post-TCC: 62 | NR | Pre-TCC: 62 | NA | NA | NA | NA |
| Breslow 2004 | Intervention: 60.10 | NR | Base: 61.30 | NR | NA | NA | NA | NA |
| Collins 2017 | NR | VICU: 60 | NR | SICU: 58 | NA | NA | NA | NA |
| Davis 2017 | After TCC: 48.20 | NR | Before TCC: 44.80 | NR | NA | NA | NA | NA |
| Forni 2010 | After period: 61.80 | NR | Before period: 63.10 | NR | NA | NA | NA | NA |
| Kahn 2016 | Preperiod, adopting: 77.90 | NR | Postperiod, adopting: 78.00 | NR | Preperiod, non-adopting: 77.80 | NR | Postperiod, non-adopting: 78.00 | NR |
| Lilly 2011 | Tele-ICU Group: 64.00 | NR | Preintervention Group: 62.00 | NR | NA | NA | NA | NA |
| Lilly 2014 | ICU TM Group: 62.70 | NR | Control Group: 62.70 | NR | NA | NA | NA | NA |
| McCambridge 2010 | HITB-RIC Group: 64.40 | NR | Control Group: 65.00 | NR | NA | NA | NA | NA |
| Morrison 2010 | Wave One: 65.10 | NR | Wave two: 64.25 | NR | Baseline: 64.40 | NR | NA | NA |
| Nassar 2014^a^ | Intervention Pre-TM Period: 66.20 | NR | Intervention, Post-TM Period: 66.50 | NR | Control, Pre-TM Period: 67.50 | NR | Control, Post-TM Period: 67.70 | NR |
| O'Shea 2022^a^ | Frequent, medium, and Infrequent interaction Tele-CC facilities: 66.80 | NR | Non-Tele-CC facilities: 67.80 | NR | NA | NA | NA | NA |
| Udeh 2022 | NR | ICU-TM: 70 | NR | No ICU-TM: 69 | NA | NA | NA | NA |
| VanGent 2018 | NR | NR | NR | NR | NA | NA | NA | NA |
| Willmitch 2012 | NR | NR | NR | NR | NA | NA | NA | NA |
| Fortis 2014 | 2012: 58.20 | NA | 2011: 58.10 | NR | NA | NA | NA | NA |
| Lilly 2017 | ICU TM: 62.40 | NR | Logistic Center: 62.70 | NR | Pre-ICU TM: 62.20 | NR | NA | NA |
| Panlaqui 2017 | Tele-ICU: 68.00 | NR | Baseline: 64.00 | NR | NA | NA | NA | NA |
| Rosenfeld 2000 | Intervention: 61.00 | NR | Baseline 1: 62.00 | NR | Baseline 2: 60.00 | NR | NA | NA |
| Sadaka 2013 | Tele-ICU: 67.10 | NR | Preintervention: 66.10 | NR | NA | NA | NA | NA |
| Thomas 2009 | Total After: 59.30 | NR | Total Before: 60.20 | NR | NA | NA | NA | NA |
| Marx 2022 | Intervention with TM: 72.14 | NR | Preintervention phase: 69.25 | NR | NA | NA | NA | NA |
| Pannu 2017 | NR | Postimplementation: 66 | NR | Preimplementation: 66 | NA | NA | NA | NA |
| Spies 2023 | Intervention: 67.25 | NR | Control: 68.74 | NR | NA | NA | NA | NA |
| Fortis 2018^a^ | ICU TM, Post-TM: 65.92 | NR | Non-TM, Pre-TM: 65.74 | NR | ICU TM, Pre-TM: 65.45 | NR | Non-TM, Post-TM: 66.36 | NR |
| Pereira 2024 | Intervention, TCC: 60.80 | NR | Usual care: 61.20 | NR | NA | NA | NA | NA |

**Abbreviations:** Health information technology bundle (HITB), intensive care unit (ICU), not applicable (NA), not reported (NR), remote intensive coverage (RIC), tele-critical care (TCC), telemedicine (TM).

**Footnotes:**

**^a^**Studies used the same population pool for analyses.
