# Supplementary material for: Telemedicine in adult intensive care: A systematic review of patient-relevant outcomes and methodological considerations
Source: PLOS Digit Health. 2025 Dec 15;4(12):e0001126. doi: 10.1371/journal.pdig.0001126 (PMC12704867; doi:10.1371/journal.pdig.0001126)
Supplement: S6 Table — (DOCX) [file pdig.0001126.s009.docx]

**Table 6: Number and percentages of males per group of all included studies.**

| Study ID | Group 1: No. of males (n) | Group 1: No. of males (%) | Group 2: No. of males (n) | Group 2: No. of males (%) | Group 3: No. of males (n=) | Group 3: No. of males (%) | Group 4: No. of males (n) | Group 4: No. of males (%) |
| --- | --- | --- | --- | --- | --- | --- | --- | --- |
| Boyle 2023 | Post-TCC: 7,480 | Post-TCC: 60 | Pre-TCC: 1,396 | Pre-TCC: 57.5 | NA | NA | NA | NA |
| Breslow 2004 | Intervention: 370 | NR | Base: 782 | NR | NA | NA | NA | NA |
| Collins 2017 | VICU: 518 | VICU: 50 | SICU: 4,235 | SICU: 61 | NA | NA | NA | NA |
| Davis 2017 | NR | NR | NR | NR | NA | NA | NA | NA |
| Forni 2010 | After period: 620 | After period: 58 | Before period: 635 | Before period: 59 | NA | NA | NA | NA |
| Kahn 2016 | Preperiod, adopting: 72,580 | Preperiod, adopting: 49.2 | Postperiod, adopting:71,616 | Postperiod, adopting: 49.3 | Preperiod, non-adopting: 205590 | Preperiod, non-adopting: 49 | Postperiod, non-adopting: 202,895 | Postperiod, non-adopting: 49.3 |
| Lilly 2011 | Tele-ICU Group: 2,701 | Tele-ICU Group: 57 | Preintervention Group: 874 | Preintervention Group: 57 | NA | NA | NA | NA |
| Lilly 2014 | ICU TM Group: 58,192 | ICU TM Group: 54.2 | Control Group: 6,261 | Control Group: 54.2 | NA | NA | NA | NA |
| McCambridge 2010 | HITB-RIC Group: 481 | HITB-RIC Group: 50.2 | Control Group: 478 | Control Group: 50.1 | NA | NA | NA | NA |
| Morrison 2010 | Wave one: 646 | Wave one: 50.94 | Wave two: 762 | Wave two: 53.29 | Baseline: 773 | Baseline. 56.38 | NA | NA |
| Nassar 2014^a^ | Intervention, Pre-TM Period: 1,658 | Intervention, Pre-TM Period: 97.1 | Intervention, Post-TM Period: 1,607 | Intervention, Post-TM Period: 97.6 | Control, Pre-TM Period: 1,611 | Control, Pre-TM Period: 96.8 | Control, Post-TM Period: 1,857 | Control, Post-TM Period: 96.7 |
| O'Shea 2022^a^ | Frequent, medium, and Infrequent interaction Tele-CC facilities: 78,308 | Frequent, medium, and Infrequent interaction Tele-CC facilities: 96.3 | Non-Tele-CC facilities: 225,731 | Non-Tele-CC facilities: 95.8 | NA | NA | NA | NA |
| Udeh 2022 | NR | ICU-TM: 56.8 | NR | No ICU-TM: 57.3 | NA | NA | NA | NA |
| VanGent 2018 | NR | NR | NR | NR | NA | NA | NA | NA |
| Willmitch 2012 | NR | NR | NR | NR | NA | NA | NA | NA |
| Fortis 2014 | 2012: 3,203 | 2012: 52.5 | 2011: 3,187 | 2011: 52.6 | NA | NA | NA | NA |
| Lilly 2017 | ICU TM: 8,362 | ICU TM: 57.5 | Logistic Center: 12,523 | Logistic Center: 55.9 | Pre-ICU TM: 8,262 | Pre-ICU TM: 57.9 | NA | NA |
| Panlaqui 2017 | Tele-ICU: 123 | Tele-ICU: 65 | Baseline: 202 | Baseline: 60 | NA | NA | NA | NA |
| Rosenfeld 2000 | NR | Intervention: 57 | NR | Baseline 1: 55 | NR | Baseline 2: 63 | NA | NA |
| Sadaka 2013 | Tele-ICU: 1,162 | Tele-ICU: 53 | Preintervention: 334 | Preintervention: 53 | NA | NA | NA | NA |
| Thomas 2009 | Total After: 1,115 | Total After: 52.9 | Total Before: 1,038 | Total Before: 51 | NA | NA | NA | NA |
| Marx 2022 | Intervention with TM: 1,415 | Intervention with TM: 49.6 | Preintervention phase: 1,920 | Preintervention phase: 46.8 | NA | NA | NA | NA |
| Pannu 2017 | Postimplementation: 94 | Postimplementation: 51.9 | Preimplementation: 89 | Preimplementation: 58.2 | NA | NA | NA | NA |
| Spies 2023 | Intervention: 571 | Intervention: 54.5 | Control: 228 | Control: 55.1 | NA | NA | NA | NA |
| Fortis 2018^a^ | ICU TM, Post-TM: 33,961 (admissions) | ICU TM, Post-TM: 96.72 | Non-TM, Pre-TM: 185,911 | Non-TM, Pre-TM: 96.5 | ICU TM, Pre-TM: 59,688 (admissions) | ICU TM, Pre-TM: 96.05 | Non-TM, Post-TM: 253,552 | Non-TM, Post-TM: 96.18 |
| Pereira 2024 | Intervention, TCC: 4,243 | Intervention, TCC: 56.8 | Usual care: 4,212 | Usual care: 54.3 | NA | NA | NA | NA |

**Abbreviations:** Health information technology bundle (HITB), intensive care unit (ICU), not applicable (NA), not reported (NR), remote intensive coverage (RIC), tele-critical care (TCC), telemedicine (TM).

**Footnotes:**

**^a^**Studies used the same population pool for analyses.
