# Supplementary material for: Telemedicine in adult intensive care: A systematic review of patient-relevant outcomes and methodological considerations
Source: PLOS Digit Health. 2025 Dec 15;4(12):e0001126. doi: 10.1371/journal.pdig.0001126 (PMC12704867; doi:10.1371/journal.pdig.0001126)
Supplement: S7 Table — (DOCX) [file pdig.0001126.s010.docx]

**Table 7: Risk of bias results for (sw-)cRCTs assessed with RoB 2.**

| **Study ID** | **Outcome** | **Numerical result** | **D1a** | **D1b** | **D2** | **D3** | **D4** | **D5** | **Overall** |
| --- | --- | --- | --- | --- | --- | --- | --- | --- | --- |
| **Spies 2023** | ICU mortality | RR 0.89 (95% CI 0.54 – 1.46)^1^ | 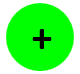 | 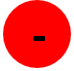 | 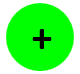 | 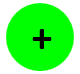 | 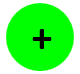 | 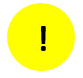 | 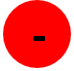 |
| **Spies 2023** | Overall mortality at 180 days | RR 1.02 (95% CI 0.76 – 1.36)^1^ | 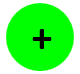 | 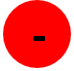 | 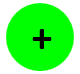 | 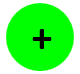 | 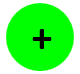 | 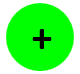 | 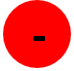 |
| **Spies 2023** | Quality of life | MD -2.71 (95% CI 6.95 fewer to 1.53 more) | 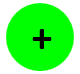 | 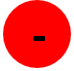 | 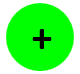 | 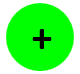 | 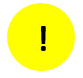 | 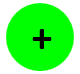 | 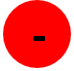 |
| **Spies 2023** | ICU LOS | Median [IQR] 6 [4–13] days (telemedicine) vs 5 [3–11] days (SoC)^1^ | 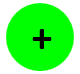 | 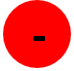 | 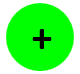 | 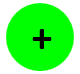 | 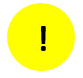 | 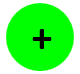 | 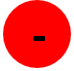 |
| **Marx 2022** | ICU mortality | RR 2.29 (95% CI 1.51 – 3.48)^1^ | 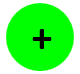 | 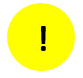 | 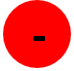 | 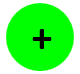 | 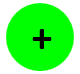 | 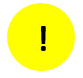 | 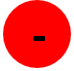 |
| **Pereira 2024** | ICU LOS | Mean (SD) 8.1 (10) days (telemedicine) vs 7.1 (9) days (SoC); MD 1 day more, 95% CI 0.04 fewer to 1.96 more | 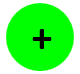 | 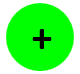 | 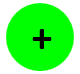 | 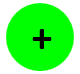 | 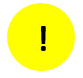 | 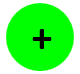 | 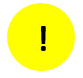 |
| **Pereira 2024** | ICU mortality | RR 1.09 (95% CI 0.94 – 1.26) | 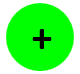 | 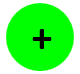 | 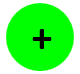 | 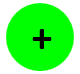 | 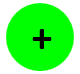 | 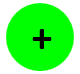 | 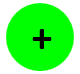 |

**Abbreviations:** cluster randomized controlled trial (cRCT), intensive care unit (ICU), interquartile range (IQR), length of stay (LOS), mean difference (MD), risk of bias (RoB), risk ratio (RR), odds ratio (OR), standard deviation (SD), standard of care (SoC), stepped-wedge cluster randomized controlled trial (sw-cRCT).

**Footnotes:**

^1^Bias for missing adjustment of time trends.

| D1a | Randomisation process |
| --- | --- |
| D1b | Timing of identification or recruitment of participants |
| D2 | Deviations from the intended interventions |
| D3 | Missing outcome data |
| D4 | Measurement of the outcome |
| D5 | Selection of the reported result |


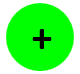


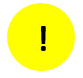
 Low risk


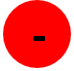
 Some concerns

High risk
