# Supplementary material for: Telemedicine in adult intensive care: A systematic review of patient-relevant outcomes and methodological considerations
Source: PLOS Digit Health. 2025 Dec 15;4(12):e0001126. doi: 10.1371/journal.pdig.0001126 (PMC12704867; doi:10.1371/journal.pdig.0001126)
Supplement: S8 Table — (DOCX) [file pdig.0001126.s011.docx]

**Table 8: Risk of bias results for NRSIs assessed with ROBINS-I.**

| **Study ID** | **Outcome** | **Numerical result** | **D1** | **D2** | **D3** | **D4** | **D5** | **D6** | **D7** | **Overall** |
| --- | --- | --- | --- | --- | --- | --- | --- | --- | --- | --- |
| **Thomas 2009** | ICU mortality | RR 0.88 (95% CI 0.71 – 1.08) | Moderate | Serious | Low | Serious | Low | Low | Moderate | Serious |
| **Nassar 2014** | ICU mortality | OR 1.07 (95% CI 0.6 – 1.9) | Moderate | Critical | Low | Serious | Critical | Low | Moderate | Critical |
| **Sadaka 2013** | ICU mortality | OR 0.46 (95% CI 0.32 – 0.66) | Moderate | Serious | Low | Serious | Low | Low | Moderate | Serious |
| **Lilly 2011** | ICU mortality | OR 0.37 (95% CI 0.28 – 0.49) | Moderate | Low | Low | Low | Low | Low | Moderate | Moderate |
| **Lilly 2011** | ICU mortality | OR 0.58 (95% CI 0.41 – 0.83) | Serious | Low | Low | Low | Low | Moderate | Moderate | Serious |
| **Lilly 2014** | ICU mortality | HR 0.74 (95 % CI 0.68 – 0.79) | Moderate | Critical | Low | Serious | Low | Low | Moderate | Critical |
| **Panlaqui 2017** | ICU mortality | RR 0.6 (95% CI 0.1 – 3.1) | Moderate | Critical | Low | Serious | Low | Low | Moderate | Critical |
| **Nassar 2014** | Overall mortality at longest follow-up (30 days) | OR 1.1 (95% CI 0.82 – 1.47) | Moderate | Critical | Low | Serious | Critical | Serious | Moderate | Critical |
| **Kahn 2016** | Overall mortality at longest follow-up (90 days) | RR 1.04 (95% CI 1.03 – 1.06) | Moderate | Critical | Low | Serious | Low | Low | Moderate | Critical |
| **Nassar 2014** | ICU LOS | OR 1.02 (95% CI 0.95 – 1.11) | Moderate | Critical | Low | Serious | Critical | Moderate | Moderate | Critical |
| **Lilly 2011** | ICU LOS | HR 1.26 (95% CI 1.17 – 1.36) | Moderate | Low | Low | Low | Low | Low | Moderate | Moderate |
| **Willmitch 2012** | ICU LOS | MD 0.56, (95% CI 0.36 – 0.76) | Serious | Low | Low | Low | Low | Moderate | Moderate | Serious |

**Abbreviations:** Hazard ratio (HR), length of stay (LOS), mean difference (MD), odds ratio (OR), non-randomized study of intervention (NRSI), risk ratio (RR), Risk Of Bias In Non-randomized Studies - of Interventions (ROBINS-I), intensive care unit (ICU), standard of care (SoC).

| D1a | Confounding | D5 | Missing data |
| --- | --- | --- | --- |
| D2 | Selection bias | D6 | Outcome measurement |
| D3 | Intervention classification | D7 | Selection of reported results |
| D4 | Deviations from intended intervention |  |  |
