# Supplementary material for: Telemedicine in adult intensive care: A systematic review of patient-relevant outcomes and methodological considerations
Source: PLOS Digit Health. 2025 Dec 15;4(12):e0001126. doi: 10.1371/journal.pdig.0001126 (PMC12704867; doi:10.1371/journal.pdig.0001126)
Supplement: S9 Table — (DOCX) [file pdig.0001126.s012.docx]

**Table 9: Secondary outcome hospital mortality; data from two (sw-)cRCTs and nine NRSIs.**

| Study ID | Intervention arm: no. of events/no. of participants analysed | Control arm: no. of events/no. of participants analysed | OR or RR (95% CI) | Adjustment for |
| --- | --- | --- | --- | --- |
| Pereira 2024 | 3,106/7,471 | 3,119/7,759 | OR 0.93 (95% CI 0.78 – 1.12) | SAPS-3 score, type of ICU admission, invasive mechanical ventilation at ICU admission, number of ICU beds, region where the ICU was located, baseline category of ICU performance from the SMR and SRU matrix, and batch of randomization |
| Boyle 2023 | 1,2479/1,186 | 2,429/260 |  | risk-adjusted (multiplying the corresponding SMR and standardized HLOS ratio for that period with the group-wise  pooled expected mortality and pooled expected  HLOS, respectively) |
| Lilly 2011 | 4,761/NR | 1,529/NR | OR 0.4 (95% CI 0.31 – 0.52) | differences in acuity score, admission source, admission ICU, time after enrollment of first case in group, and other predictive factors including laboratory values and physiological measurements |
| Lilly 2011 | 4,761/NR | 1,529/NR | OR 0.54 (95% CI 0.39 – 0.73) | adherence to best practices and lower rates of complications |
| Lilly 2014 | NR | NR | HZ 0.84 (95% CI 0.78 – 0.89) | APACHE IV score, age, hospital or ICU identifier (as a random effect), admission source, primary admission diagnosis, operative status, time from start of study enrollment, heart rate, admission and highest creatinine values, respiratory rate, admission hematocrit value, BUN, WBC count, Glasgow Coma Score, prothrombin time, anion gap, urine output (in the first 24 h), base excess, and total bilirubin and albumin values |
| Nassar 2014^a^ | 1,647/NR | 1,708/NR | OR 1.33 (95% CI 0.86 – 2.07) | patient demographics, comorbid illness, primary conditions at ICU admission and the most abnormal laboratory values during 24 h surrounding ICU admission, categorized to the APACHE III scoring method |
| O’Shea 2022^a^ | 8,575/NR | NR/NR | OR 1.11 (95% CI 0.64 – 1.57) | age, gender, race, rural residency, primary diagnosis, illness severity |
| Willmitch 2012 | 5,781/NR | 6,504/NR | RR 0.77 (95% CI 0.87 – 0.87) | severity of illness |
| Panlaqui 2017^b^ | 188/NR | 337/NR | RR 0.41 (95%CI 0.15 – 1.13) | age and APACHE II scores |
| Panlaqui 2017^c^ | 188/NR | 337/NR | RR 0.5 (95% Ci 0.2 – 1.2) | age and APACHE II scores |
| Sadaka 2013 | 1,53/2,193 | 56/630 | OR 0.76 (96% CI 0.55 – 1) | severity-adjusted (APS and APACHE IV scores) |

**Abbreviations:** Acute Physiology And Chronic Health Evaluation (APACHE), antiphospholipid syndrome (APS), blood urea nitrogen (BUN), cluster randomized controlled trial (cRCT), hazard ratio (HR), hospital length of stay (HLOS), intensive care unit (ICU), length of stay (LOS), non-randomized study of intervention (NRSI), not reported (NR), odds ratio (OR), risk ratio (RR), standard deviation (SD), stepped-wedge cluster randomized controlled trial (sw-cRCT), white blood cells (WBC).

**Footnotes:**

**^a^**Studies used the same population pool for analyses.

^b^hospital mortality = all patients who died after discharge from ICU within the hospital.

^c^total mortality = all patients who died within the hospital, regardless of the ward.
