# Supplementary material for: Telemedicine in adult intensive care: A systematic review of patient-relevant outcomes and methodological considerations
Source: PLOS Digit Health. 2025 Dec 15;4(12):e0001126. doi: 10.1371/journal.pdig.0001126 (PMC12704867; doi:10.1371/journal.pdig.0001126)
Supplement: S10 Table — (DOCX) [file pdig.0001126.s013.docx]

**Table 10: Secondary outcome hospital LOS; data from one sw-cRCT and data from seven NRSIs.**

| Study ID | Intervention arm: no. of participants analysed | Control arm: no. of participants analysed | Intervention mean or MD (SD or 95% CI) | Control mean (SD or 95% CI) | RR / OR / HR (95% CI) | Adjustment for |
| --- | --- | --- | --- | --- | --- | --- |
| Marx 2022 | NR | 2,408 | 20.63 (95% CI 19.55 – 21.7) MD 4.61 (95% CI 3.32 – 5.91) | 16.26 (95% CI 15.65 – 16.87) | NR | treating hospital, patient age, and SOFA score |
| Boyle 2023 | 12,479 | 2,429 | 12.4 (SD 12.06) | 11.8 (SD 11.5) | NR | risk-adjusted (multiplying the corresponding SMR and standardized HLOS ratio for that period with the group-wise pooled expected mortality and pooled expected HLOS, respectively) |
| Lilly 2011 | 4,761 | 1,529 | NR | NR | HR 1.44 (95% CI 1.33 – 1.56) | differences in acuity score, admission source, admission ICU, time after enrollment of first case in group, and other predictive factors including laboratory values and physiological measurements |
| Nassar 2014^a^ | 1,647 | 1,708 | NR | NR | OR 1.03 (95% CI 0.96 – 1.11) | patient demographics, comorbid illness, primary conditions at ICU admission and the most abnormal laboratory values during 24 h surrounding ICU admission, categorized to the APACHE III scoring method |
| O’Shea 2022^a^ | NR | NR | NR | NR | RR 0.11 (95% CI 0.01 – 1.98) | age, gender, race, rural residency, primary diagnosis, illness severity |
| Willmitch 2012 | 5,781 | 6,507 | 10.6 (95% CI 9.8 – 10.53) MD 1.7 (95% CI 1.202 – 2.198) | 11.86 (95% CI 11.52 – 12.21) | NR | severity of illness |
| Sadaka 2013 | 2,193 | 630 | 6.2 (SD 7.4) | 5.2 (SD 6.1) | NR | severity-adjusted (APS and APACHE IV scores) |
| Sadaka 2013 | NR | NR | NR | NR | HR 1.3 (95% CI 1.25 – 1.35) | severity-adjusted (APS and APACHE IV scores) |

**Abbreviations:** Acute Physiology And Chronic Health Evaluation (APACHE), antiphospholipid syndrome (APS), hazard ratio (HR), hospital length of stay (HLOS), intensive care unit (ICU), length of stay (LOS), mean difference (MD), non-randomized study of intervention (NRSI), not reported (NR), odds ratio (OR), risk ratio (RR), standard deviation (SD), Sequential Organ Failure Assessment (SOFA), standardized mortality ratio (SMR), stepped-wedge cluster randomized controlled trial (sw-cRCT).

**Footnotes:**

**^a^**Studies used the same population pool for analyses.
