# Supplementary material for: Telemedicine in adult intensive care: A systematic review of patient-relevant outcomes and methodological considerations
Source: PLOS Digit Health. 2025 Dec 15;4(12):e0001126. doi: 10.1371/journal.pdig.0001126 (PMC12704867; doi:10.1371/journal.pdig.0001126)
Supplement: S11 Table — (DOCX) [file pdig.0001126.s014.docx]

**Table 11: Secondary outcome adherence to best practice guidelines; data from one sw-cRCT (Marx 2022); adjusted for treating hospital, patient age, and SOFA score.**

| Study ID | Guidelines | Intervention arm:  no. of events /  no. of participants analysed | Control arm:  no. of events /  no. of participants analysed | Odds ratio (95% CI) |
| --- | --- | --- | --- | --- |
| Marx 2022 | adherence to the 3-hour sepsis compliance bundles | 18/23 | 35/66 | NR |
| Marx 2022 | adherence to the 6- hour sepsis compliance bundles | 15/18 | 20/57 | 14.245 (95% CI 3.141 – 85.424) |
| Marx 2022 | overall sepsis bundle adherence | 17/23 | 17/66 | 7.739 (95% CI 2.379 – 28.026) |
| Marx 2022 | imperatively start antimicrobial treatment and remove the focus on Staphylococcus aureus bloodstream infection. | 43/94 | 15/92 | 4.004 (95% CI 1.828 – 9.202) |
| Marx 2022 | critically ill patients with signs of infection need early appropriate antibiotic therapy | 18/20 | 29/59 | 6.822 (95% CI 1.271 – 56.607) |
| Marx 2022 | prefer oral formulations of highly bioavailable antimicrobials whenever possible | 6/24 | 19/88 | 1.135 (95% CI 0.179 – 7.493) |
| Marx 2022 | do not prolong prophylactic administration of antibiotics in patients after they have left the operating room | 25/31 | 110/129 | 1.744 (95% CI 0.326 – 12.861) |
| Marx 2022 | do not treat an elevated C‐reactive protein or procalcitonin level with antibiotics in patients without signs of infection | 125/137 | 531/590 | 1.463 (95% CI 0.666 – 3.416) |
| Marx 2022 | ARDS compliance, mild ARDS patients | 19/161 | 16/217 | 2.355 (95% CI 1.023 – 5.516) |
| Marx 2022 | ARDS compliance, moderate ARDS patients | 20/158 | 26/219 | 1.214 (95% CI 0.595 – 2.462) |
| Marx 2022 | ARDS compliance, severe ARDS patients | 8/57 | 10/61 | 0.555 (95% CI 0.139 – 2.07) |

**Abbreviations:** Acute respiratory distress syndrome (ARDS), confidence interval (CI), not reported (NR), Sequential Organ Failure Assessment (SOFA), stepped-wedge cluster randomized controlled trial (sw-cRCT).
