# Supplementary material for: Telemedicine in adult intensive care: A systematic review of patient-relevant outcomes and methodological considerations
Source: PLOS Digit Health. 2025 Dec 15;4(12):e0001126. doi: 10.1371/journal.pdig.0001126 (PMC12704867; doi:10.1371/journal.pdig.0001126)
Supplement: S12 Table — (DOCX) [file pdig.0001126.s015.docx]

**Table 12: Secondary outcome disease specific effects; data from three (sw-)cRCTs.**

| Study ID | Disease specific effects | Intervention arm:  no. of events /  no. of participants analysed | Control arm:  no. of events /  no. of participants analysed | Intervention mean (SD or 95% CI) | Control mean (SD) | Adjusted for |
| --- | --- | --- | --- | --- | --- | --- |
| Marx 2022 | antibiotic days | NR/NR | NR/1,213 | 0.752 (95% CI 0.73 – 0.77) | 0.761 (95% CI 0.74 – 0.79)^1^ | adjusted for treating hospital, patient age, and SOFA score |
| Spies 2023 | received mechanical ventilation | 779/1,048 | 251/414 | NR | NR | not adjusted |
| Spies 2023 | duration of mechanical ventilation of mechanically ventilated patients (hours) | NR/1,048 | NR/414 | 212.02 (SD 320) | 196.17 (SD 343.75) | not adjusted |
| Pereira 2024 | ventilator-free days at 28 days | NR/7,471 | NR/7,759 | 8.2 (SD 11) | 9.2 (SD 11) | SAPS-3 score, type of ICU admission, invasive mechanical ventilation at ICU admission, number of ICU beds, region where the ICU was located, baseline category of ICU performance from the SMR and SRU matrix, and batch of randomization |

**Abbreviations:** Cluster randomized controlled trial (cRCT), confidence interval (CI), not reported (NR), intensive care unit (ICU), Sequential Organ Failure Assessment (SOFA), Simplified Acute Physiology Score (SAPS), standard deviation (SD), standardized mortality ratio (SMR), standardized resource use (SRU), stepped-wedge cluster randomized controlled trial (sw-cRCT).
