# Supplementary material for: Telemedicine in adult intensive care: A systematic review of patient-relevant outcomes and methodological considerations
Source: PLOS Digit Health. 2025 Dec 15;4(12):e0001126. doi: 10.1371/journal.pdig.0001126 (PMC12704867; doi:10.1371/journal.pdig.0001126)
Supplement: S13 Table — (DOCX) [file pdig.0001126.s016.docx]

**Table 13: Secondary outcome transfer rate; data from one sw-cRCT and data from one NRSI.**

| Study ID | Intervention arm: no. of events/no. of participants analysed | Control arm: no. of events/no. of participants analysed | Odds ratio (95% CI) | Adjustment for |
| --- | --- | --- | --- | --- |
| Marx 2022 | 101/857 | 86/1,965 | 2.093 (95% CI 2.012 – 4.186) | treating hospital, patient age, and SOFA score |
| O’Shea 2022 | NR/8,575 | NR/NR | 3.1 (95% CI 0.6 – 5.59) | age, gender, race, rural residency, primary diagnosis, illness severity |

**Abbreviations:** Confidence interval (CI), non-randomized study of intervention (NRSI), not reported (NR), Sequential Organ Failure Assessment (SOFA), stepped-wedge cluster randomized controlled trial (sw-cRCT).
