# Supplementary material for: Telemedicine in adult intensive care: A systematic review of patient-relevant outcomes and methodological considerations
Source: PLOS Digit Health. 2025 Dec 15;4(12):e0001126. doi: 10.1371/journal.pdig.0001126 (PMC12704867; doi:10.1371/journal.pdig.0001126)
Supplement: S14 Table — (DOCX) [file pdig.0001126.s017.docx]

**Table 14: Secondary outcome fulfilment of process and quality indicators; data from one sw-cRCT; adjusted for time, with random intercepts for patient, and centre levels.**

| Study ID | No. of participants analysed/no. of participants enrolled | DIVI quality indicators (QIs) | Absolute difference (99.375% CI) | Odds ratio (99.375% CI) |
| --- | --- | --- | --- | --- |
| Spies 2023 | 1,512/1,553 | QI 1 (daily multi-professional and interdisciplinary clinical visits with documentation of daily goals) | 0.009 (− 0.009 – 0.026) | 1.606 (0.78 – 3.309) |
| Spies 2023 | 1,512/1,553 | QI 2 (management of sedation, analgesia and delirium) | 0.245 (0.06 – 0.43) | 5.328 (3.395 – 8.358) |
| Spies 2023 | 1,512/1,553 | QI 3 (patient-adapted ventilation) | 0.026 (0.001 – 0.051) | 2.248 (1.198 – 4.217) |
| Spies 2023 | 1,512/1,553 | QI 4 (early weaning from invasive ventilation) | 0.005 (− 0.001 – 0.011) | 9.049 (2.707 – 30.247) |
| Spies 2023 | 1,512/1,553 | QI 6 (measures for infection management) | 0.005 (− 0.001 – 0.011) | 4.397 (1.482 – 13.037) |
| Spies 2023 | 1,512/1,553 | QI 7 (early enteral nutrition) | 0.036 (0 – 0.072) | 1.579 (1.032 – 2.416) |
| Spies 2023 | 1,512/1,553 | QI 8 (documentation of  structured patient and  family communication) | 0.124 (0.017 – 0.231) | 6.787 (3.976 – 11.589) |
| Spies 2023 | 1,512/1,553 | QI 9 (early mobilization) | 0.055 (− 0.008 – 0.119) | 3.161 (2.16 – 4.624) |

**Abbreviations:** Confidence interval (CI), Deutsche Interdisziplinäre Vereinigung für Intensiv- und Notfallmedizin (DIVI), quality indicator (QI), stepped-wedge cluster randomized controlled trial (sw-cRCT).
